# Trisubstituted Pyrrolinones as Small-Molecule Inhibitors Disrupting the Protein–RNA Interaction of LIN28 and *Let-7*

**DOI:** 10.1021/acsmedchemlett.0c00546

**Published:** 2021-03-01

**Authors:** Lydia Borgelt, Fu Li, Pascal Hommen, Philipp Lampe, Jimin Hwang, Georg L. Goebel, Sonja Sievers, Peng Wu

**Affiliations:** † Chemical Genomics Centre, Max Planck Institute of Molecular Physiology, Dortmund 44227, Germany; ‡ Department of Chemical Biology, Max Planck Institute of Molecular Physiology, Dortmund 44227, Germany; § Compound Management and Screening Center, Dortmund 44227, Germany

**Keywords:** Protein−RNA interaction, RNA-binding protein, Small molecule, LIN28 inhibitor, Substituted pyrrolinone

## Abstract

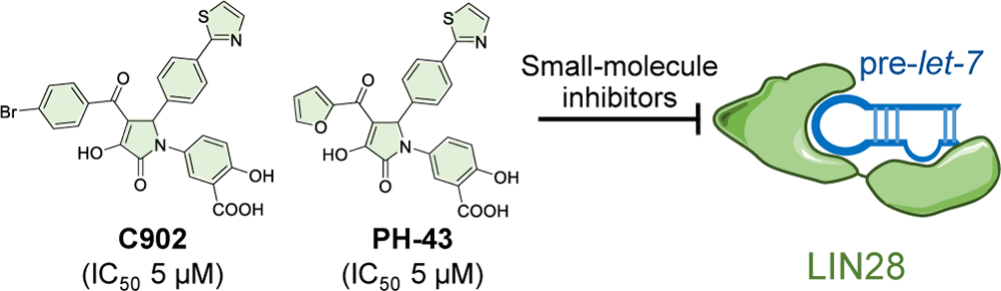

Modulation of protein–RNA interaction (PRI) using small molecules is a promising strategy to develop therapeutics. LIN28 is an RNA-binding protein that blocks the maturation of the tumor suppressor *let-7* microRNAs. Herein, we performed a fluorescence polarization-based screening and identified trisubstituted pyrrolinones as small-molecule inhibitors disrupting the LIN28–*let-7* interaction. The most potent compound C902 showed dose-dependent inhibition in an EMSA validation assay, enhanced thermal stability of the cold shock domain of LIN28, and increased mature *let-7* levels in JAR cells. The structure–activity relationship study revealed key structural features contributing to either PRI inhibition or stabilization of protein–protein interaction (PPI). The pyrrolinones identified in this study not only represent a new class of LIN28-binding molecules that diversify the limited available LIN28 inhibitors but also represent the first examples of small molecules that showed substituent-dependent PRI inhibitory and PPI activating activities.

Small molecules targeting protein–RNA interaction (PRI) hold great potential to be therapeutic candidates or biological probes given the diverse cellular events regulated by the interaction between RNA and RNA-binding proteins (RBPs).^1^ RNA metabolism is regulated by various RBPs,^2^ whose alteration has been associated with several human diseases.^3−5^ There are increasing numbers of reports on small-molecule RNA binders,^6−11^ whereas only limited examples of small molecules targeting RBPs are currently available.^12^

The microRNA (miRNA)-binding protein LIN28, which includes two human isoforms LIN28A and LIN28B,^13,14^ modulates the biogenesis of the *let-7* family miRNAs.^15,16^ More specifically, LIN28 binds to both the transcribed primary *let-7* (pri-*let-7*) and the Drosha-processed precursor *let-7* (pre-*let-7*), leading to reduced expression level of mature *let-7* by blockage of Drosha- and Dicer-mediated processing of pri-*let-7* and pre-*let-7*, respectively, and degradation of pre-*let-7* via the recruitment of terminal uridylyltransferases (Figure 1A).^17,18^ LIN28 features an N-terminal cold shock domain (CSD) and a C-terminal zinc knuckle domain (ZKD) containing two CCHC zinc finger motifs. CSD and ZKD are connected by a flexible linker that allows adapting to the stem lengths of different *let-7* family miRNAs. The CSD binds the stem loop region and the ZKD interacts with a GGAG motif in the bulge region of the precursor element (preE) of both pri-*let-7* and pre-*let-7* (Figure 1B).^19,20^ Additionally, LIN28 binds to mRNAs featuring a GGAGA motif within the loop structures.^21^ Targeting the LIN28–*let-7* interaction is of particular interest from a therapeutic perspective because, on the one hand, LIN28 is an oncogene that has been found to be overexpressed in ∼15% of primary human tumors and LIN28 overexpression has been associated with poor clinical prognosis.^22^ On the other hand, mature *let-7* plays an important role as a tumor-suppressing miRNA that downregulates MYC, RAS, and other oncogenes.^16,23^ Therefore, disruption of the Lin28–*let-7* interaction using small-molecule inhibitors to enhance *let-7* biogenesis and thus increase the level of mature *let-7* stands as a promising strategy to develop anticancer therapeutics. Furthermore, the LIN28–*let-7* interaction has been associated with the regulation of glucose metabolism^24^ and other human disease.^25^

**Figure 1:**
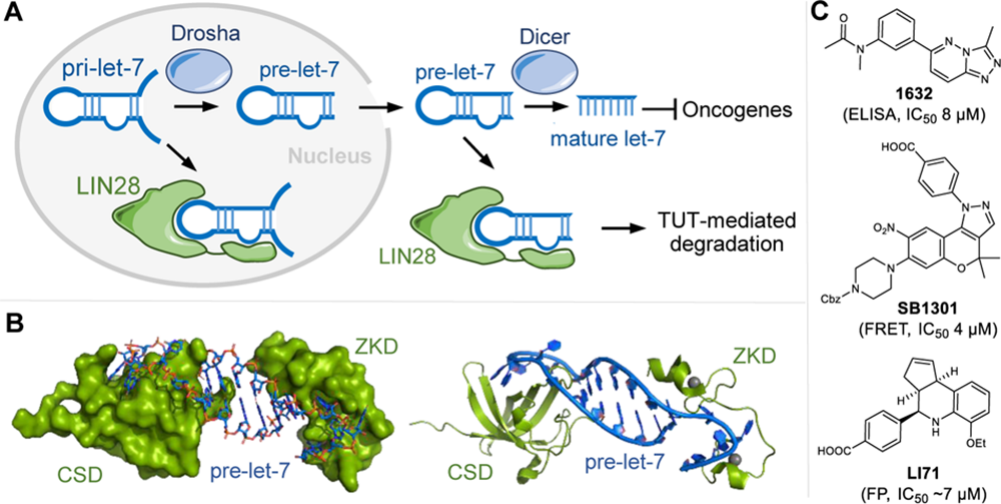
Targeting the protein–RNA interaction of LIN28–pre-*let-7*. (A) Simplified overview of the *let-7* biogenesis pathway. TUT, terminal uridylyltransferases. (B) Complex structure of human LIN28A and preE-*let-7f-1* (PDB ID: 5UDZ). The cold shock domain (CSD) and the zinc knuckle domain (ZKD) are shown in green (left, surface; right, ribbon), and the preE-*let-7f-1* is shown in blue. The flexible linker connecting the CSD and the ZKD domains is not resolved in this structure. (C) Representative LIN28 inhibitors 1632, SB1301, and LI71 and their reported IC_50_ values.

Small-molecule inhibitors targeting LIN28–*let-7* interaction were first reported in 2016,^26−28^ followed by a few recent reports (Figure 1C).^29−32^ The most potent inhibitors showed micromolar potency in in vitro assays, but suffered from low potency in cellular evaluations. Very limited structure–activity relationship (SAR) studies have been performed for even the most extensively studied class. Therefore, the identification of new classes of LIN28 inhibitors with scaffolds that are amenable for further structural optimization will likely lead to small molecules with improved inhibitory potency. Such inhibitors will be highly desired as biological probes or as potential candidates to develop anticancer therapeutics.

Herein, we performed the screening of a library containing structure-diverse molecules utilizing a fluorescence polarization (FP) assay to identify inhibitors disrupting the LIN28–*let-7* interaction (Figure 2A). A pilot screening of 1400 compounds led to the discovery of a pyrrolinone hit C902 that showed low micromolar inhibitory activity. A following electrophoretic mobility shift assay (EMSA) verified the dose-dependent inhibitory activity of the in-house resynthesized hit. Analysis of hit derivatives and analogues revealed PRI inhibitory SAR surrounding the pyrrolinone core scaffold and the association with the protein–protein interaction activating potency of this series of pyrrolinones.

**Figure 2:**
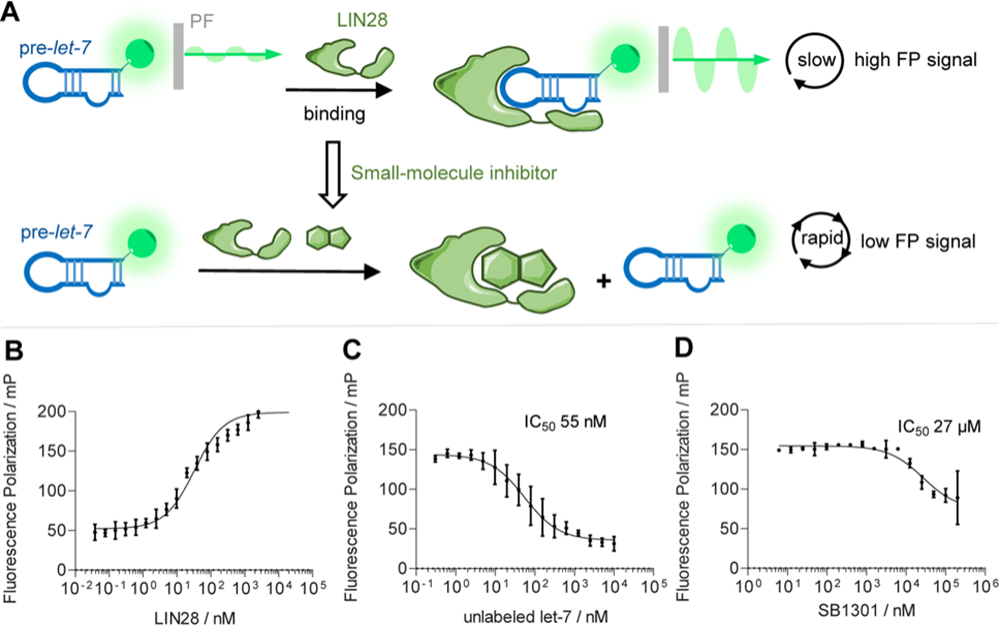
FP assay. (A) Small-molecule inhibitors disrupting the LIN28–*let-7* interaction led to low FP signal. PF, polarization emission filter. (B) FP assay of LIN28A (residues 16–187) titrated to 2 nM FAM-labeled preE-*let-7f-1* miRNA, three replicates, error bars indicate ± SD. LIN28A-bound preE*-let-7f-1* led to increased FP (mP). (C) Inhibition of the LIN28–*let-7* interaction using unlabeled preE-*let-7f-1*. (D) Inhibition of the LIN28–*let-7* interaction using the reported LIN28 inhibitor SB1301.

We used a FP assay to measure the binding between a truncated human LIN28A containing the CSD and ZKD and a FAM-labeled preE-*let-7f-1* miRNA (GGGGUAGUGAUUUUACCCUGUUUAGGAGAU-FAM) to identify inhibitors disrupting the LIN28–*let-7* interaction (Figure S1A). His-tagged LIN28A (residues 16–187) was purified using immobilized nickel affinity chromatography and the His-tag was cleaved by recombinant TEV protease to remove the potential influence induced by an artificial charge to LIN28A. In the FP assay, both His-tagged and untagged LIN28A (residues 16–187) were titrated into FAM-labeled preE-*let-7f-1* and FP was measured. Increased FP was observed for untagged LIN28A bound to preE-*let-7f-1* (Figure 2B) and His-tagged LIN28A (Figure S1B). Unlabeled preE-*let-7f-1* was used as a positive control in the FP assay with a tested IC_50_ of 55 nM, which is equivalent to the reported value (Figure 2C).^29^ Additionally, we synthesized the previously reported inhibitor SB1301 in-house and tested it in the FP assay (IC_50_: 27 μM, Figure 2D).^27^ In light of these results, the FP assay proved to be sufficiently robust and sensitive to be used for screening of small-molecule libraries for potential LIN28–*let-7* inhibitors.

We performed FP-based screening of an in-house library containing ∼15 000 natural product-inspired small molecules. Initial screening was performed for a pilot collection of 1400 compounds in the FP assay. Single concentration measurement at 30 μM was performed in triplicate. The unlabeled preE-*let-7f-1* miRNA was used as the positive control. FP and total fluorescence intensity (FI) were recorded. The *Z*′-factors were greater than 0.76, conveying high robustness and reliability of the screening results. Compounds that showed at least 50% FP inhibition but less than 300% FI of the control were grouped for the following purity check with a threshold of 85% by LC-MS. A following manual inspection led to an initial hit list of six heterocyclic small molecules. To confirm the hits from pilot screening, we tested the dose-dependent LIN28 inhibition in the FP assay for the six compounds, among which C902 showed micromolar inhibitory activity (Figure 3A,B).

**Figure 3:**
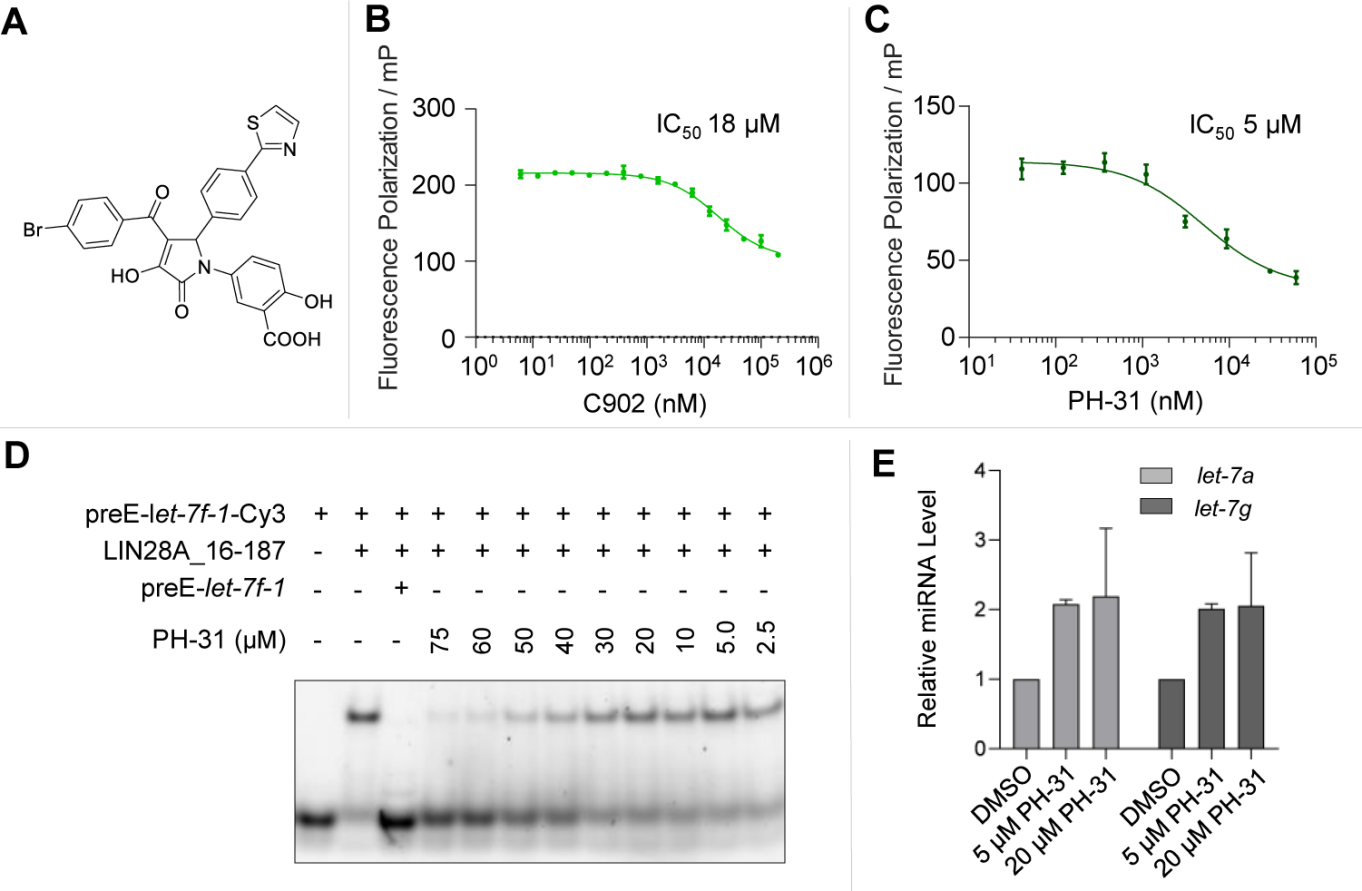
Screening and validation of inhibitors of the LIN28–*let-7* interaction. (A) Structure of the identified C902, which was resynthesized in-house as PH-31. (B) Inhibitory activity of C902 in the FP assay. (C) Inhibitory activity of PH-31 in the FP assay. (D) PH-31 showed dose-dependent inhibition of the LIN28–*let-7* interaction in EMSA. (E) Treatment of JAR cells with PH-31 led to increased levels of mature *let-7a* and *let-7g* quantified by RT-qPCR. Error bars indicate standard deviation of two independent measurements.

The pyrrolinone C902 was then resynthesized in-house as PH-31 with improved purity based on a previously reported synthetic route involving a three-component Doebner condensation of an amine, an aldehyde, and a dioxobutanoate component.^33^ After testing the IC_50_ of PH-31 in FP (Figure 3C), its LIN28–*let-7* inhibitory activity was verified in an EMSA, in which compounds that disrupt the formation of the LIN28–*let-7* complex can be identified with a readout orthogonal to FP. PH-31 showed dose-dependent micromolar inhibition of the LIN28–*let-7* complex (Figures 3D and S2), while in comparison the unlabeled preE–*let-7f-1* completely inhibited the formation of the protein–RNA complex at 500 nM. In contrast, an FP-inactive analogue C903 did not show activity in the EMSA (Figure S3). Furthermore, PH-31 showed concentration-dependent enhancement of the thermal stability of the CSD of LIN28A in a differential scanning fluorimetry assay (Figures S4 and S5). Additionally, the treatment of the human choriocarcinoma cell line JAR that endogenously expresses LIN28A and LIN28B with PH-31 led to increased levels of mature miRNAs *let-7a* and *let-7g* measured by RT-qPCR (Figure 3E). It is worth noting that the observed changes in cellular *let-7* level may be attributed to the polypharmacological nature of the trisubstituted pyrrolinones.

To further probe the inhibitory mechanism of the pyrrolinone hit C902/PH-31, molecular docking was performed using the reported structure of the LIN28–preE-*let-7f-1* complex.^34^ A scrutiny of the binding mode between preE-*let-7f-1* and LIN28 CSD did not reveal any deep pocket resembling that of the ATP-binding pocket that was targeted by many kinase inhibitors.^35^ Whereas there are a few surface pockets that may accommodate or engage C902 binding, such as the G7-binding surface dent, the G9-binding surface pocket, the A10-binding narrow groove, and the U14-binding wide groove (Figure 4A–C). Docking of C902 onto each of these surface sites were performed and evaluated based on docking scores, complementary of shape and charges, and predicted network of molecular interactions. Among all studied binding scenarios, the one in which C902 binds at the G9-binding surface pocket showed to be the most favored, as the K102 residue hooks into the V-shape structural moiety formed by the aryl substituents at the 1- and 5-positions of the pyrrolinone core (Figure 4D), together with the formation of an extensive network of molecular interactions (Figure 4E and F). The crucial salicylic acid moiety at the 1-position interacts with K102 via a π–cation interaction and forms hydrogen bonds with K98 and A101. The carbonyl at the 4-position may establish a salt bridge via its enol form with K78 and the benzoyl group interacts with H75 via a T-shaped π–π interaction. The thiazol-2-yl group at the 5-position stacks with F73, and the phenyl group at the 5-position interacts with K102 via a π–cation interaction. Additionally, the 2-carbonyl group forms a hydrogen bond with K98 and the 3-hydroxy groups may form a hydrogen bond with K78 through its ketone form. Docking results at the other sites are less convincing, since the G7-binding dent is too shallow to form a strong binding, the A10-binding groove is too narrow to fit C902, and the U14-binding groove is too wide to anchor C902.

**Figure 4:**
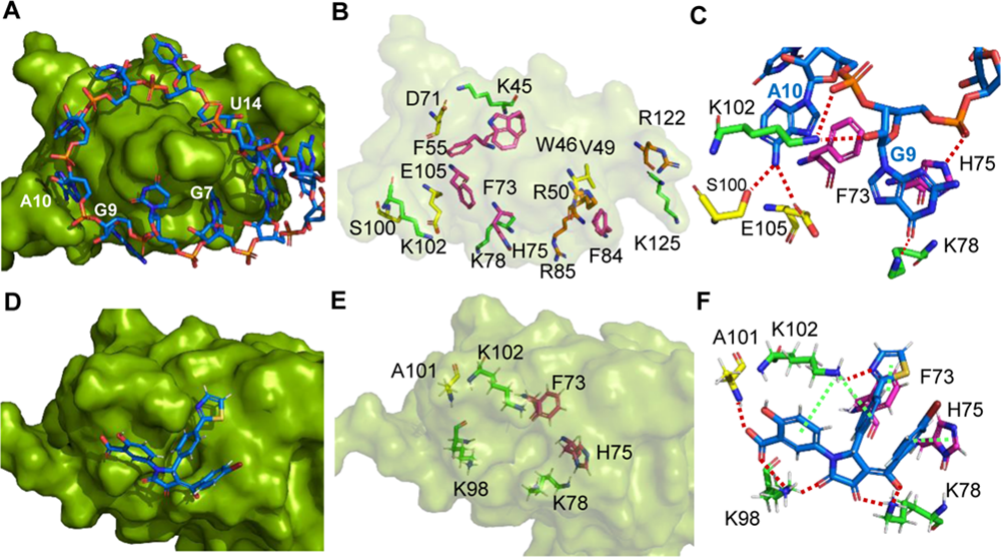
Binding analysis of pyrrolinone C902 to LIN28 based on the reported structure of the LIN28–preE-*let-7f-1* complex (PDB ID: 5UDZ). (A) Stem loop of preE-*let-7f-1* (shown in backbone comprised of blue ribose and orange phosphate) binding to the CSD of LIN28A (shown in green surface). (B) Key residues on the CSD that interact with preE-*let-7f-1*. Lysine residues are shown in green backbones, and arginine residues in pale-orange backbones. Residues including phenylalanine, tryptophan, and histidine that are involved in π–π stacking interaction are shown in magenta backbones. Other selected residues that interact with preE-*let-7f-1* primarily through hydrogen bond interactions are shown in yellow backbones. (C) Interactions of *let-7f-1* nucleotides with key residues at the G9–A10-binding groove. Hydrogen bond interactions are indicated by red dotted lines. (D) Docking of C902 (blue backbone) into the G9-binding groove on LIN28 CSD (green surface). (E) Key residues that interact with C902. K78, K98, and K102 are shown in green backbones; F73 and H75 that are involved in π–π stacking interactions are shown in magenta backbones; and A101 is shown in yellow backbone. (F) Interactions of C902 with key residues. Red dotted lines indicate hydrogen bonds and salt bridges. Green dashed lines indicate π–π stacking interactions and π–cation interactions.

To analyze the SAR surrounding the trisubstituted pyrrolinone scaffold of C902, we collected 28 compounds from the ∼15 000 natural product-inspired library, including 23 pyrrolinones with different substituents at each of the 1-, 4-, and 5-positions (C879–903, Table 1) and 5 pyrazoles that were obtained from the pyrrolinones with a further condensation step with hydrazine (C904–908, Table S1). The compounds were tested for their dose–response in the FP assay and the most potent compounds C879 and C880 with tested IC_50_ values below 30 μM were resynthesized in-house (PH-34 and PH-39, respectively), together with another seven compounds there were newly designed and synthesized to complement the SAR analysis (PH-series, Table 1, Figure S6, Tables S2–S4). Taken together, the FP dose–response results showed the crucial role of the salicylic acid moiety at the 1-position of the pyrrolinone core, as either removal of the 4-hydroxy group or the 3-carboxylic acid or replacement of the 3-carboxylic acid with a 4-carboxylic acid reduced activity or led to loss of activity. The benzoyl substituent at the 4-position of the pyrrolinone core can be tolerated with a 4-bromo or 4-methoxy group. Due to limited substitution patterns at the phenyl group at the 5-position, further structural modifications need to be performed to determine the SAR at this position. All tested pyrazoles were inactive in the FP assay. It is worth noting that the furan-2-carbonyl analogue PH-43 showed equivalent potency with that of C902 with an IC_50_ of 5 μM.

**Table 1:**
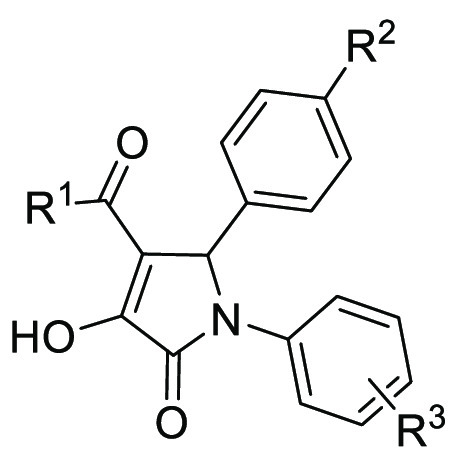
LIN28–*let-7* Inhibitory Activity of Trisubstituted Pyrrolinones

compd ID	R^1^	R^2^	R^3^	IC_50_ (μM)^a^
C879/PH-34	phenyl	thiazol-2-yl	3-COOH and 4-OH	12^b^*^,^*^d^
C880/PH-39	phenyl	COOH	3-COOH and 4-OH	6^b^*^,^*^d^
C881	phenyl	COOH	3-COOH	>100^c^*^,^*^e^
C882	phenyl	COOH	4-COOH	>100^c^*^,^*^e^
C883	4-methoxyphenyl	NO_2_	3-COOH	>100^c^*^,^*^e^
C884	4-methoxyphenyl	NO_2_	4-COOH	>100^c^*^,^*^e^
C885	4-methoxyphenyl	COOH	3-COOH and 4-OH	30–40^c^*^,^*^e^
C886	4-methoxyphenyl	COOH	3-COOH	>100^c^*^,^*^e^
C887	4-methoxyphenyl	COOH	4-COOH	>100^c^*^,^*^e^
C888	4-methoxyphenyl	COOH	3-(1*H*-tetrazol-5-yl)	>100^c^*^,^*^e^
C891	4-bromophenyl	NO_2_	3-COOH	>100^c^*^,^*^e^
C892	4-bromophenyl	NO_2_	3-(1*H*-tetrazol-5-yl)	>100^c^*^,^*^e^
C893	4-bromophenyl	COOH	3-COOH and 4-OH	30–40^c^*^,^*^e^
C894	4-bromophenyl	COOH	3-COOH	>100^c^*^,^*^e^
C895	4-bromophenyl	COOH	4-COOH	>100^c^*^,^*^e^
C896	4-bromophenyl	COOH	3-(1*H*-tetrazol-5-yl)	>100^c^*^,^*^e^
C897	phenyl	thiazol-2-yl	3-COOH	30–40^c^*^,^*^e^
C898	phenyl	thiazol-2-yl	3-(1*H*-tetrazol-5-yl)	>100^c^*^,^*^e^
C899	4-bromophenyl	thiazol-2-yl	4-COOH	>100^c^*^,^*^e^
C900	4-methoxyphenyl	thiazol-2-yl	3-COOH	>100^c^^,^^e^
C901	4-methoxyphenyl	thiazol-2-yl	3-(1*H*-tetrazol-5-yl)	>100^c^*^,^*^e^
C902/PH-31	4-bromophenyl	thiazol-2-yl	3-COOH and 4-OH	5^b^*^,^*^d^
C903	4-bromophenyl	thiazol-2-yl	3-COOH	>100^c^*^,^*^e^
PH-30	4-bromophenyl	thiazol-2-yl	3-OH and 4-COOH	>100^b^*^,^*^e^
PH-35	4-bromophenyl	thiazol-2-yl	4-OH	>100^b^*^,^*^e^
PH-36	phenyl	COOH	4-OH	>100^b^*^,^*^e^
PH-37	phenyl	thiazol-2-yl	3-NO_2_ and 4-OH	41^b^
PH-38	phenyl	COOH	3-OH and 4-COOH	16^b^
PH-43	furan-2-yl	thiazol-2-yl	3-COOH and 4-OH	5^b^
PH-44	3,4-dimethoxyphenyl	thiazol-2-yl	3-COOH and 4-OH	12^b^

a Tested in quadruplicate.

b Starting from a maximum concentration of 60 μM, eight concentrations in total.

c Starting from 30 μM, eight concentrations in total.

d Data of in-house synthesized compound, PH-series.

e Extrapolated based on the observed IC_50_ curves.

This series of pyrrolinones and pyrazoles were previously reported as stabilizers of the protein–protein interaction (PPI) involving the plant 14-3-3 proteins,^33,36,37^ which are small adapter proteins that interact with functionally diverse protein partners to play important regulatory roles in vital cellular events including signal transduction, cell cycle control, and apoptosis.^38^ An analysis combining the reported PPI stabilizing activity between 14-3-3 and the plant plasma membrane H^+^-ATPase 2 (PMA2) and PRI inhibitory activity against LIN28–*let-7* revealed in this study showed intriguing SAR information surrounding the pyrrolinones and pyrazoles (Figure S7). The pyrrolinones C902, C880 and the furan analogue PH-43 that showed the most potent LIN28–*let-7* inhibitory activities in our study were compared with compounds PPI-1, C904, and PPI-2 that showed the most potent 14-3-3–PMA2 stabilizing activities.^33^ A general trend is that pyrrolinones that have shown micromolar inhibitory activity against LIN28–*let-7* interaction tended to have minimal stabilizing effects toward 14-3-3–PMA2, all with less than 30% of the stabilizing activity of that of compound PPI-1, which itself showed a weak stabilizing potency (EC_50_: ∼100 μM). In contrast, pyrazole is a favored scaffold for the stabilization of 14-3-3–PMA2 interaction owing to the rigidity of the pyrazole scaffold that enables deeper binding to the PPI interface and enlarged contact surface,^33^ whereas all the tested pyrazoles C904–908 were inactive against LIN28–*let-7* interaction (Table S1).

The salicylic acid moiety, *N*-3-carboxy-4-hydroxyphenyl substituent, is important for modulating PPI and PRI and can be tolerated with a few modifications in either case. The 5-phenyl of the pyrrolinones, equivalent to the 4-phenyl of the pyrazoles, plays a crucial role in determining whether the resulting pyrrolinone is a PRI inhibitor or a PPI activator, as in the cases C880 and compound PPI-1, whose only difference is either a 4-carboxy or a 4-nitro group at the 5-phenyl substituent on the pyrrolinone core. Consistently, the pyrazoles C904 and PPI-2 that showed the most potent 14-3-3 PPI stabilizing activity also bear the signature nitro group at the equivalent position. Additionally, both a 4-carboxy and a 4-(thiazol-2-yl) on the 4-phenyl moiety of pyrrolinones are shown as being the preferred moieties contributing to LIN28–*let-7* inhibitory activity. In summary, the fact that a certain substitution pattern is only leading to potency in one direction, either LIN28–*let-7* inhibition or 14-3-3–PMA2 stabilization, which is “switchable” using another set of substituents, is a valuable feature for this series of trisubstituted pyrrolinones and pyrazoles that needs to be studied further.

In this study, we identified the trisubstituted pyrroliones as a new class of small-molecule inhibitors targeting LIN28–*let-7* interaction. The most potent compounds C902 and PH-43 showed low micromolar inhibitory potency. The structural binding analysis showed potential key molecular interactions between C902 and LIN28A. SAR analysis combining the PRI inhibitory activity in this study and reported PPI stabilizing activity of compounds with the same pyrrolinone and closely related pyrazole scaffolds revealed the pharmacophores contributing to either LIN28–*let-7* inhibition or 14-3-3–PMA2 stabilization. To the best of our knowledge, this is the first report of a dual PRI and PPI modulating scaffold, whose PRI inhibitory and PPI stabilizing activities can be tuned by carefully choosing different substituents attached to its core. Therefore, the identified trisubstituted pyrrolinones not only represent a new LIN28-binding scaffold that diversifies the limited collection of available LIN28 inhibitors but, more significantly, stand as a new class of compounds with a switchable substituent-dependent mechanism of modulation targeting the challenging PRI and PPI.

## Supporting Information Available

Experimental procedures, supplementary biochemical and biophysical data, synthetic procedures, compound characterization data, ^1^H NMR and ^13^C NMR spectra of in-house synthesized compounds (PDF)

## Abbreviations

CSD: cold shock domain
EMSA: electrophoretic mobility shift assay
FP: fluorescence polarization
PPI: protein–protein interaction
preE: precursor element
PRI: protein–RNA interaction
SAR: structure–activity relationship
ZKD: zinc knuckle domain.
