# Supplementary material for: Trisubstituted Pyrrolinones as Small-Molecule Inhibitors Disrupting the Protein–RNA Interaction of LIN28 and Let-7
Source: ACS Med Chem Lett. 2021 Mar 1;12(6):893–8. doi: 10.1021/acsmedchemlett.0c00546 (PMC8201479; doi:10.1021/acsmedchemlett.0c00546)
Supplement: Supplementary file 1 — ml0c00546_si_001.pdf [file ml0c00546_si_001.pdf]

Supporting Information

Trisubstituted Pyrrolinones as Small-Molecule Inhibitors Disrupting the Protein–RNA Interaction of LIN28 and *Let-7*

Lydia Borgelt,[†,‡,∥] Fu Li,[†,‡,∥] Pascal Hommen,[†,‡,∥] Philipp Lampe,[‡,§,∥] Jimin Hwang,[†,‡] Georg L. Goebel,[†,‡] Sonja Sievers,[‡,§] and Peng Wu[†,‡,]*

[†]Chemical Genomics Centre, Max Planck Institute of Molecular Physiology, Dortmund 44227, Germany

[‡]Department of Chemical Biology, Max Planck Institute of Molecular Physiology, Dortmund 44227, Germany

[§]Compound Management and Screening Center, Dortmund 44227, Germany

[∥]Equally contributed authors

*E-mail: peng.wu@mpi-dortmund.mpg.de

CONTENTS

EXPERIMENTAL PROCEDURES ………………….……….……………………….......... Page S3

SUPPLEMENTAL BIOCHEMICAL AND BIOPHYSICAL DATA ……………..………... Page S7

GENERAL CHEMISTRY INFORMATION ……………………………….……..……….. Page S25

SYNTHETIC PROCEDURES AND COMPOUND CHARACTERIZATION …..……….. Page S27

ANALYTICAL UHPLC AND NMR SPECTRA …………..…………………...………….. Page S35

REFERENCES …………..…………………………………….………….....……...….. Page S67

EXPERIMENTAL PROCEDURES

Protein expression and purification. Human LIN28A (residues 16–187) was subcloned into pET-19 vector and overexpressed in *Escherichia coli BL21(DE3).* The culture was incubated at 37 °C until the absorbance at 600 nm (OD600) reached 0.5–0.7. IPTG was added to a final concentration of 300 µM, and overnight induction was performed at 18 °C. Cells were harvested by centrifugation. The bacterial pellet was resuspended in lysis buffer (50 mM NaH_2PO_4, pH 7.5, 300 mM NaCl, 0.1 mM PMSF) and lysed using a Microfluidizer (Microfluidics). A fresh portion of 0.1 mM PMSF and Triton X-100 (1% final concentration) were added. The lysate was cleared by ultracentrifugation at 30000 xg and 4 °C for 1 h. The protein purification was performed using immobilized nickel affinity chromatography (HisTrap, GE Healthcare) in a buffer containing 50 mM NaH_2PO_4 (pH 8), 300 mM NaCl and 5 % glycerol. Gradient elution was performed with a maximum of 0.5 M imidazole. Subsequently, the affinity tag was cleaved using His6-TEV-protease and the protease and unspecific binders were eliminated by a second nickel affinity chromatography. After protein concentration LIN28A containing fractions were concentrated and applied to a High Load Superdex 75 pg 16/600 column (GE Healthcare) with gel-filtration buffer (30 mM NaH_2PO_4, pH 7.5, 50 mM NaCl, 5% glycerol, 2 mM β-ME). The purified protein was concentrated and stored at -80 °C until further applications.

Fluorescence polarization. The fluorescence polarization assay was performed in black low volume polystyrene 384 well plates (Corning 4514). IC_{50} measurements were performed with three technical replicates. Purified LIN28A (residues 16–187) was incubated at a concentration of 200 nM for 30 minutes with compound concentrations ranging from 0.3 nM to 100 µM in FP assay buffer (20 mM Tris, pH 7, 100 mM NaCl, 5 mM $MgCl_2$, 2 mM glutathione (reduced), 0.1 % NP-40). Subsequently, FAM-labeled preE-*let-7f-1* (mus musculus) (GGGGUAGUGAUUUUACCCUGUUUAGGAGAU-FAM, synthesized by IDT) was added to a final concentration of 2 nM. Fluorescence polarization

was detected after incubation at room temperature using a TECAN Spark plate reader. Half maximal inhibitory concentrations (IC_{50}) were determined using GraphPad Prism 7.

Compound screening. High throughput screening was performed in low volume 384 well plates (Corning 4514) with 30 µM compound, 40 nM LIN28A, and 2 nM *preE-let-7f-1*-FAM in FP assay buffer. Compounds were transferred with an ECHO 520 liquid handler and protein and RNA were added with a multidrop dispenser. Protein and RNA were added stepwise with a 30 min incubation period in between. FP was detected after a final incubation of 15 min with an Envision plate reader and fluorescence was measured with a Paradigm plate reader. A reaction without LIN28 served as a negative control and one without compound was used as a positive control. The software Quattro Workflow (Quattro Research GmbH) was used for the analysis of screening data.

Electrophoretic mobility shift assay. EMSA was used as a secondary assay to validate FP-assay hit molecules. Purified LIN28A (residues 16–187) was incubated with compound and 5 U recombinant ribonuclease inhibitor (Takara Bio) in EMSA reaction buffer (50 mM Tris (pH 7.5), 100 mM NaCl, 10 mM β-mercaptoethanol, 50 µM $ZnCl_2$, 2 % DMSO, 0.01 % Tween 20, 12 % glycerol) for 2 hours at room temperature. Subsequently, preE-*let-7f-1*-Cy3 (*mus musculus*) (GGGGUAGUGAUUUUAC CCUGUUUAGGAGAU-Cy3, purchased from IDT) was added to a final concentration of 5 nM and a reaction volume of 50 µL. The final concentration of LIN28A was 10 nM and compound concentrations up to 75 µM were used. The reaction mixtures were incubated for a further 15 minutes and 10 µL loading dye (40 % glycerol, 1.5x TAE) was added. 10 µL of each reaction was separated in an 8 % polyacrylamide TAE gel at 4 °C and 220 V for 1 h using 0.25x TAE as running buffer. Cy3 fluorescence was detected with a ChemiDoc MP (Bio-Rad) and 2 minutes of exposure time.

Thermal shift assay. Thermal stability of the cold shock domain construct LIN28A (residues 16–126) with and without compound was monitored using a NanoTemper Prometheus NT.48 nano-

differential scanning fluorimetry (nanoDSF) instrument at temperatures between 20 °C and 90 °C and a temperature slope of 1 °C per minute with an excitation power of 50–60 %. A total protein concentration of 30 µM and compound concentrations from 100 µM to 0.78 µM corresponding to 5 % to 0.039 % DMSO, respectively, were used. Compound and protein were incubated for 45 minutes at room temperature in buffer containing 30 mM NaH_2PO_4, 50 mM NaCl, 1 mM $MgCl_2$, and 50 µM $ZnCl_2$ at pH 8 and 10 µL sample was loaded to each nanoDSF standard capillary (NanoTemper). The ratio of intrinsic tryptophan fluorescence at 350 nm and 330 nm was measured and the first derivative was determined to detect the inflection points using the software of the device.

RT-qPCR. JAR cells (obtained from DSMZ, German Collection of Microorganisms and Cell Cultures, Braunschweig, Germany, DSMZ no. ACC462)[1] were cultured in 24-well plates and treated with the compound (PH-31) for 24 h with a total DMSO concentration of 0.5 % in all wells. RNA was extracted using RNeasy Mini Kit (Qiagen) and 20 ng of total RNA was used for reverse transcription using the TaqMan microRNA Reverse Transcription Kit (Applied Biosystems) following the manufacturer's protocols. We then performed qPCR using TaqMan Universal Master Mix II, with UNG (Applied Biosystems) according to the manufacturer's protocol using a CFX Connect Real-Time PCR System (BioRad). TaqMan microRNA assays were purchased from Applied Biosystems (Assay IDs: 001093, 000377, 002282). Relative expression levels were normalized to U6 snRNA and samples treated with DMSO using the reported $2^{-\Delta\Delta CT}$ method.[2]

Docking analysis. Computational docking of pyrrolinones to preE-*let-7* binding site of LIN28 CSD (PDB code: 5UDZ) was done by Schrödinger Maestro 12.3. The 3-dimensional structures of pyrrolinones were prepared after calculating energy minimization by mm2 at Chem3D 18.2 and the chemical states were generated by the ligand preparation module. The conformation of LIN28 was prepared according to the protein preparation module, including hydrogen addition, water molecule removal, and energy minimization, after removing preE-*let-7* fragments except for oligomers used as

ligand. The binding site was generated by grid generation module and using preE-*let-7* fragment (A6-U8, U8-U11, or U13-A15) as the ligand. The glide dock was performed by glide dock module and evaluated according to the docking score, small molecule orientation, solvent exposure, and interactions between small molecules and LIN28. The interactions were visualized by using PyMOL.

SUPPLEMENTAL BIOCHEMICAL AND BIOPHYSICAL DATA

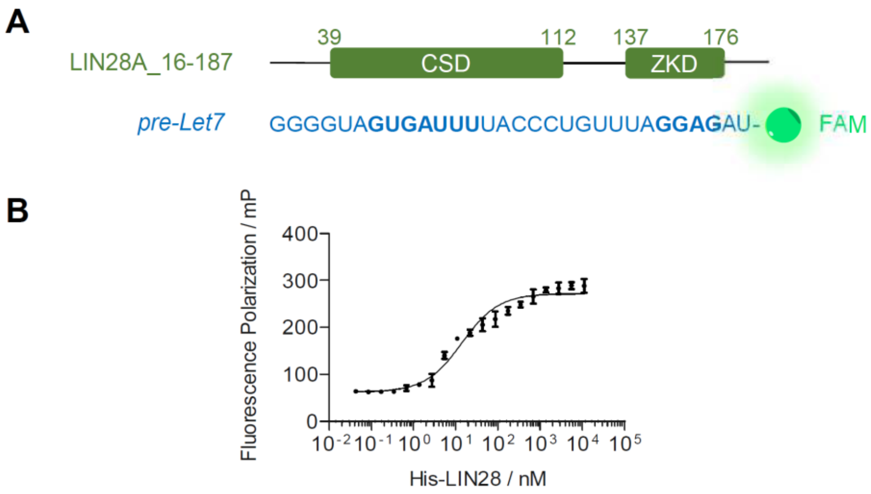

Figure S1. (A) A truncated human LIN28A (residues 16–187) containing both the CSD and ZKD domains and a FAM-labeled preE-*let-7f-1* miRNA were used in the fluorescence-polarization (FP) assay to identify inhibitors disrupting the LIN28–*let-7* interaction. (B) His-LIN28A (residues 16–187) titrated to 2 nM FAM-labeled preE-*let-7f-1* miRNA, three replicates, error bars indicate ±SD. His-LIN28A-bound preE-*let-7f-1* leads to increased FP (mP).

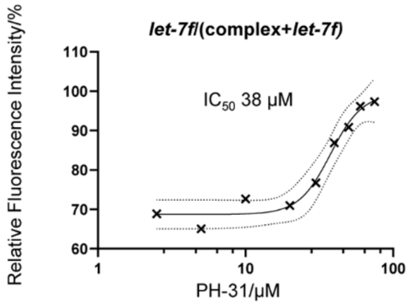

Figure S2. The measured IC_{50} values of PH-31 in EMSA. The IC_{50} value of 38 μM was calculated by the relative fluorescence intensity of *let-7f* divided by that of the LIN28–*let-7f* complex plus *let-7f*.

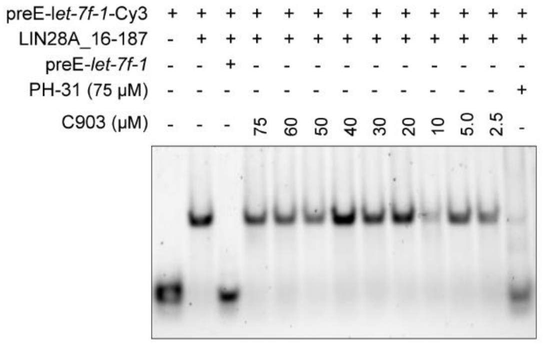

Figure S3. The inactive analogue C903 did not show inhibitory activity against the LIN28–*let-7f* interaction in EMSA.

A

Capillary	Sample (PH-031)	Inflection point
1	No compound	38.67 ± 0.12
2	50 μM	40.25 ± 0.65
3	25 μM	40.07 ± 0.52
4	12.5 μM	39.83 ± 0.39
5	6.25 μM	39.53 ± 0.34
6	3.125 μM	39.23 ± 0.29
7	1.563 μM	39.03 ± 0.17
8	0.781 μM	38.97 ± 0.22

B

Capillary	Sample (PH-036)	Inflection point
1	No compound	38.67 ± 0.12
2	50 μM	39.23 ± 0.09
3	25 μM	39.03 ± 0.05
4	12.5 μM	38.90 ± 0.08
5	6.25 μM	38.73 ± 0.09
6	3.125 μM	38.70 ± 0.00
7	1.563 μM	38.63 ± 0.05
8	0.781 μM	38.60 ± 0.08

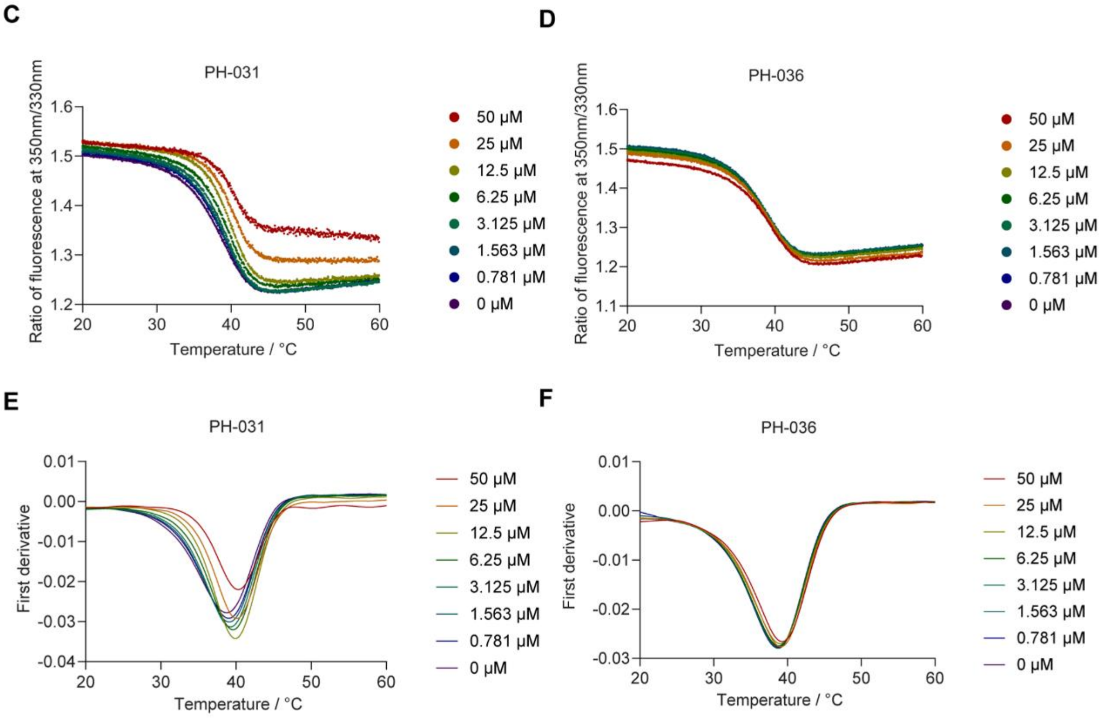

Figure S4. Thermal shift assay from nano-differential scanning fluorimetry (DSF). (A) PH-31 led to the concentration-dependent enhancement of thermal stability of the CSD of LIN28A (residues 16-126) using nano-DSF. (B) The data for the inactive compound PH-36. (C) The ratio of fluorescence at 350 nM/330 nM for PH-31. (D) The ratio of fluorescence at 350 nM/330 nM for PH-36. (E) First derivative graph for PH-31. (F) First derivative graph for PH-36.

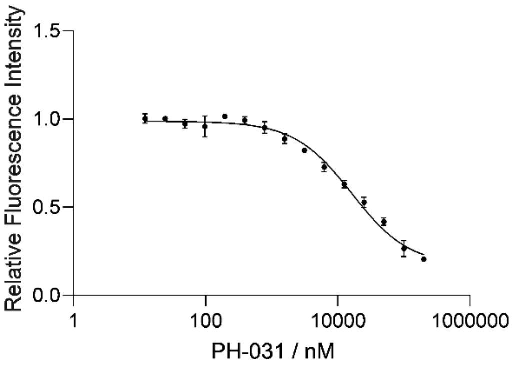

Figure S5. Fluorescence quenching assay measuring binding of PH-31 to the cold-shock domain of LIN28A (Kd: 16.5 ± 2.8 µM).

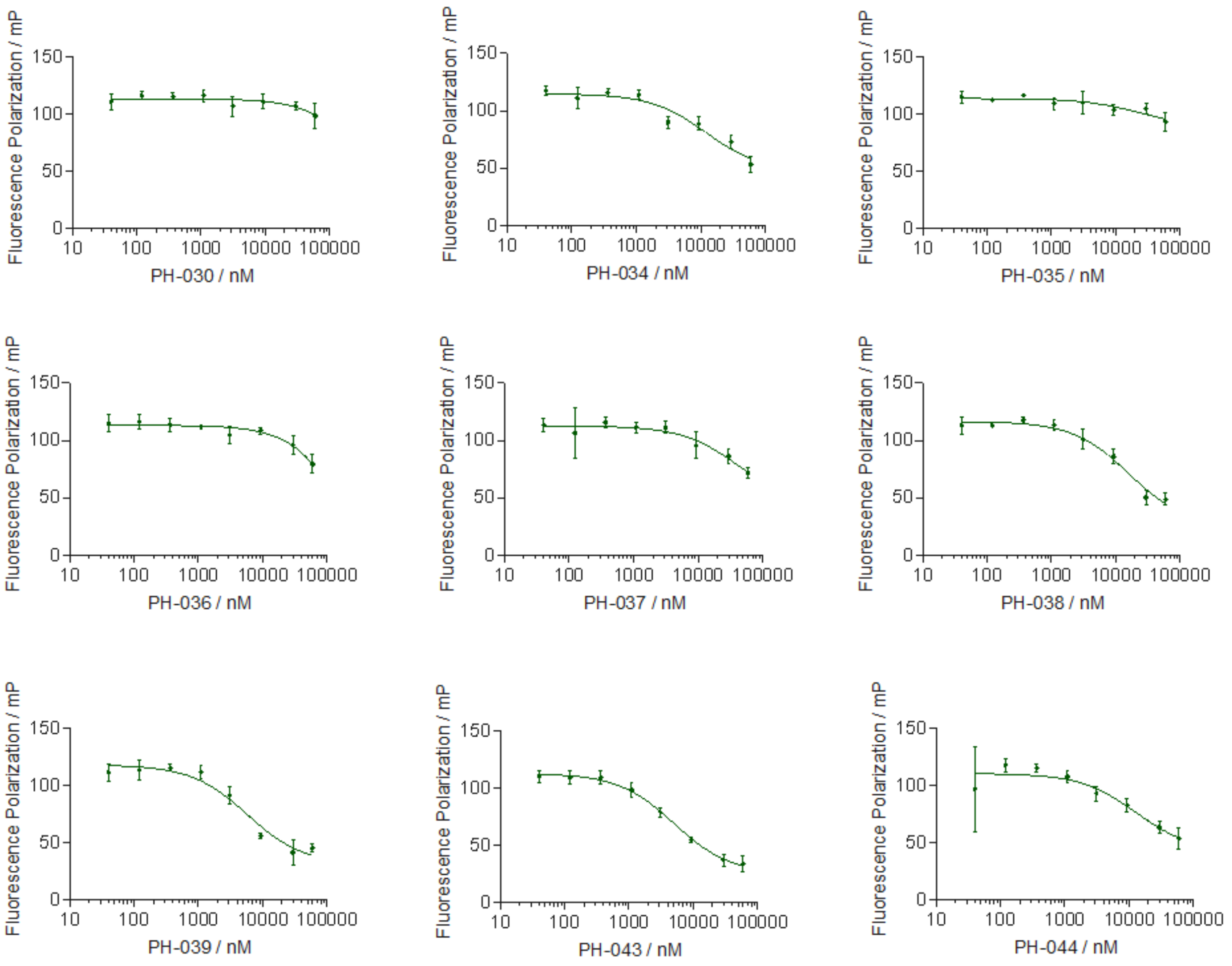

Figure S6. Dose-dependent inhibition of the in-house synthesized compounds (PH series) in the FP assay. The figure for PH-31 has been included as Figure 3D in the main manuscript, and the IC_{50} values are summarized in Table 1 of the main manuscript.

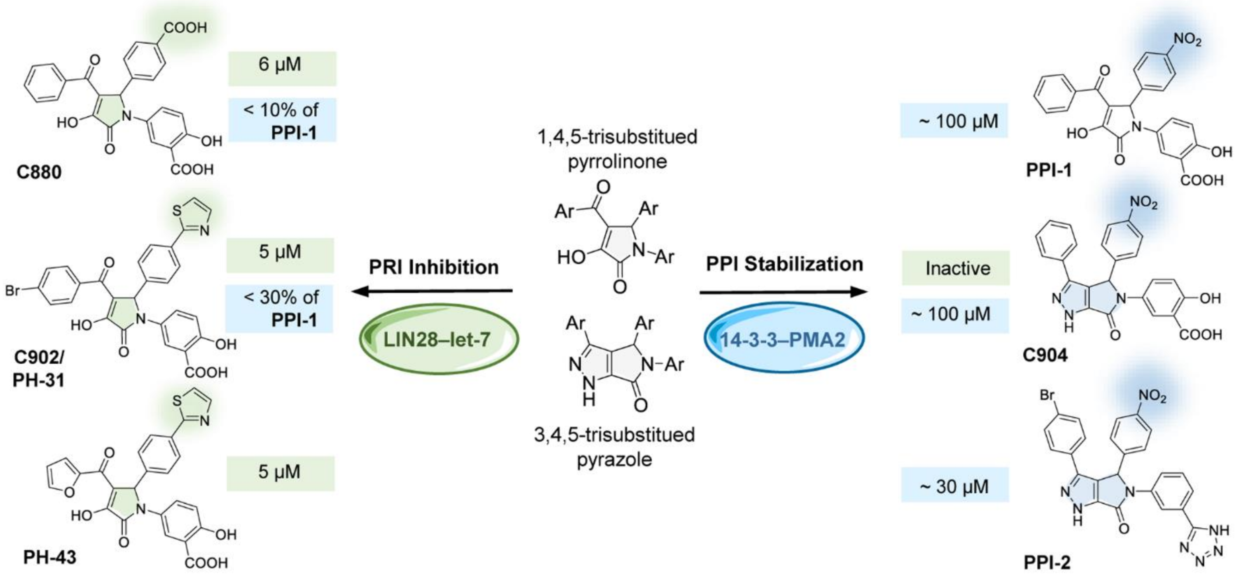

Figure S7. Comparison of the LIN28–*let-7* inhibitory activity and 14-3-3–PMA2 stabilizing activity of representative 1,4,5-trisubstituted pyrrolinones and 3,4,5-trisubstituted pyrazoles. Pyrrolinones showed micromolar LIN28–*let-7* inhibitory activity and minimal 14-3-3–PMA2 stabilizing activity, while pyrazoles are inactive against LIN28–*let-7* but are favored scaffolds for 14-3-3–PMA2 stabilization. The IC_{50} against LIN28–*let-7f-1* are shown in green rectangles and the EC_{50} towards the 14-3-3 protein T14-3e and the PMA2-CT52YDI protein are shown in blue rectangles (including stabilizing effect in % towards T14-3c–CT66YDI protein at a compound concentration of 100 µM).[3] PPI, protein–protein interaction; PRI, protein–RNA interaction.

Table S1. LIN28–*let-7* inhibitory activity of trisubstituted pyrazoles

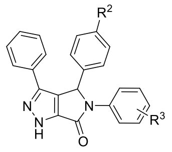

Compound ID	R^2	R^3	IC_{50} (μM)$^{a-c}$
C904	NO_2	3-COOH and 4-OH	>100
C905	NO_2	4-COOH	>100
C906	NO_2	4-methoxyphenylcarbamoyl	>100
C907	Br	3-COOH and 4-OH	>100
C908	NO_2	4-benzylcarbamoyl	>100

[a]Tested in quadruplicates; [b]Starting from a maximum concentration of 30 μM, 8 concentrations in total; [c]Extrapolated based on the observed IC_{50} curves.

Table S2. Dose-Average Z’ factor values for the LIN28 fluorescence polarization assay (for compounds in Table 1 and Table S1)

Methods	Method description	Compounds tested in this method	Z’ factor (average of quadruplicates)
A	Starting from a max. concentration of 30 µM, 8 concentrations in total	C881–888, C891–C901, C903–C908	0.81
B	Starting from a max. concentration of 60 µM, 8 concentrations in total	PH-30, PH-31, PH-34–PH-39, PH-43, PH-44	0.71

Table S3. (Part I) Raw data for the ten PH compounds in the LIN28 fluorescence polarization assay in Table 1

**PH-30**						
Concentration (μM)	Replicate 1 (mP)	Replicate 2 (mP)	Replicate 3 (mP)	Replicate 4 (mP)	Average (mP)	Standard deviation
59.375	89.00	110.00	89.70	106.00	98.68	10.89
29.688	106.00	111.00	102.00	110.00	107.25	4.11
9.375	108.00	115.00	104.00	118.00	111.25	6.40
3.125	99.50	113.00	99.50	116.00	107.00	8.75
1.103	111.00	122.00	119.00	113.00	116.25	5.12
0.368	116.00	117.00	112.00	118.00	115.75	2.63
0.123	111.00	119.00	117.00	118.00	116.25	3.59
0.041	116.00	113.00	101.00	113.00	110.75	6.65
**PH-31**						
Concentration (μM)	Replicate 1 (mP)	Replicate 2 (mP)	Replicate 3 (mP)	Replicate 4 (mP)	Average (mP)	Standard deviation
59.375	44.40	37.50	38.70	34.40	38.75	4.18
29.688	41.00	44.90	43.60	42.70	43.05	1.64
9.375	64.70	70.50	55.50	65.40	64.03	6.24
3.125	74.00	79.30	70.30	76.60	75.05	3.84
1.103	115.00	102.00	105.00	101.00	105.75	6.40
0.368	121.00	115.00	107.00	111.00	113.50	5.97
0.123	108.00	116.00	107.00	109.00	110.00	4.08
0.041	108.00	119.00	105.00	105.00	109.25	6.65
**PH-34**						
Concentration (μM)	Replicate 1 (mP)	Replicate 2 (mP)	Replicate 3 (mP)	Replicate 4 (mP)	Average (mP)	Standard deviation
59.375	57.90	56.30	56.70	42.70	53.40	7.17
29.688	77.10	79.60	66.90	68.50	73.03	6.27
9.375	95.10	83.70	92.00	83.70	88.63	5.83
3.125	87.80	83.60	92.10	95.40	89.73	5.13
1.103	113.00	113.00	119.00	109.00	113.50	4.12
0.368	115.00	111.00	119.00	117.00	115.50	3.42
0.123	117.00	115.00	115.00	96.60	110.90	9.58
0.041	120.00	118.00	120.00	111.00	117.25	4.27
**PH-35**						
Concentration (μM)	Replicate 1 (mP)	Replicate 2 (mP)	Replicate 3 (mP)	Replicate 4 (mP)	Average (mP)	Standard deviation
59.375	104.00	95.90	88.50	85.50	93.48	8.27
29.688	109.00	102.00	108.00	99.60	104.65	4.57
9.375	108.00	108.00	99.00	101.00	104.00	4.69
3.125	110.00	123.00	108.00	100.00	110.25	9.54
1.103	112.00	114.00	110.00	102.00	109.50	5.26
0.368	116.00	118.00	116.00	116.00	116.50	1.00
0.123	114.00	112.00	113.00	112.00	112.75	0.96
0.041	114.00	120.00	119.00	108.00	115.25	5.50

Table S3. (Part II)

PH-36						
Concentration (μM)	Replicate 1 (mP)	Replicate 2 (mP)	Replicate 3 (mP)	Replicate 4 (mP)	Average (mP)	Standard deviation
59.375	86.20	81.30	67.60	82.30	79.35	8.11
29.688	99.70	106.00	87.00	91.40	96.03	8.48
9.375	111.00	111.00	105.00	107.00	108.50	3.00
3.125	99.60	97.70	111.00	111.00	104.83	7.17
1.103	114.00	113.00	111.00	110.00	112.00	1.83
0.368	117.00	120.00	107.00	110.00	113.50	6.03
0.123	113.00	126.00	113.00	113.00	116.25	6.50
0.041	115.00	121.00	104.00	120.00	115.00	7.79
**PH-37**						
Concentration (μM)	Replicate 1 (mP)	Replicate 2 (mP)	Replicate 3 (mP)	Replicate 4 (mP)	Average (mP)	Standard deviation
59.375	75.50	64.90	73.20	72.40	71.50	4.59
29.688	80.70	92.50	81.70	91.20	86.53	6.19
9.375	109.00	83.80	88.50	101.00	95.58	11.52
3.125	108.00	119.00	108.00	111.00	111.50	5.20
1.103	115.00	113.00	105.00	112.00	111.25	4.35
0.368	121.00	119.00	113.00	110.00	115.75	5.12
0.123	124.00	117.00	73.90	111.00	106.48	22.36
0.041	108.00	122.00	113.00	110.00	113.25	6.18
**PH-38**						
Concentration (μM)	Replicate 1 (mP)	Replicate 2 (mP)	Replicate 3 (mP)	Replicate 4 (mP)	Average (mP)	Standard deviation
59.375	56.20	48.70	46.20	44.90	49.00	5.05
29.688	53.20	50.80	41.80	55.40	50.30	5.97
9.375	91.60	87.90	77.00	89.60	86.53	6.53
3.125	88.00	107.00	104.00	105.00	101.00	8.76
1.103	119.00	115.00	110.00	109.00	113.25	4.65
0.368	116.00	122.00	116.00	117.00	117.75	2.87
0.123	113.00	114.00	113.00	113.00	113.25	0.50
0.041	106.00	123.00	114.00	109.00	113.00	7.44
**PH-39**						
Concentration (μM)	Replicate 1 (mP)	Replicate 2 (mP)	Replicate 3 (mP)	Replicate 4 (mP)	Average (mP)	Standard deviation
59.375	50.60	46.70	42.40	42.40	45.53	3.94
29.688	56.40	42.50	28.70	38.90	41.63	11.45
9.375	57.10	58.10	54.90	53.80	55.98	1.97
3.125	96.70	94.50	81.20	94.10	91.63	7.04
1.103	108.00	119.00	114.00	107.00	112.00	5.60
0.368	120.00	116.00	114.00	113.00	115.75	3.10
0.123	106.00	126.00	115.00	107.00	113.50	9.26
0.041	107.00	119.00	103.00	117.00	111.50	7.72

Table S3. (Part III)

PH-43						
Concentration (μM)	Replicate 1 (mP)	Replicate 2 (mP)	Replicate 3 (mP)	Replicate 4 (mP)	Average (mP)	Standard deviation
59.375	35.80	42.50	25.90	30.50	33.68	7.14
29.688	34.10	38.20	32.20	44.50	37.25	5.44
9.375	56.00	56.90	51.70	56.10	55.18	2.35
3.125	76.10	80.30	75.10	84.50	79.00	4.30
1.103	105.00	101.00	99.90	89.60	98.88	6.56
0.368	101.00	114.00	113.00	111.00	109.75	5.97
0.123	109.00	111.00	102.00	116.00	109.50	5.80
0.041	113.00	117.00	106.00	106.00	110.50	5.45
**PH-44**						
Concentration (μM)	Replicate 1 (mP)	Replicate 2 (mP)	Replicate 3 (mP)	Replicate 4 (mP)	Average (mP)	Standard deviation
59.375	43.80	62.70	48.30	61.20	54.00	9.38
29.688	57.80	68.40	67.10	60.80	63.53	5.06
9.375	89.30	76.10	79.50	87.00	82.98	6.21
3.125	94.50	101.00	86.90	89.40	92.95	6.23
1.103	115.00	104.00	108.00	104.00	107.75	5.19
0.368	113.00	120.00	113.00	114.00	115.00	3.37
0.123	122.00	123.00	116.00	111.00	118.00	5.60
0.041	111.00	127.00	41.80	107.00	96.70	37.61

Table S4. (Part I) IC_{50} curves for all library compounds in the LIN28 fluorescence polarization (FP) assay in Table 1 and Table S1

Cpd.	IC_{50} curve–FP replicate 1	IC_{50} curve–FP replicate 2	IC_{50} curve–FP replicate 3	IC_{50} curve–FP replicate 4
C905	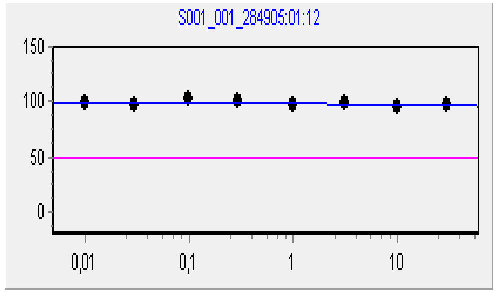	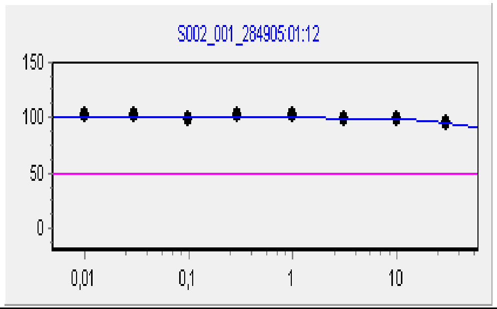	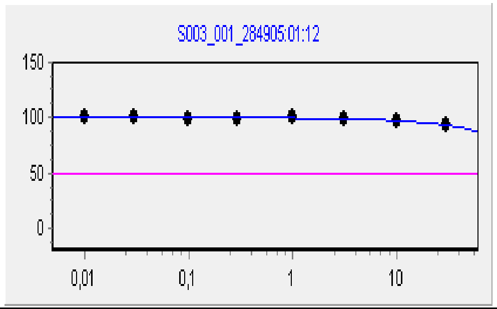	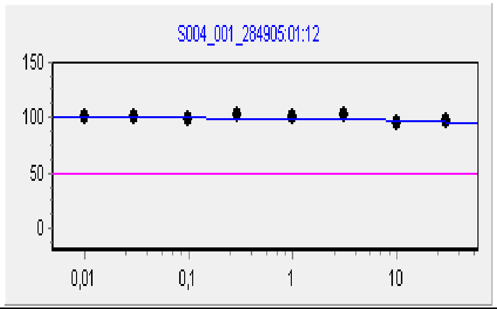
C882	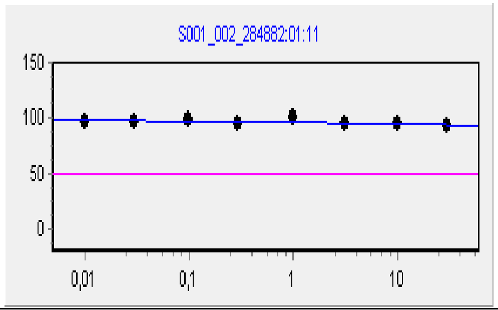	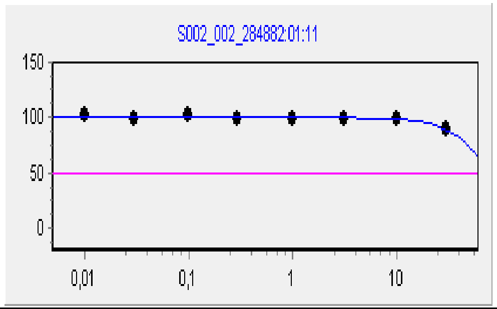	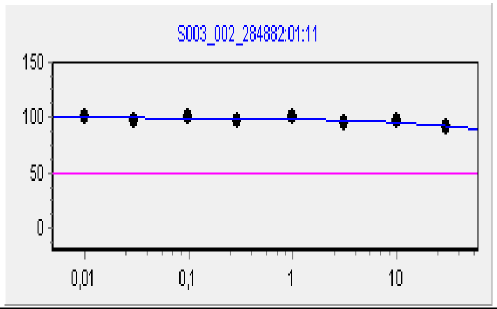	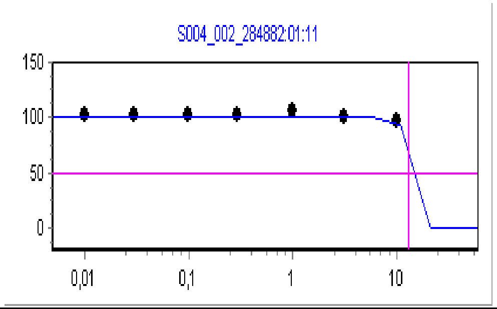
C884	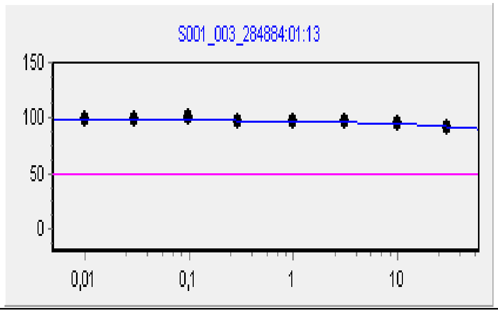	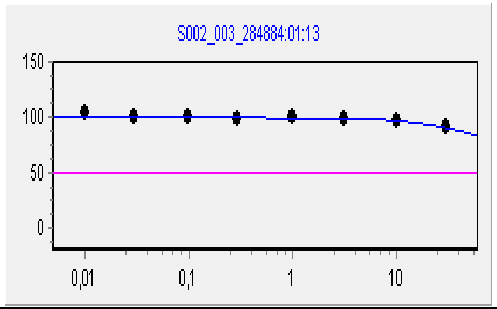	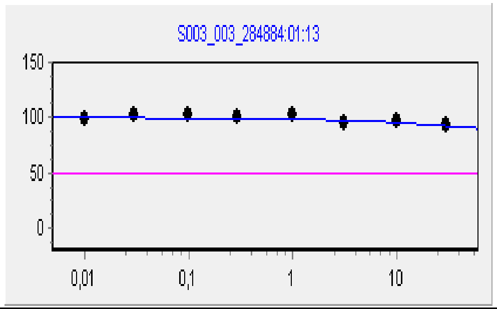	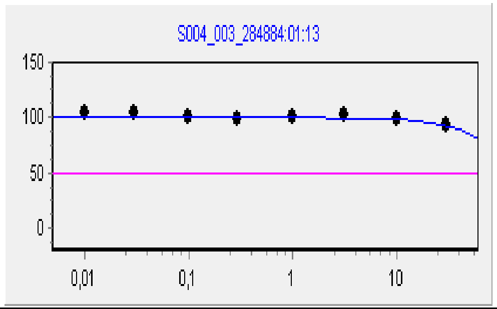
C897	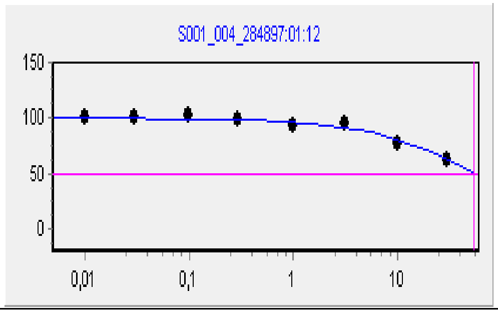	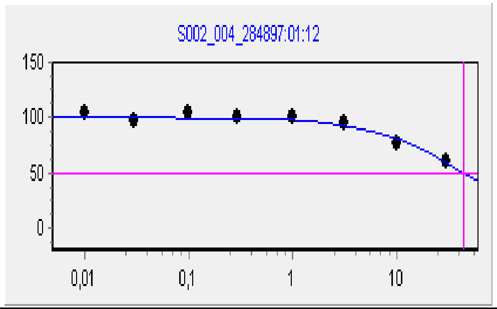	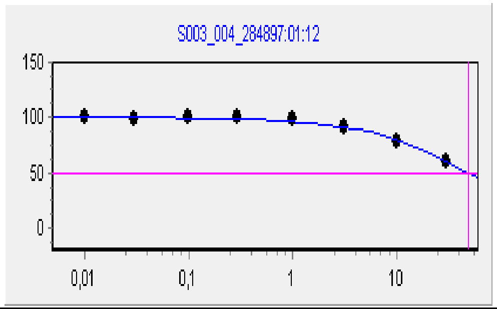	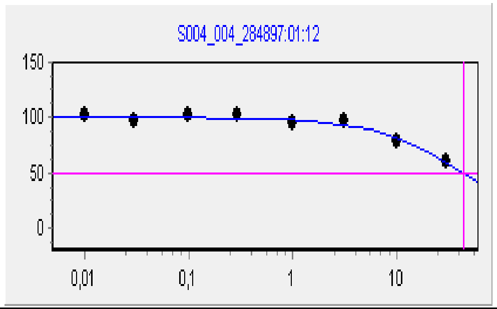

Table S4. (Part II)

Cpd.	IC50 curve–FP replicate 1	IC50 curve–FP replicate 2	IC50 curve–FP replicate 3	IC50 curve–FP replicate 4
C881	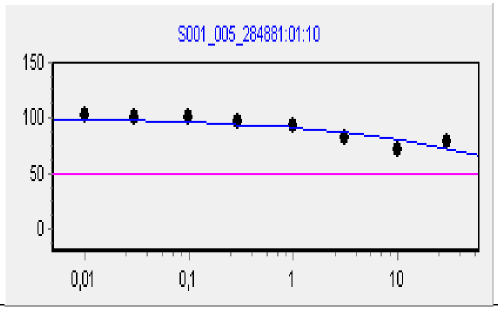	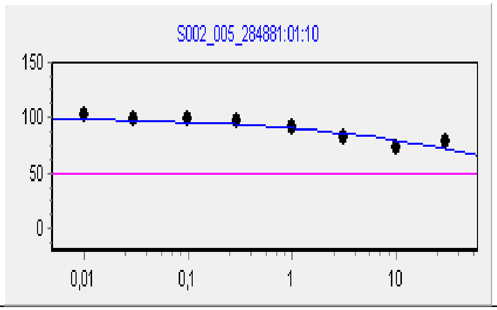	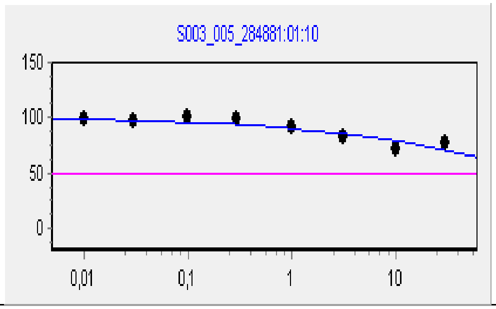	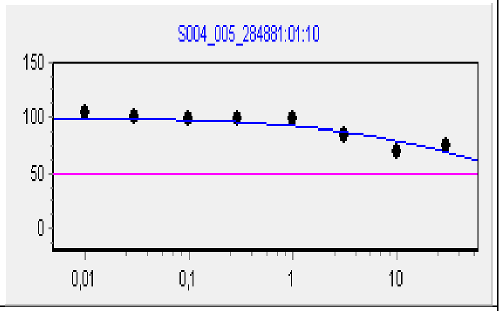
C886	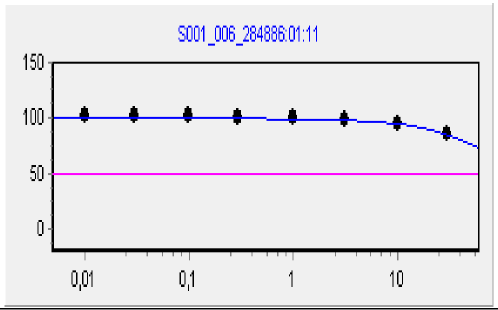	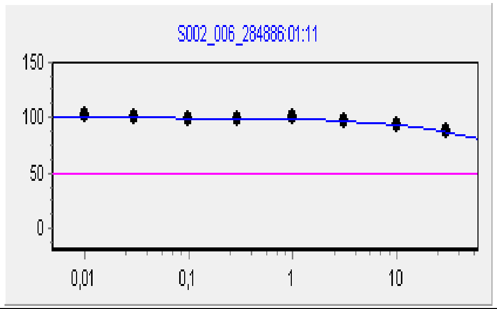	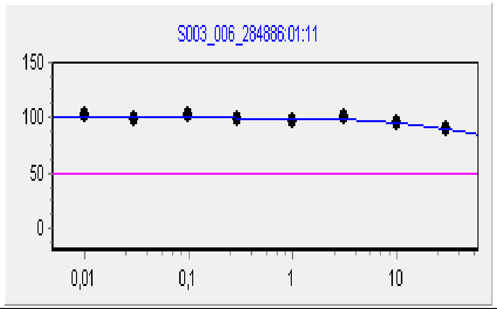	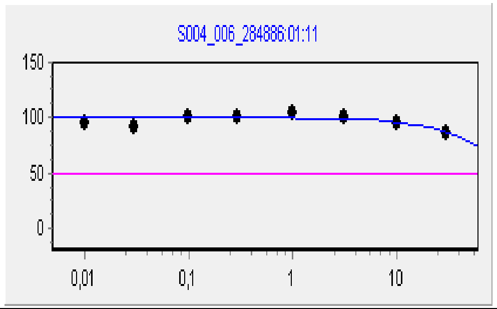
C887	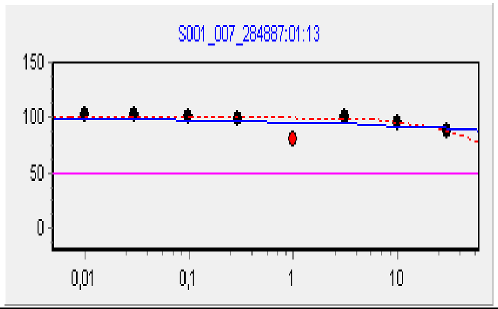	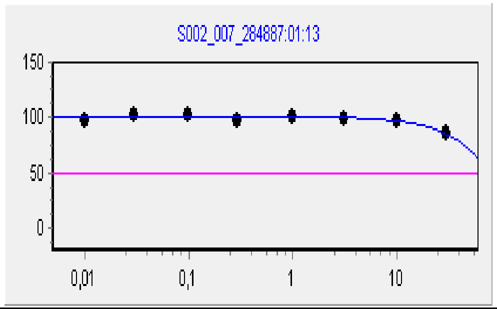	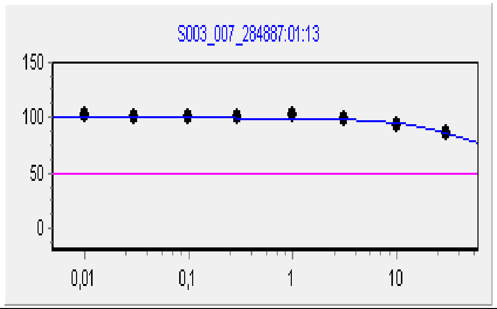	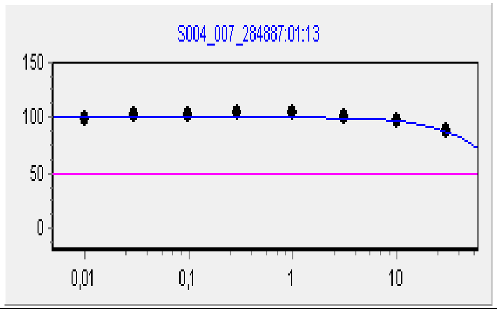
C907	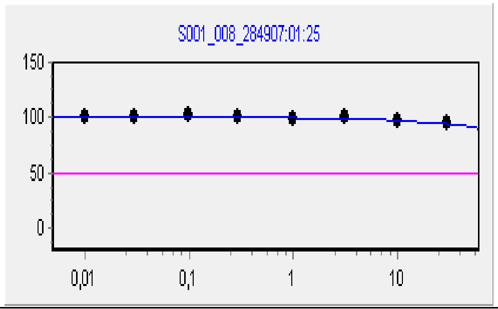	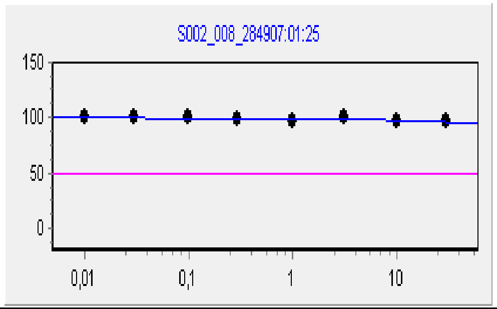	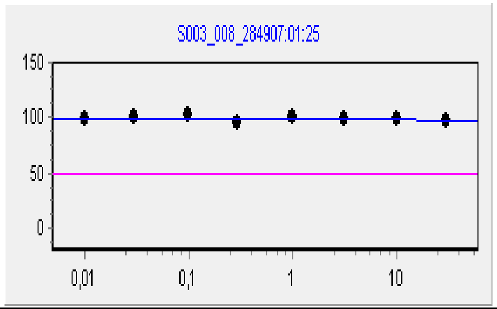	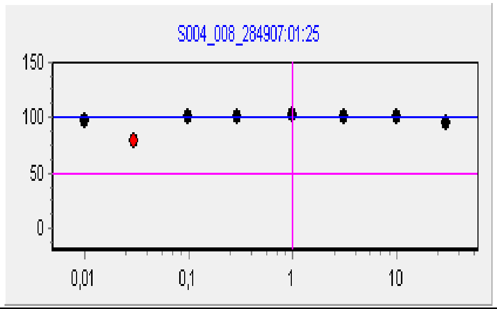

Table S4. (Part III)

Cpd.	IC50 curve–FP replicate 1	IC50 curve–FP replicate 2	IC50 curve–FP replicate 3	IC50 curve–FP replicate 4
C892	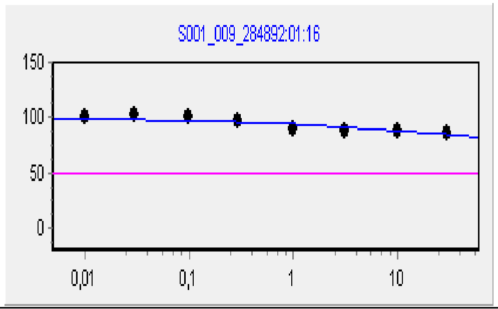	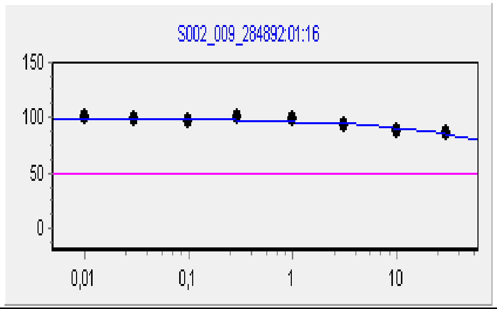	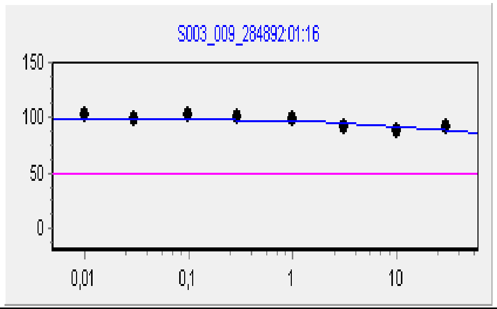	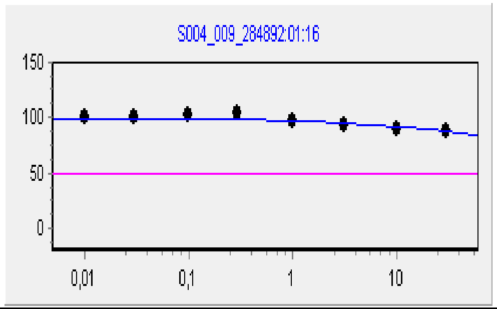
C901	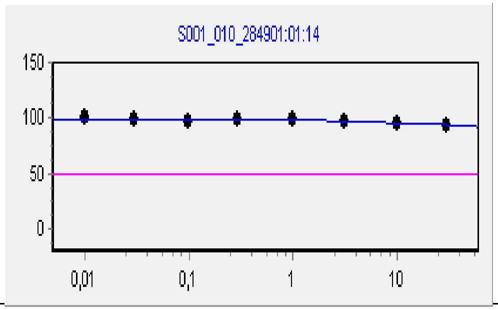	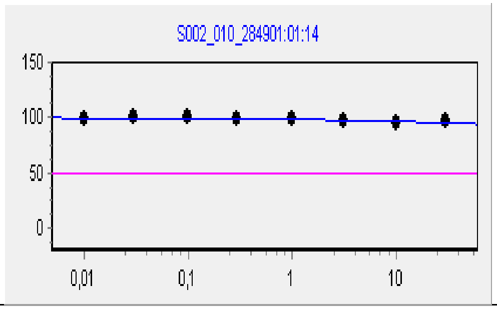	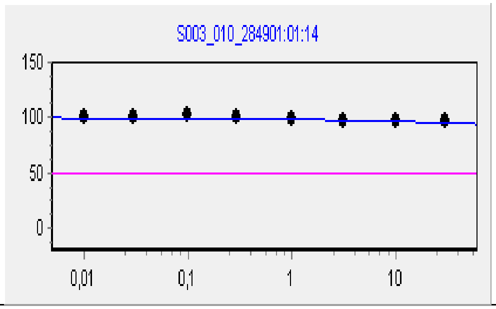	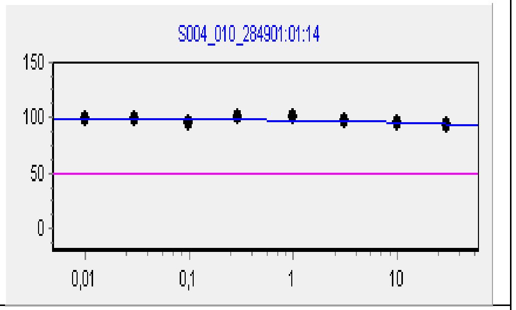
C903	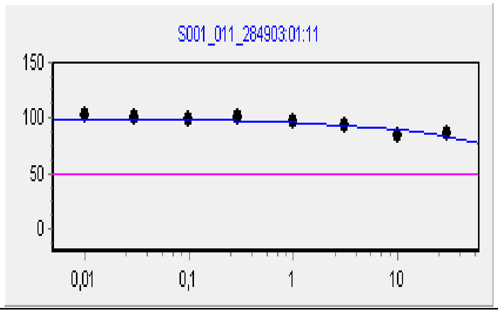	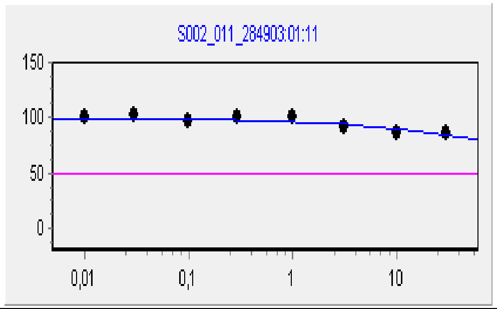	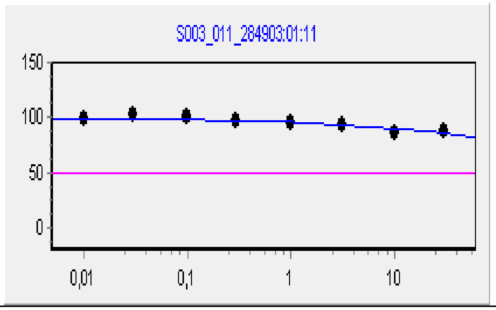	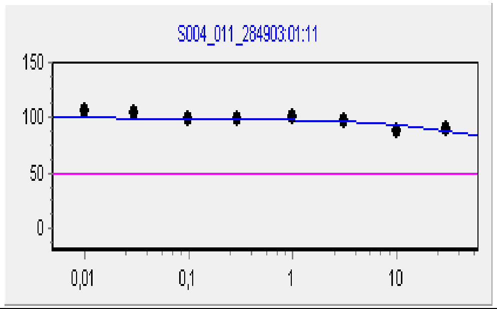
C895	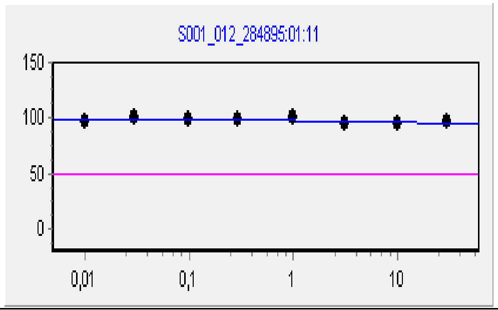	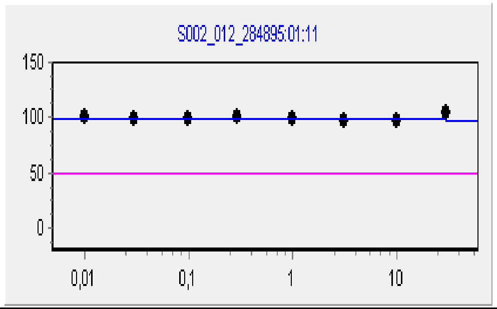	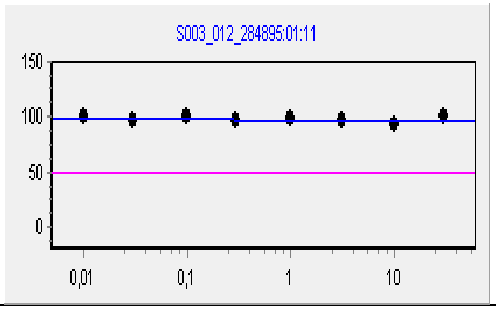	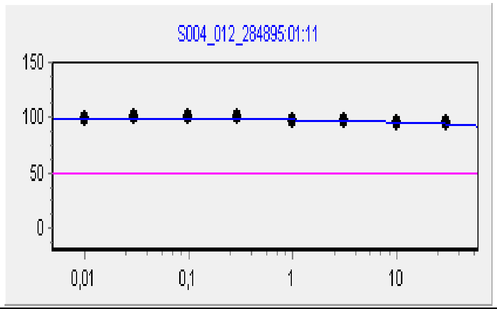

Table S4. (Part IV)

Cpd.	IC$_{50}$ curve–FP replicate 1	IC$_{50}$ curve–FP replicate 2	IC$_{50}$ curve–FP replicate 3	IC$_{50}$ curve–FP replicate 4
C900	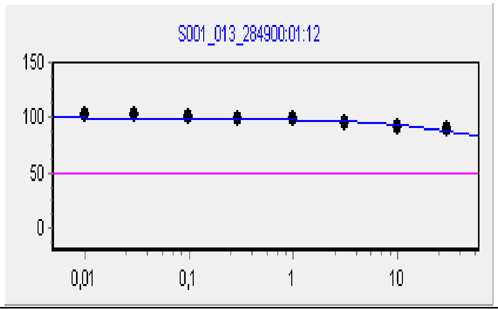	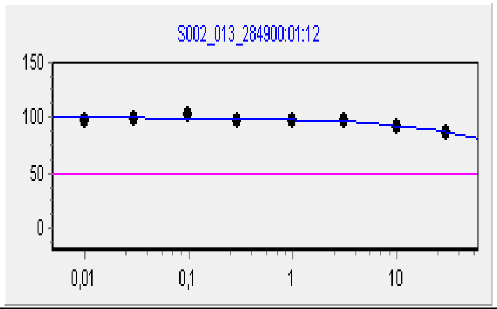	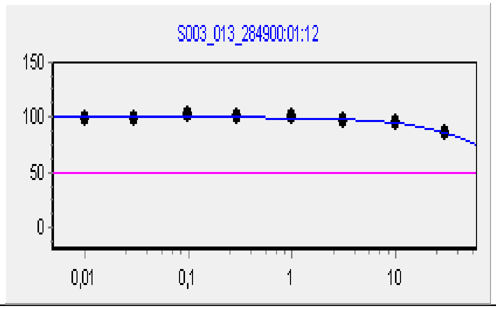	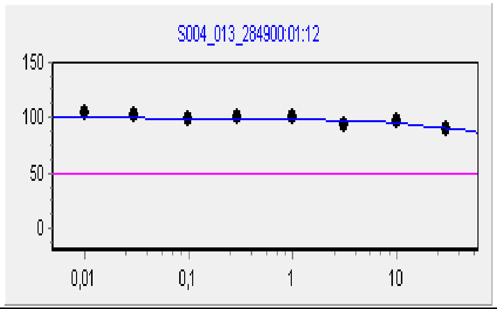
C904	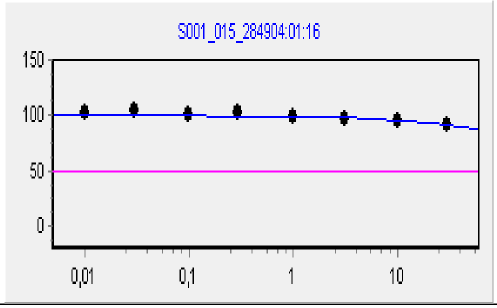	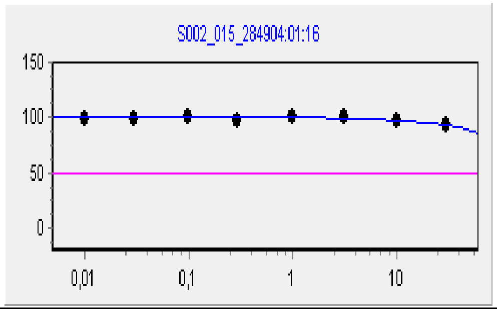	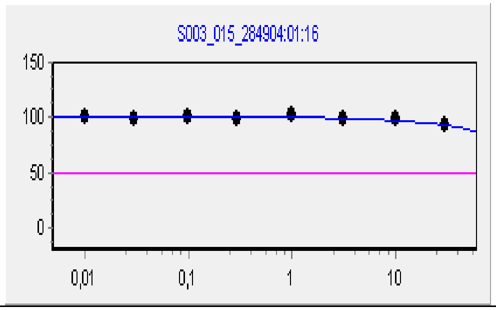	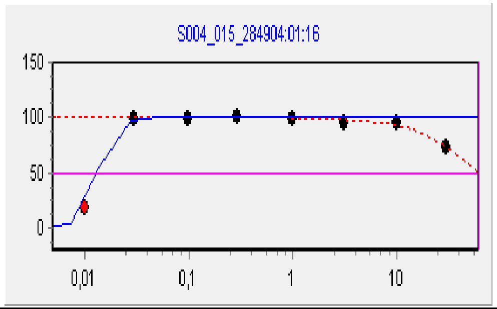
C885	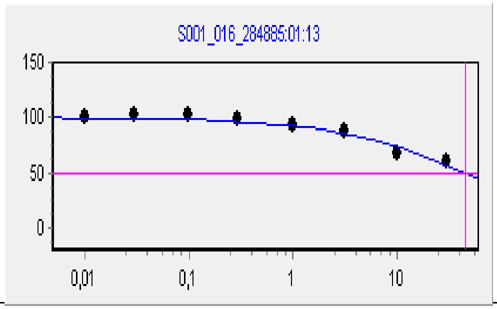	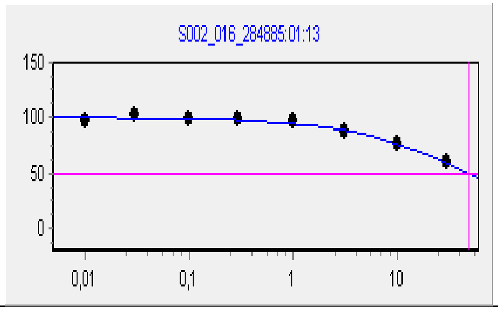	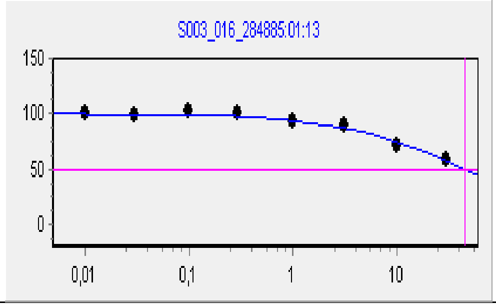	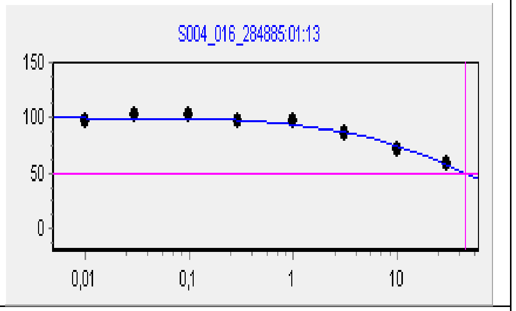
C898	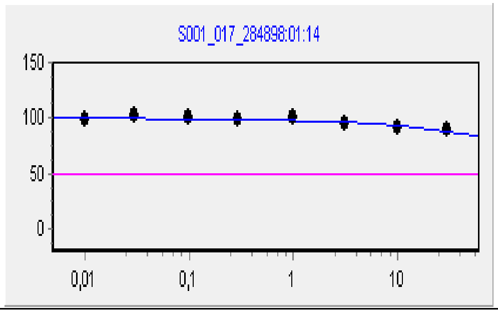	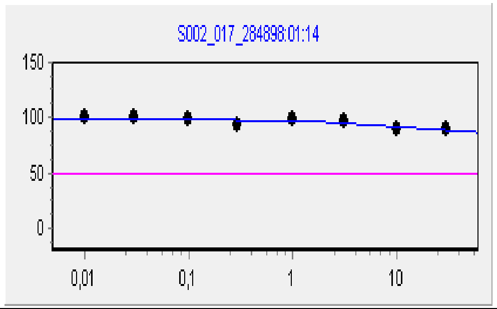	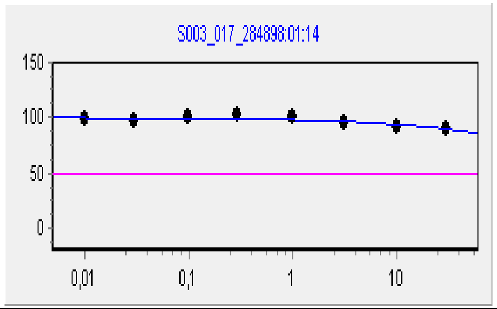	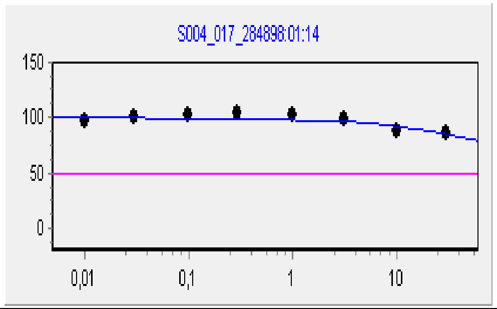

Table S4. (Part V)

Cpd.	IC_{50} curve–FP replicate 1	IC_{50} curve–FP replicate 2	IC_{50} curve–FP replicate 3	IC_{50} curve–FP replicate 4
C896	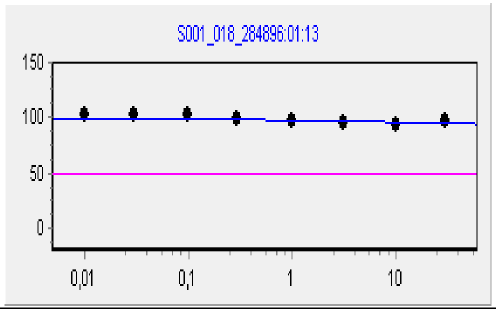	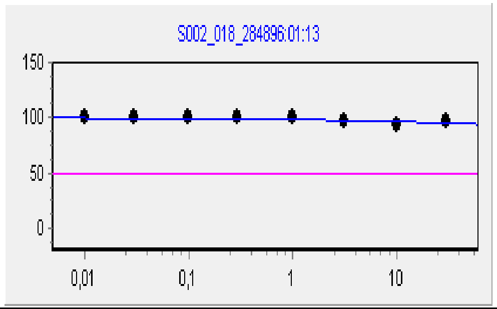	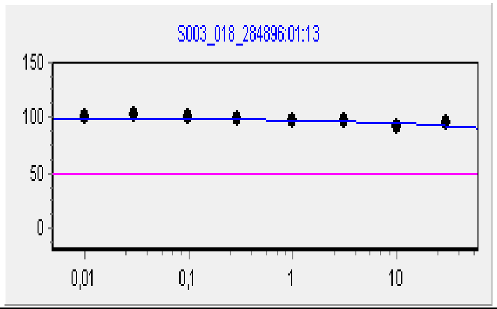	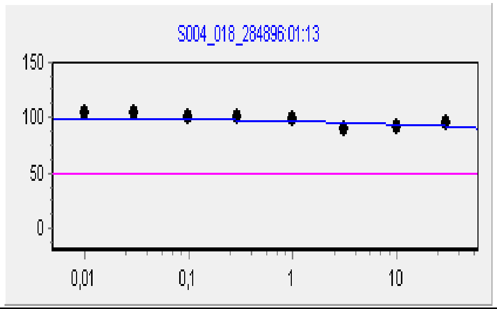
C893	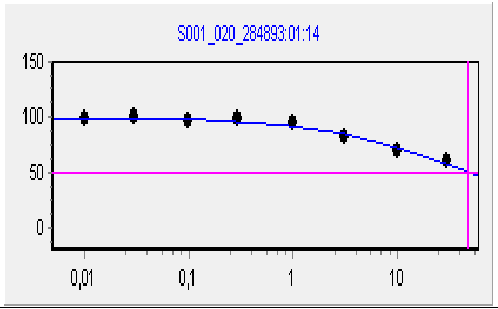	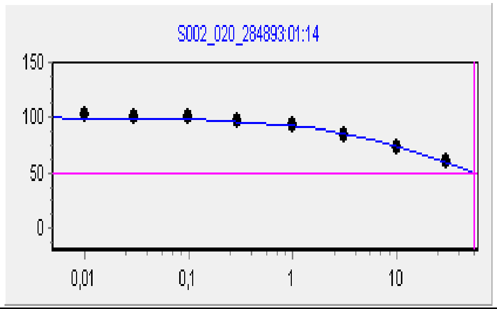	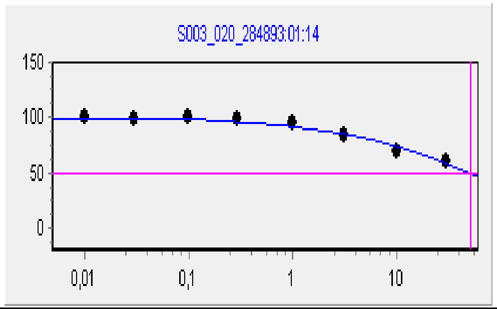	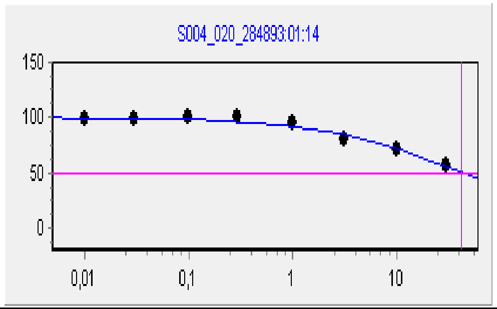
C883	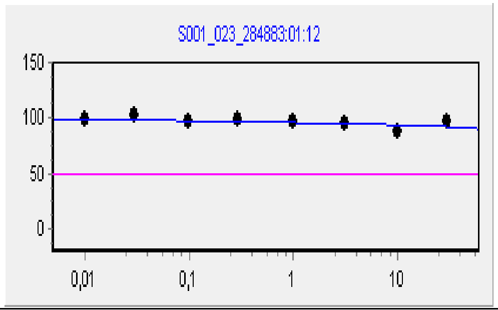	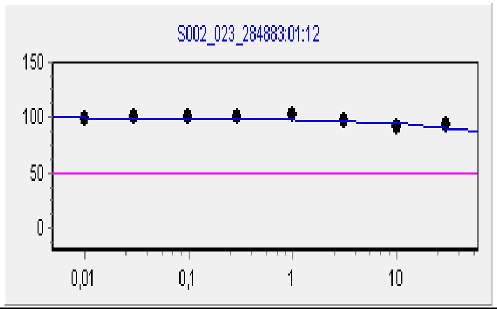	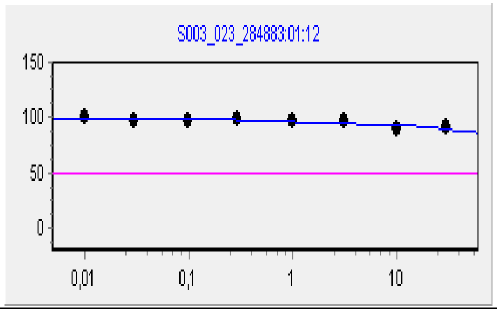	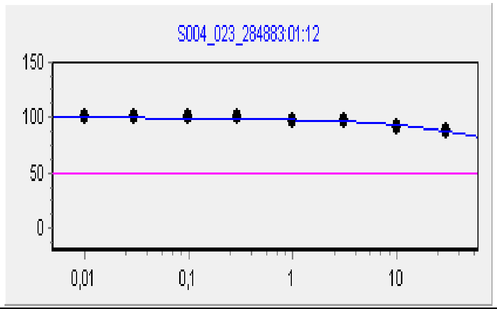
C899	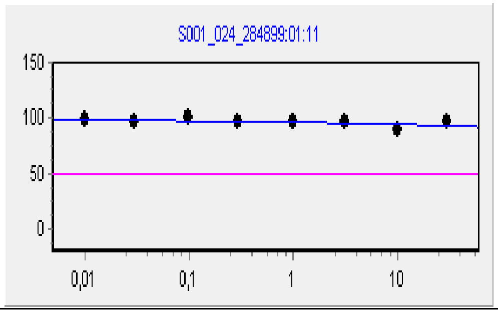	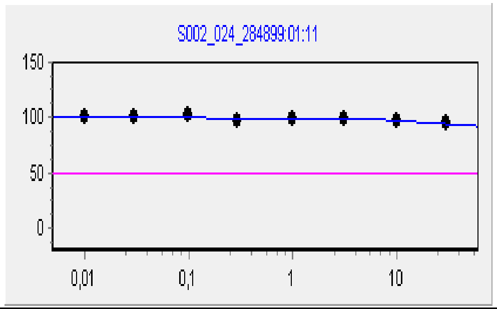	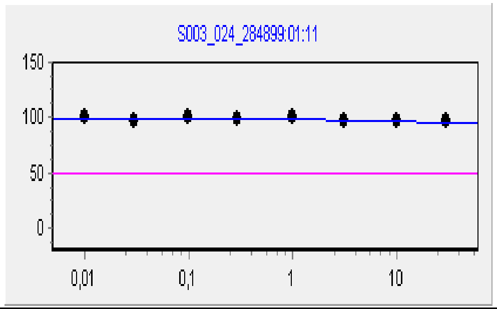	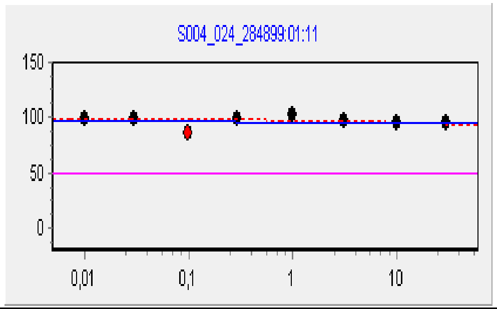

Table S4. (Part VI)

Cpd.	IC50 curve–FP replicate 1	IC50 curve–FP replicate 2	IC50 curve–FP replicate 3	IC50 curve–FP replicate 4
C894	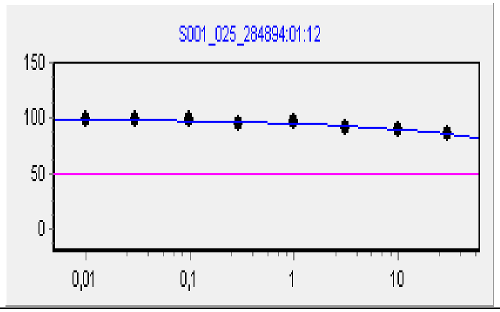	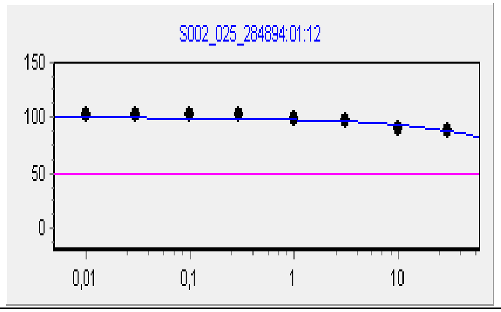	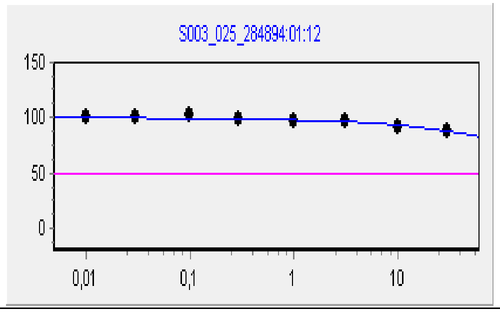	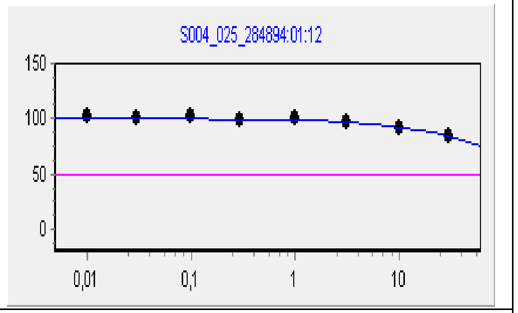
C891	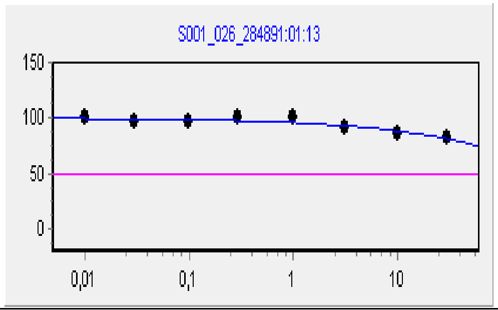	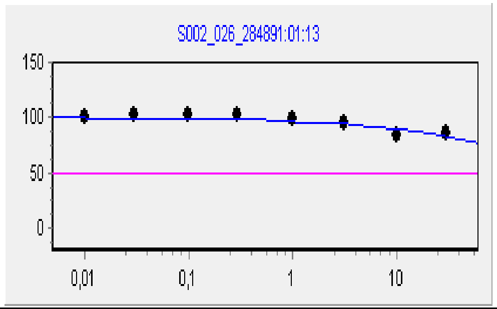	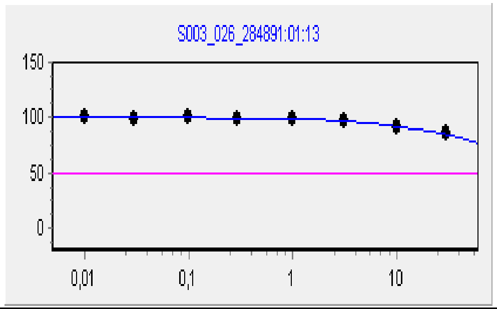	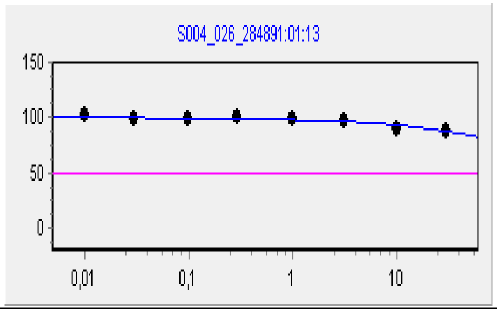
C908	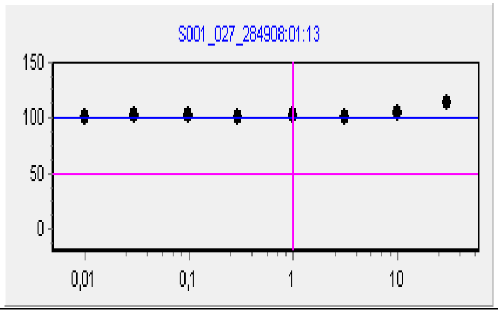	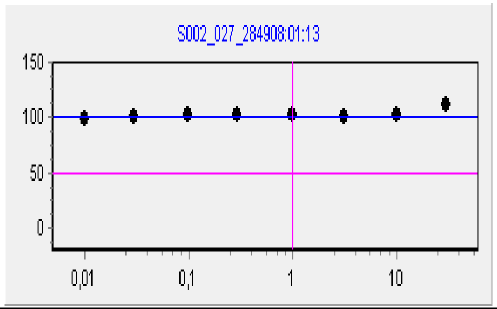	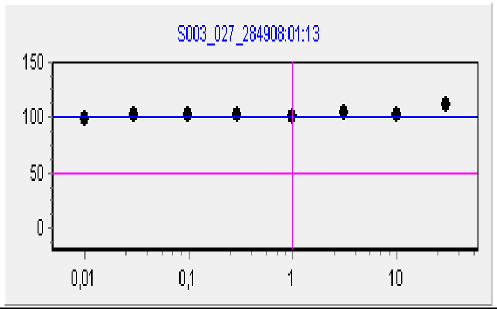	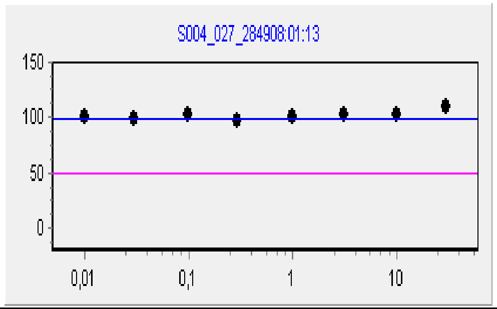
C906	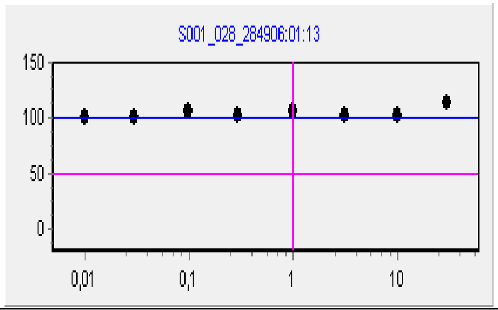	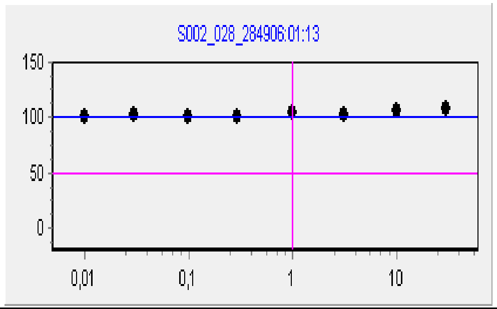	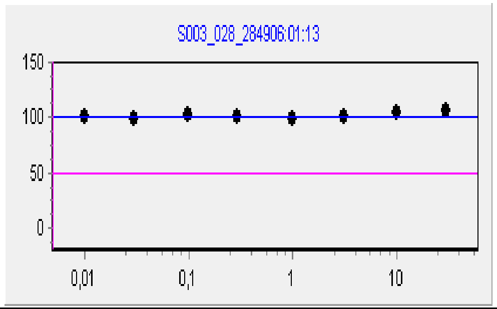	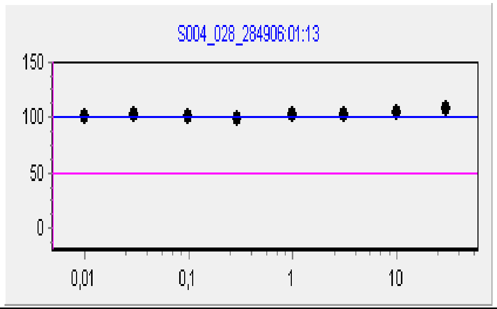

Table S4. (Part VII)

Cpd.	IC50 curve–FP replicate 1	IC50 curve–FP replicate 2	IC50 curve–FP replicate 3	IC50 curve–FP replicate 4
C888	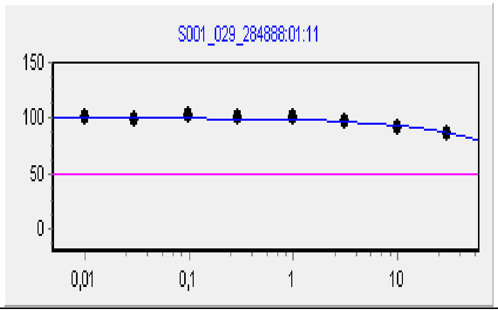	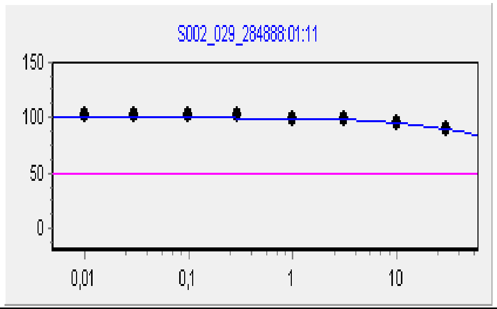	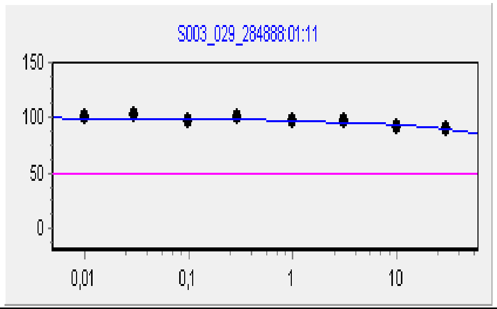	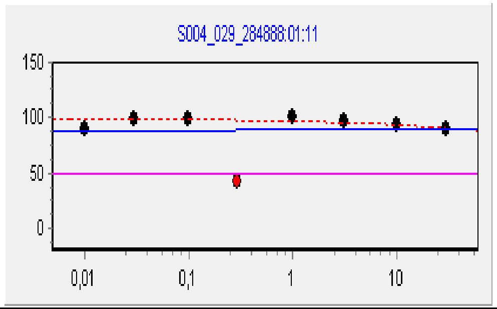

GENERAL CHEMISTRY INFORMATION

All commercially purchased reagents and solvents were used without further purification unless noted otherwise. Dry solvents were purchased from Fischer Scientific, VWR and/or Acros and used without further treatment. Oxygen and/or moisture sensitive solutions were transferred using syringes and cannulas under inert gas.

Thin layer chromatography (TLC) was performed on silica coated aluminum plates (Merck 60 F254) and visualization was achieved under UV irradiation (254 nm) or using potassium permanganate stain (1.5 g $KMnO_4$, 10 g K_2CO_3, 1.25 mL of 10% aqueous NaOH solution and 200 mL of water).

Analytical UHPLC-MS and LC-MS was performed on an Agilent 1260 II Infinity system equipped with a mass detector (UHPLC column: Zorbax Eclipse C18 Rapid Resolution 2.1x50 mm 1.8μm; LC-MS column: InfinityLab Poroshell 120 EC-C18, 2.1x150, 2.7 μm). Appropriate gradient systems obtained by mixing Acetonitrile (+ 0.1% TFA) and Water (+ 0.1% TFA) were used.

Purification of crude products was achieved through flash column chromatography (FC, silica gel 60 Å, 0.035-0.070 mm) or automated medium pressure liquid chromatography (MPLC, Buchi Pure C-810, Buchi Pure C-835) using the indicated solvents. Alternatively, separations were carried out on a Buchi Pure C-835 system (columns: Nuleodur C18 gravity VP 125/10 5 μm). Appropriate gradient systems obtained by mixing Acetonitrile (+ 0.1% TFA) and Water (+ 0.1% TFA) were used.

NMR spectra were recorded on Bruker AV 400 Avance III HD (NanoBay), Agilent Technologies DD2, Bruker AV 500 Avance III HD (Prodigy), Bruker AV 600 Avance III HD (CryoProbe), or Bruker AV 700 Avance III HD (CryoProbe) spectrometers. Data is reported in parts per million (ppm) with reference to the used deuterated solvent ($CDCl_3$: 7.26 ppm, 77.16 ppm; DMSO-d6: 2.50 ppm, 39.52 ppm; Acetone-d6: 2.05 ppm, 29.84 ppm, 206.26 ppm). Chemical shift value is reported in ppm, multiplicity (s = singlet, d = doublet, t = triplet, dd = double doublet, and m = multiplet), integration

value, and coupling constant value in Hz. Signals were assigned to corresponding Hydrogens or Carbons based on 2D NMR correlations ($^{1}H/^{1}H$ COSY, $^{1}H/^{1}H$ NOESY, $^{1}H/^{13}C$ HSQC, $^{1}H/^{13}C$ HMBC).

High-resolution mass spectrometry (HRMS) was measured on an LTQ Orbitrap mass spectrometer coupled to an Accela HPLC-System (HPLC column: Hypersyl GOLD, 50 mm x 1 mm, particle size 1.9 μm, ionization method: electron spray ionization (ESI)).

SYNTHETIC PROCEDURES AND COMPOUND CHARACTERIZATION

General step for the Doebner condensation

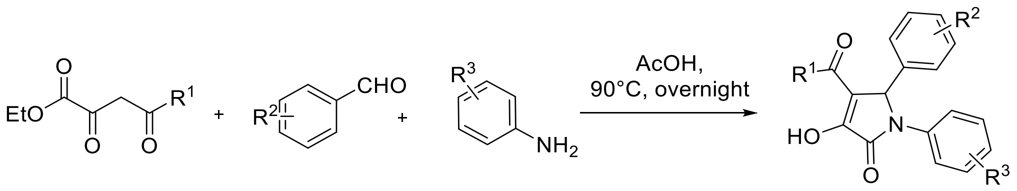

The benzaldehyde derivative (1.0 equiv.) and the aniline derivative (1.0 equiv.) were added sequentially to a suspension (0.06 M) of the dioxobutanoate component (1.0 equiv.) in acetic acid.[3] The reaction mixture was stirred overnight at 90 °C. After cooled to room temperature, the reaction mixture was diluted with Et_2O and filtered. The residue was washed with Et_2O to give the desired pyrrolinone derivative after drying. In the case that precipitation did not yield pure product, the precipitate was further purified with an appropriate gradient on a preparative HPLC system.

4-(3-(4-Bromobenzoyl)-4-hydroxy-5-oxo-2-(4-(thiazol-2-yl)phenyl)-2,5-dihydro-1*H*-pyrrol-1-yl)-2-hydroxybenzoic acid (**PH-30**)

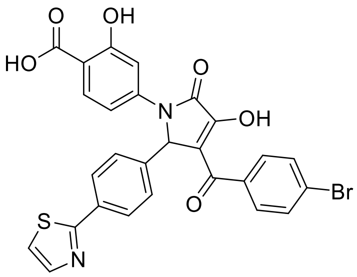

Purified by preparative HPLC, obtained the target compound as a brown solid (18.1 mg). **^{1}H NMR** (600 MHz, Methanol-d_4) δ 8.02 (d, J = 2.5 Hz, 1H), 7.80 (dd, J = 9.8, 5.6 Hz, 3H), 7.71 (d, J = 8.0 Hz, 2H), 7.61 (d, J = 7.6 Hz, 3H), 7.55 (d, J = 3.1 Hz, 1H), 7.46 (d, J = 7.9 Hz, 2H), 6.87 (d, J = 8.8

Hz, 1H), 6.21 (s, 1H). **^{13}C NMR** (151 MHz, Methanol-d_4) δ 190.3, 172.9, 169.5, 166.3, 163.1, 161.3, 144.4, 139.8, 138.5, 134.6, 132.5, 132.0, 131.8, 129.8, 128.9, 128.5, 127.9, 126.7, 121.0, 120.7, 118.7, 114.2, 63.8. **R_f**: 0,6 in 30% MeOH in DCM. **HRMS**-ESI (m/z): calculated for $C_{27}H_{18}N_2O_6BrS$ $[M+H]^+$ 577.0064; found, 577.0062. Calculated for $C_{27}H_{18}N_2O_6{}^{81}BrS$ $[M+H]^+$ 579.0043; found, 579.0041.

5-(3-(4-Bromobenzoyl)-4-hydroxy-5-oxo-2-(4-(thiazol-2-yl)phenyl)-2,5-dihydro-1*H*-pyrrol-1-yl)-2-hydroxybenzoic acid (C902/PH-31)

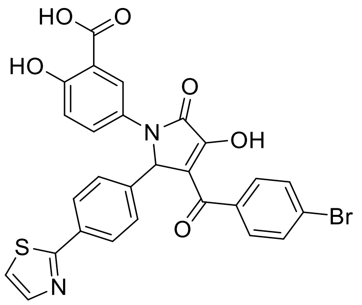

Yellow solid (119.6 mg, 31%). **^{1}H NMR** (500 MHz, Acetone-d_6) δ 8.20 (d, J = 2.7 Hz, 1H), 7.87 (d, J = 8.4 Hz, 2H), 7.80 (dt, J = 7.3, 3.3 Hz, 4H), 7.67 (d, J = 8.5 Hz, 2H), 7.60 (d, J = 8.3 Hz, 2H), 7.57 (d, J = 3.2 Hz, 1H), 6.92 (d, J = 9.0 Hz, 1H), 6.37 (s, 1H). **^{1}H NMR** (600 MHz, DMSO-d_6) δ 14.08 (s, 1H), 11.98 (s, 1H), 11.21 (s, 1H), 8.02 (d, J = 2.7 Hz, 1H), 7.86 (d, J = 3.2 Hz, 1H), 7.81 – 7.76 (m, 2H), 7.73 (d, J = 3.2 Hz, 1H), 7.72 – 7.63 (m, 5H), 7.52 – 7.48 (m, 2H), 6.91 (d, J = 8.9 Hz, 1H), 6.31 (s, 1H). **^{1}H NMR** (600 MHz, DMSO-d_6 + D_2O exchange) δ 8.01 (d, J = 4.7 Hz, 1H), 7.84 (q, J = 3.4, 2.7 Hz, 1H), 7.77 (d, J = 8.5 Hz, 3H), 7.71 – 7.63 (m, 5H), 7.48 (q, J = 4.8 Hz, 2H), 6.90 (dd, J = 9.0, 2.3 Hz, 1H), 6.31 – 6.25 (m, 1H). **^{13}C NMR** (126 MHz, Acetone-d_6) δ 188.9, 172.0, 167.7, 165.0, 160.6, 151.5, 144.7, 139.2, 138.0, 134.5, 132.3, 131.7, 131.6, 129.6, 129.1, 127.9, 127.3, 125.6, 120.5, 120.4, 118.4, 113.1, 62.8. **R_f**: 0.25 in 40% MeOH in DCM. **HRMS**-ESI (m/z): calculated for

$C_{27}H_{18}N_2O_6BrS$ [M+H]$^+$ 577.0064; found, 577.0063. Calculated for $C_{27}H_{18}N_2O_6{}^{81}BrS$ [M+H]$^+$ 579.0043; found, 579.0040.

5-(3-Benzoyl-4-hydroxy-5-oxo-2-(4-(thiazol-2-yl)phenyl)-2,5-dihydro-1*H*-pyrrol-1-yl)-2-hydroxybenzoic acid (PH-34)

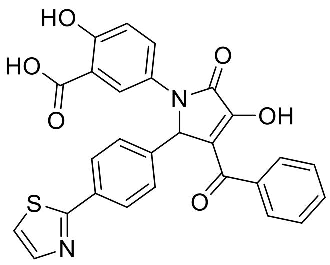

Purified by preparative HPLC, obtained the target compound as a yellow solid (12.1 mg). **^{1}H NMR** (600 MHz, DMSO-d_6) δ 14.14 (s, 1H), 11.97 (s, 1H), 11.17 (s, 1H), 8.03 (d, J = 2.7 Hz, 1H), 7.85 (d, J = 3.2 Hz, 1H), 7.81 – 7.76 (m, 2H), 7.75 – 7.68 (m, 4H), 7.60 – 7.54 (m, 1H), 7.53 – 7.42 (m, 4H), 6.91 (d, J = 9.0 Hz, 1H), 6.33 (s, 1H). **^{13}C NMR** (151 MHz, DMSO-d_6) δ 189.2, 171.2, 166.4, 164.5, 158.7, 150.4, 143.8, 138.5, 137.9, 132.7, 130.6, 128.7, 128.7, 128.2, 127.7, 126.3, 124.9, 120.6, 119.6, 117.5, 112.9, 61.3. **R$_f$**: 0.32 in 40% MeOH in DCM + 0.1% AcOH. **HRMS**-ESI (m/z): calculated for $C_{27}H_{19}N_2O_6S$ [M+H]$^+$ 499.0958; found, 499.0953.

4-(4-Bromobenzoyl)-3-hydroxy-1-(4-hydroxyphenyl)-5-(4-(thiazol-2-yl)phenyl)-1,5-dihydro-2*H*-pyrrol-2-one (PH-35)

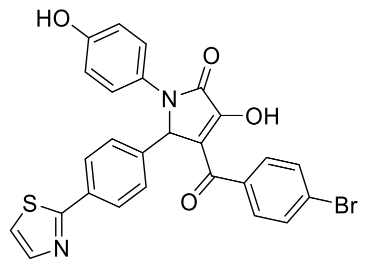

Grey solid (82.7 mg, 34%). **^{1}H NMR** (500 MHz, DMSO-*d_6*) δ 12.15 (s, 1H), 9.47 (s, 1H), 7.86 (d, J = 3.2 Hz, 1H), 7.77 (d, J = 8.3 Hz, 2H), 7.73 (d, J = 3.2 Hz, 1H), 7.69 – 7.62 (m, 4H), 7.47 (d, J = 8.3 Hz, 2H), 7.36 (d, J = 8.9 Hz, 2H), 6.68 (d, J = 8.9 Hz, 2H), 6.22 (s, 1H). **^{13}C NMR** (176 MHz, DMSO-*d_6*) δ 208.0, 187.9, 166.4, 164.1, 155.3, 143.7, 138.9, 137.1, 132.5, 131.2, 130.6, 128.6, 127.6, 126.3, 126.1, 124.7, 120.4, 115.2, 61.3. **R_f**: 0.5 in 20% MeOH in DCM + 0.1% AcOH. **HRMS**-ESI (m/z): calculated for $C_{26}H_{18}N_2O_4BrS$ $[M+H]^+$ 533.0165; found, 533.0163. Calculated for $C_{26}H_{18}N_2O_4{}^{81}BrS$ $[M+H]^+$ 535.0145; found, 535.0141.

4-(3-Benzoyl-4-hydroxy-1-(4-hydroxyphenyl)-5-oxo-2,5-dihydro-1*H*-pyrrol-2-yl)benzoic acid (PH-36)

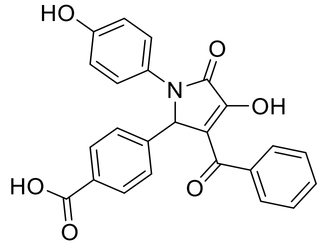

Yellow solid (104.6 mg, 55%). **^{1}H NMR** (600 MHz, DMSO-*d_6*) δ 12.75 (s, 1H), 11.95 (s, 1H), 9.45 (s, 1H), 7.75 (d, J = 8.1 Hz, 2H), 7.69 (d, J = 7.3 Hz, 2H), 7.55 (t, J = 7.4 Hz, 1H), 7.48 – 7.41 (m, 4H), 7.36 – 7.33 (m, 2H), 6.75 – 6.54 (m, 2H), 6.24 (s, 1H). **^{13}C NMR** (151 MHz, DMSO-*d_6*) δ 189.0, 166.8, 164.3, 155.3, 138.1, 132.5, 130.3, 129.8, 129.3, 128.7, 128.7, 128.1, 128.0, 127.6, 127.5, 124.7,

115.2, 61.4. **R$_f$**: 0.5 in 40% MeOH in DCM + 0.1% AcOH. **HRMS**-ESI (m/z): calculated for $C_{24}H_{18}NO_6$ $[M+H]^+$ 416.1129; found, 416.1127.

4-Benzoyl-3-hydroxy-1-(4-hydroxy-3-nitrophenyl)-5-(4-(thiazol-2-yl)phenyl)-1,5-dihydro-2*H*-pyrrol-2-one (PH-37)

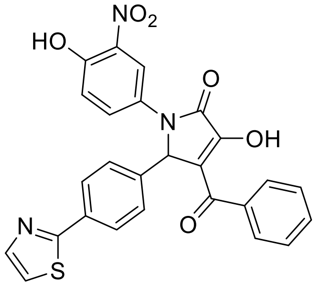

Purified by preparative HPLC, obtained the target compound as a yellow solid (2.0 mg). **^{1}H NMR** (500 MHz, DMSO-*d*$_6$) δ 12.05 (s, 1H), 11.04 (s, 1H), 8.25 (d, J = 2.6 Hz, 1H), 7.86 (d, J = 3.2 Hz, 1H), 7.82 – 7.77 (m, 2H), 7.77 – 7.70 (m, 4H), 7.60 – 7.52 (m, 3H), 7.46 (t, J = 7.8 Hz, 2H), 7.07 (d, J = 9.1 Hz, 1H), 6.39 (s, 1H). **^{13}C NMR** (126 MHz, DMSO-*d*$_6$) δ 189.3, 166.4, 164.6, 150.2, 149.6, 143.9, 138.2, 137.8, 136.3, 132.8, 129.7, 129.2, 128.8, 128.8, 128.3, 127.7, 126.3, 120.7, 119.8, 119.3, 119.2, 61.1. **R$_f$**: 0.28 in 10% MeOH in DCM + 0.1% AcOH. **HRMS**-ESI (m/z): calculated for $C_{26}H_{18}N_3O_6S$ $[M+H]^+$ 500.0911; found, 500.0906.

4-(3-Benzoyl-2-(4-carboxyphenyl)-4-hydroxy-5-oxo-2,5-dihydro-1*H*-pyrrol-1-yl)-2-hydroxybenzoic acid (PH-38)

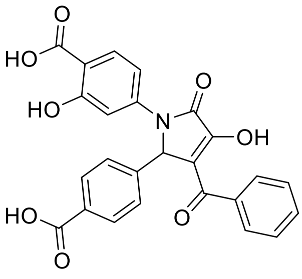

Purified by preparative HPLC, obtained the target compound as a white powder (3.0 mg). **^{1}H NMR** (500 MHz, DMSO-d_6) δ 13.90 (s, 1H), 12.90 (s, 1H), 12.08 (s, 1H), 11.29 (s, 1H), 7.78 (d, J = 8.3 Hz, 2H), 7.69 (d, J = 8.6 Hz, 3H), 7.56 (dd, J = 7.8, 3.5 Hz, 3H), 7.45 (t, J = 7.7 Hz, 2H), 7.32 (d, J = 2.1 Hz, 1H), 7.26 (dd, J = 8.7, 2.1 Hz, 1H), 6.44 (s, 1H). **^{13}C NMR** (126 MHz, DMSO-d_6) δ 178.7, 171.3, 166.8, 165.2, 161.4, 142.3, 141.4, 137.7, 132.9, 130.8, 130.5, 129.4, 128.8, 128.8, 128.3, 127.9, 120.1, 112.5, 109.6, 109.2, 60.6. **R$_f$**: 0.17 in 40% MeOH in DCM + 0.1% AcOH. **HRMS**-ESI (m/z): calculated for $C_{25}H_{18}NO_8$ $[M+H]^+$ 460.1027; found, 460.1025.

5-(3-Benzoyl-2-(4-carboxyphenyl)-4-hydroxy-5-oxo-2,5-dihydro-1*H*-pyrrol-1-yl)-2-hydroxybenzoic acid (C880/PH-39)

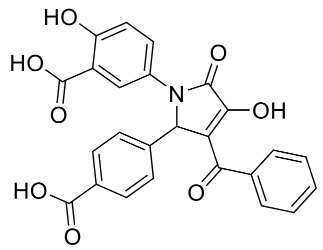

Purified by preparative HPLC, obtained the target compound as a white powder (3.8 mg). **^{1}H NMR** (500 MHz, Acetone-d_6) δ 8.18 (d, J = 2.7 Hz, 1H), 7.91 – 7.82 (m, 4H), 7.78 (dd, J = 9.0, 2.8 Hz, 1H), 7.58 (dd, J = 19.9, 7.9 Hz, 3H), 7.46 (t, J = 7.7 Hz, 2H), 6.90 (d, J = 9.0 Hz, 1H), 6.41 (s, 1H). **^{13}C NMR** (126 MHz, Acetone-d_6) δ 190.0, 172.2, 170.8, 167.0, 165.1, 160.6, 142.5, 139.0, 133.6, 131.5, 131.4, 130.6, 129.8, 129.1, 129.0, 125.6, 120.7, 118.4, 113.2, 62.9. **R$_f$**: 0.38 in 40% MeOH in DCM + 0.1% AcOH. **HRMS-**ESI (m/z): calculated for $C_{25}H_{18}NO_8$ $[M+H]^+$ 460.1027; found, 460.1024.

5-(3-(Furan-2-carbonyl)-4-hydroxy-5-oxo-2-(4-(thiazol-2-yl)phenyl)-2,5-dihydro-1H-pyrrol-1-yl)-2-hydroxybenzoic acid (PH-43)

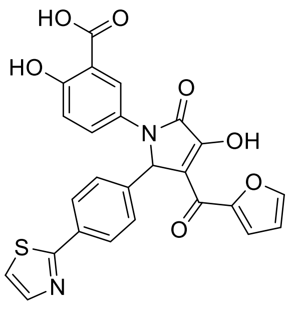

Purified by preparative HPLC, obtained the target compound as a yellow solid (20.2 mg). **^{1}H NMR** (600 MHz, Acetone-*d*$_6$) δ 10.98 (s, 1H), 8.14 (d, J = 2.7 Hz, 1H), 7.90 – 7.84 (m, 3H), 7.81 (d, J = 3.2 Hz, 1H), 7.77 (dd, J = 9.0, 2.7 Hz, 1H), 7.59 – 7.53 (m, 4H), 6.92 (d, J = 9.0 Hz, 1H), 6.67 (dd, J = 3.6, 1.7 Hz, 1H), 6.50 (s, 1H). **^{13}C NMR** (151 MHz, Acetone-*d*$_6$) δ 175.3, 171.1, 166.8, 163.0, 159.9, 155.7, 151.5, 147.9, 143.8, 138.5, 133.6, 131.5, 128.9, 128.1, 126.4, 125.3, 119.9, 119.6, 118.3, 117.6, 112.6, 112.1, 61.8. **HRMS-**ESI (m/z): calculated for $C_{25}H_{17}N_2O_7S$ $[M+H]^+$ 489.0751; found, 489.0746.

5-(3-(3,4-Dimethoxybenzoyl)-4-hydroxy-5-oxo-2-(4-(thiazol-2-yl)phenyl)-2,5-dihydro-1*H*-pyrrol-1-yl)-2-hydroxybenzoic acid (PH-44)

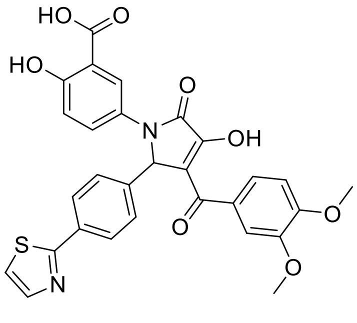

Purified by preparative HPLC, obtained the target compound as a yellow solid (14.7 mg). **^{1}H NMR** (600 MHz, Acetone-*d*$_6$) δ 8.20 (d, J = 2.7 Hz, 1H), 7.86 (d, J = 8.4 Hz, 2H), 7.82 (dd, J = 9.0, 2.8 Hz, 1H), 7.80 (d, J = 3.2 Hz, 1H), 7.62 (dd, J = 8.4, 2.0 Hz, 1H), 7.59 – 7.54 (m, 3H), 7.44 (d, J = 2.0 Hz,

1H), 7.00 (d, J = 8.5 Hz, 1H), 6.92 (d, J = 9.0 Hz, 1H), 6.39 (s, 1H), 3.87 (s, 3H), 3.84 (s, 3H). **^{13}C NMR** (151 MHz, Acetone-d_6) δ 187.5, 171.1, 166.8, 164.3, 159.5, 153.9, 149.1, 143.8, 138.3, 133.6, 130.8, 130.4, 128.7, 128.5, 126.5, 124.6, 124.3, 120.5, 119.6, 117.5, 112.0, 111.6, 110.5, 62.2, 55.3, 55.2. **HRMS-**ESI (m/z): calculated for $C_{29}H_{23}N_2O_8S$ $[M+H]^+$ 559.1170; found, 559.1167.

ANALYTICAL UHPLC AND NMR SPECTRA

Analytical UHPLC of PH-30:

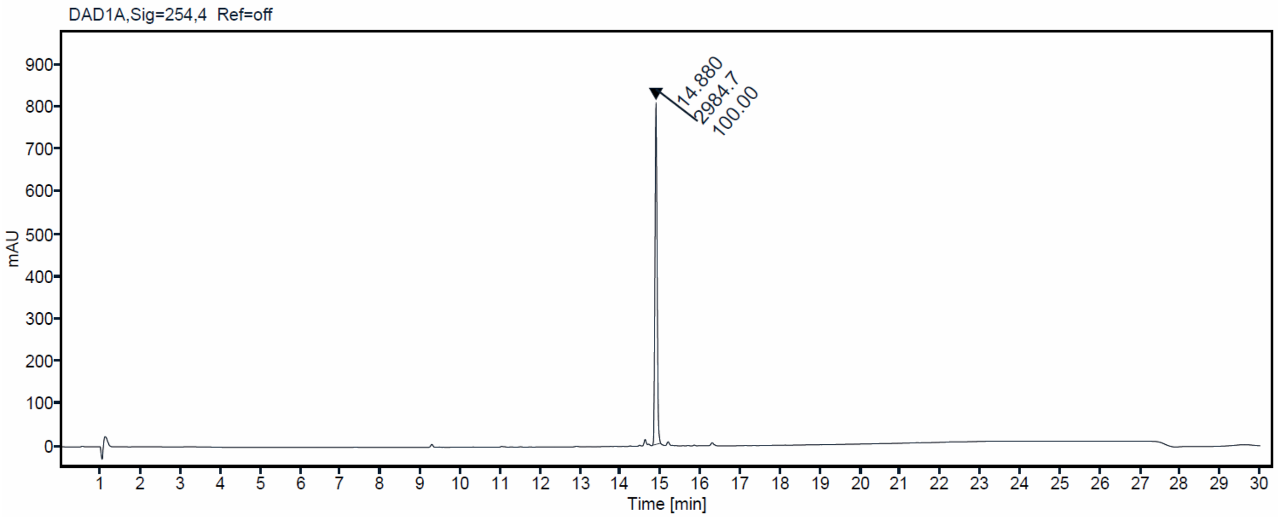

^{1}H NMR of PH-30 (600 MHz, Methanol-d_4), 15 ppm to -3 ppm:

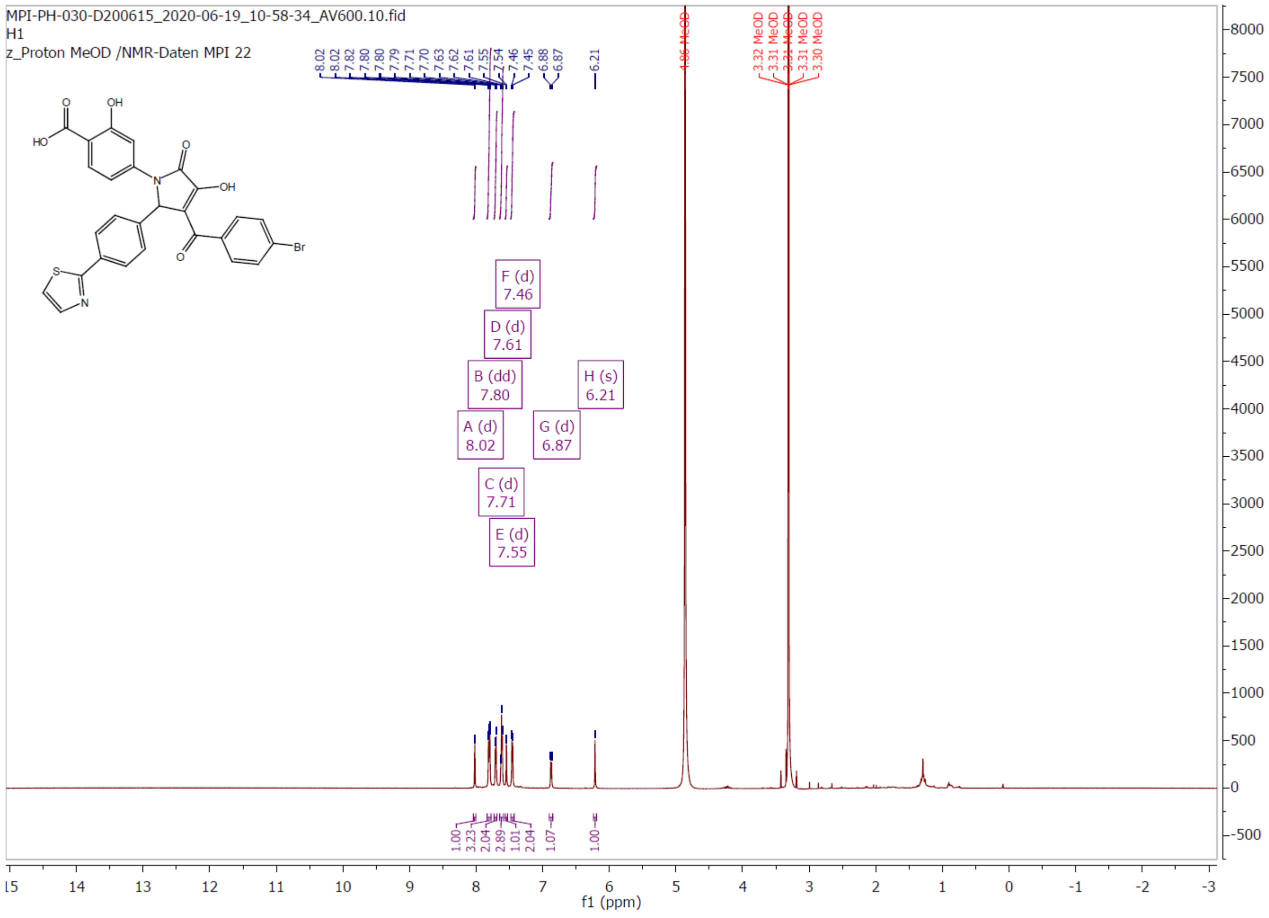

Partially enlarged ^{1}H NMR spectra of PH-30 (600 MHz, Methanol-d_4), 8.5 ppm to 6 ppm:

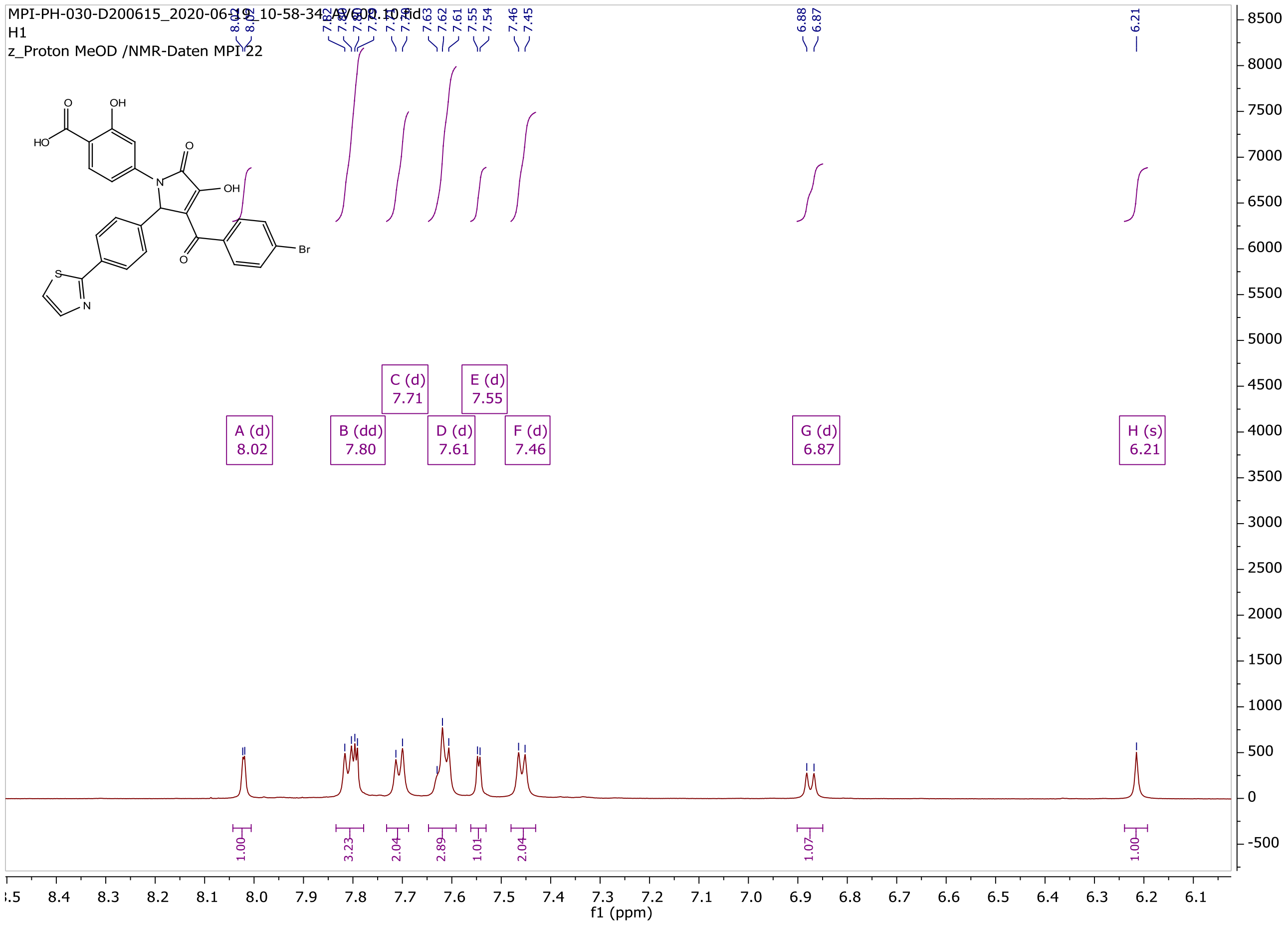

^{13}**C NMR of PH-30** (151 MHz, Methanol-d_4), 200 ppm to -10 ppm:

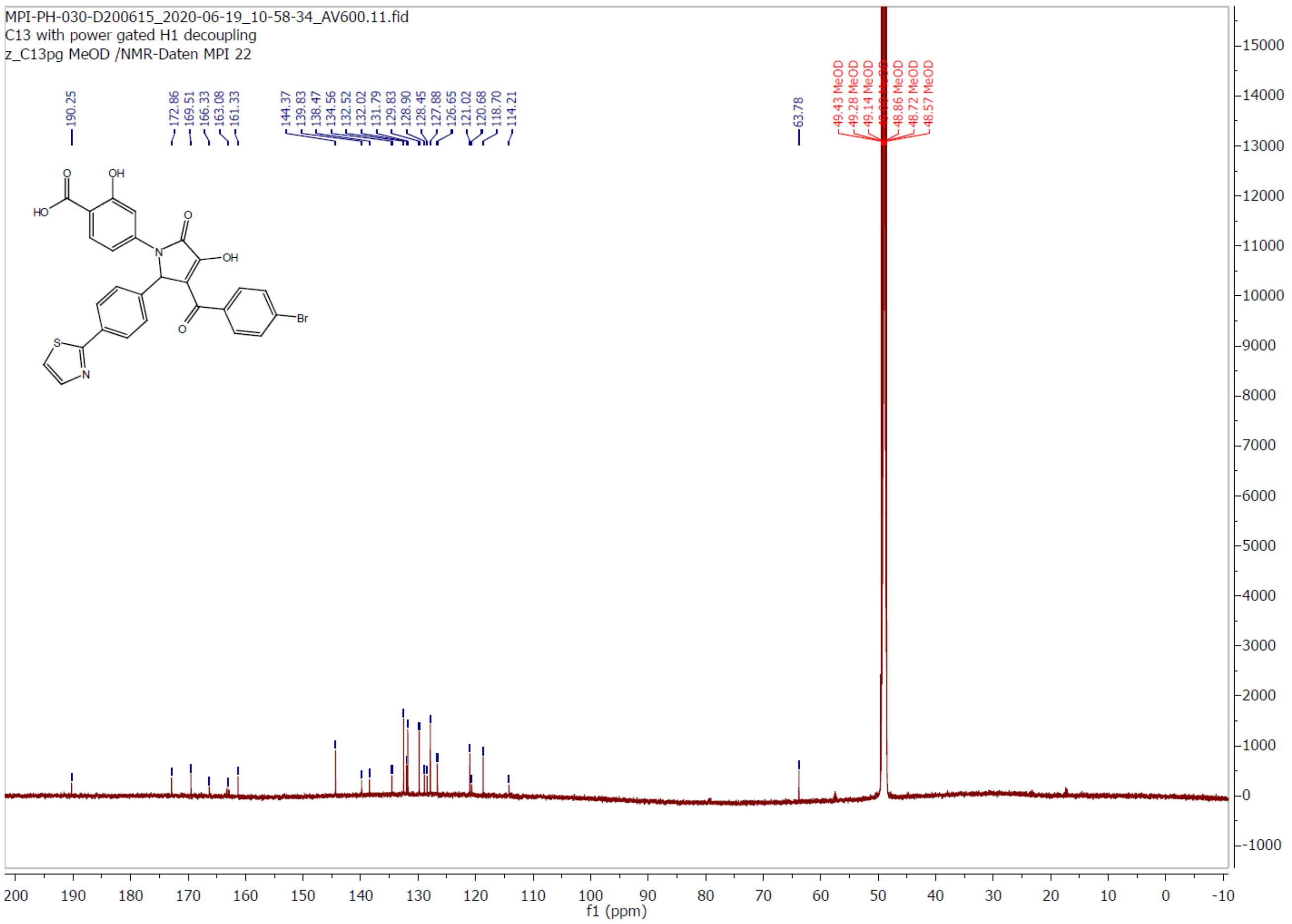

Analytical UHPLC, PH-31

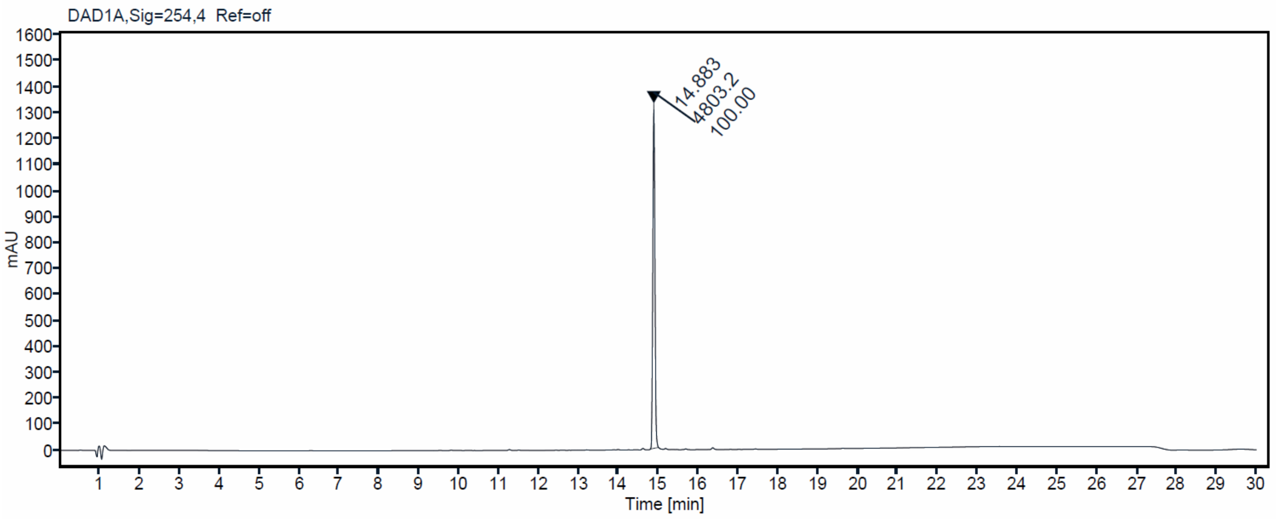

^{1}H NMR of PH-31 (500 MHz, Acetone-d_6), 16 ppm to -3 ppm:

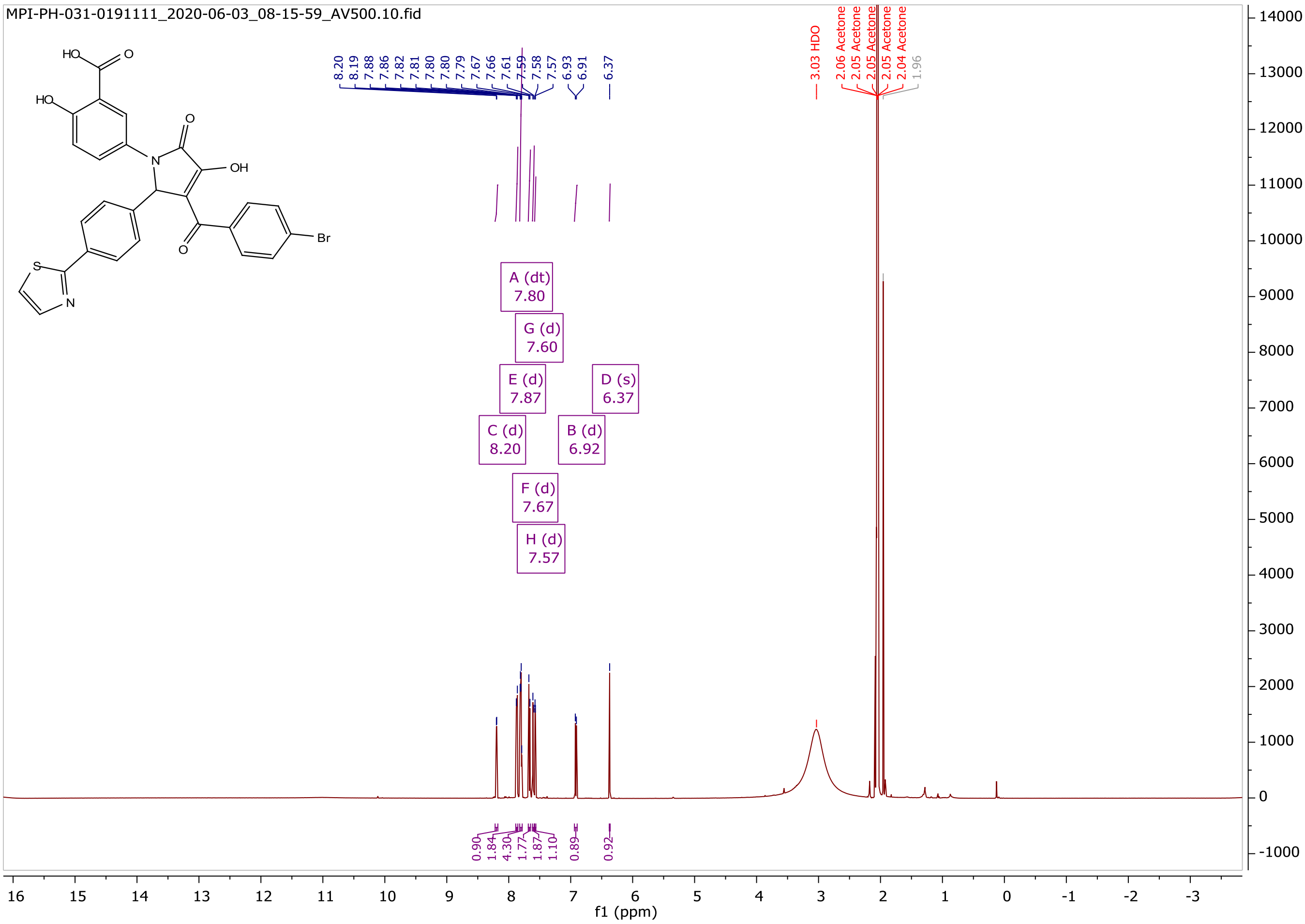

Partially enlarged ^{1}H NMR spectra of PH-31 (500 MHz, Acetone-d_6), 8.5 ppm to 6 ppm:

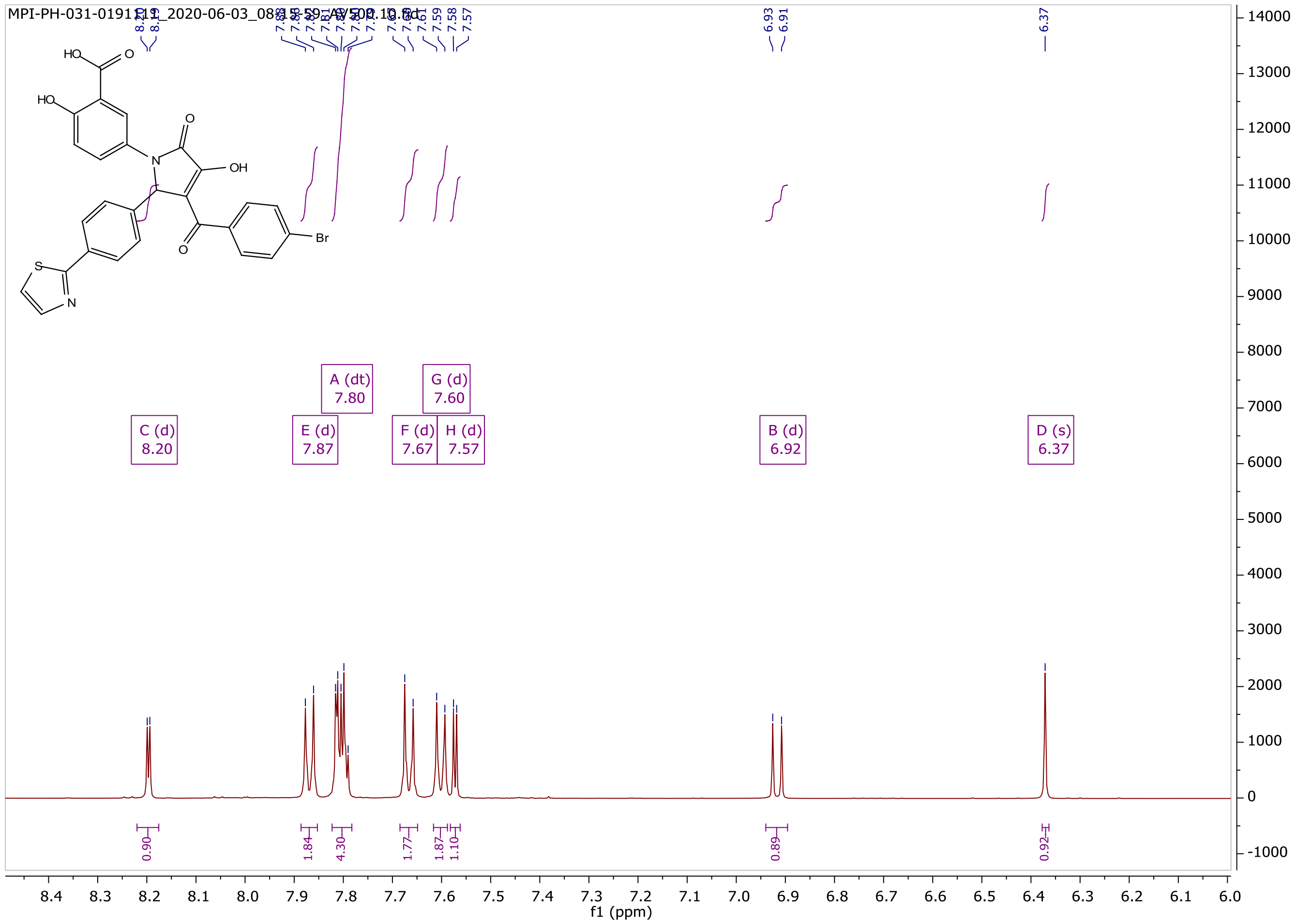

^{13}C NMR of PH-31 (126 MHz, Acetone-d_6), 210 ppm to -10 ppm:

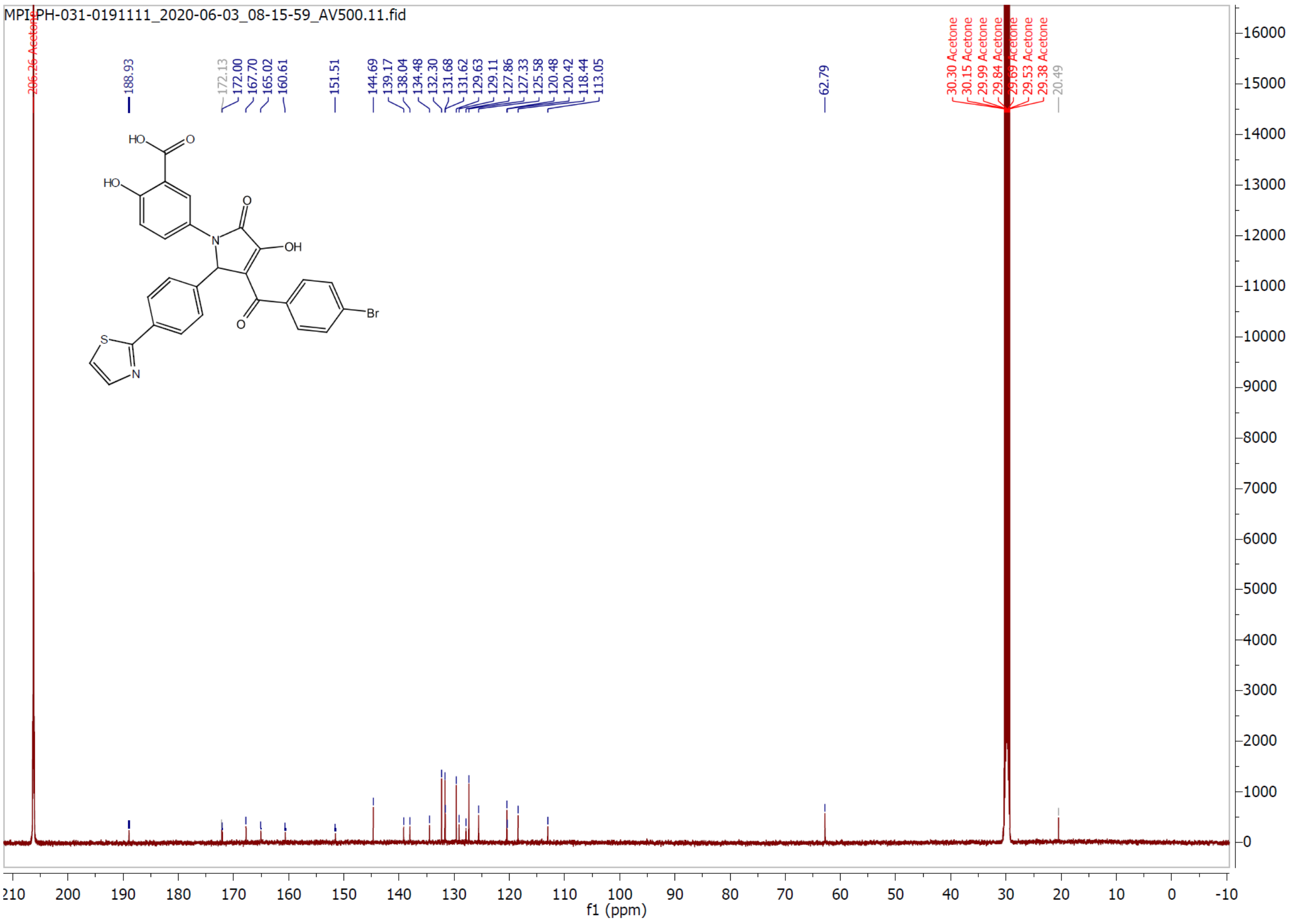

^{1}H NMR of PH-31 (600 MHz, DMSO-d_6), 16 to -4 ppm:

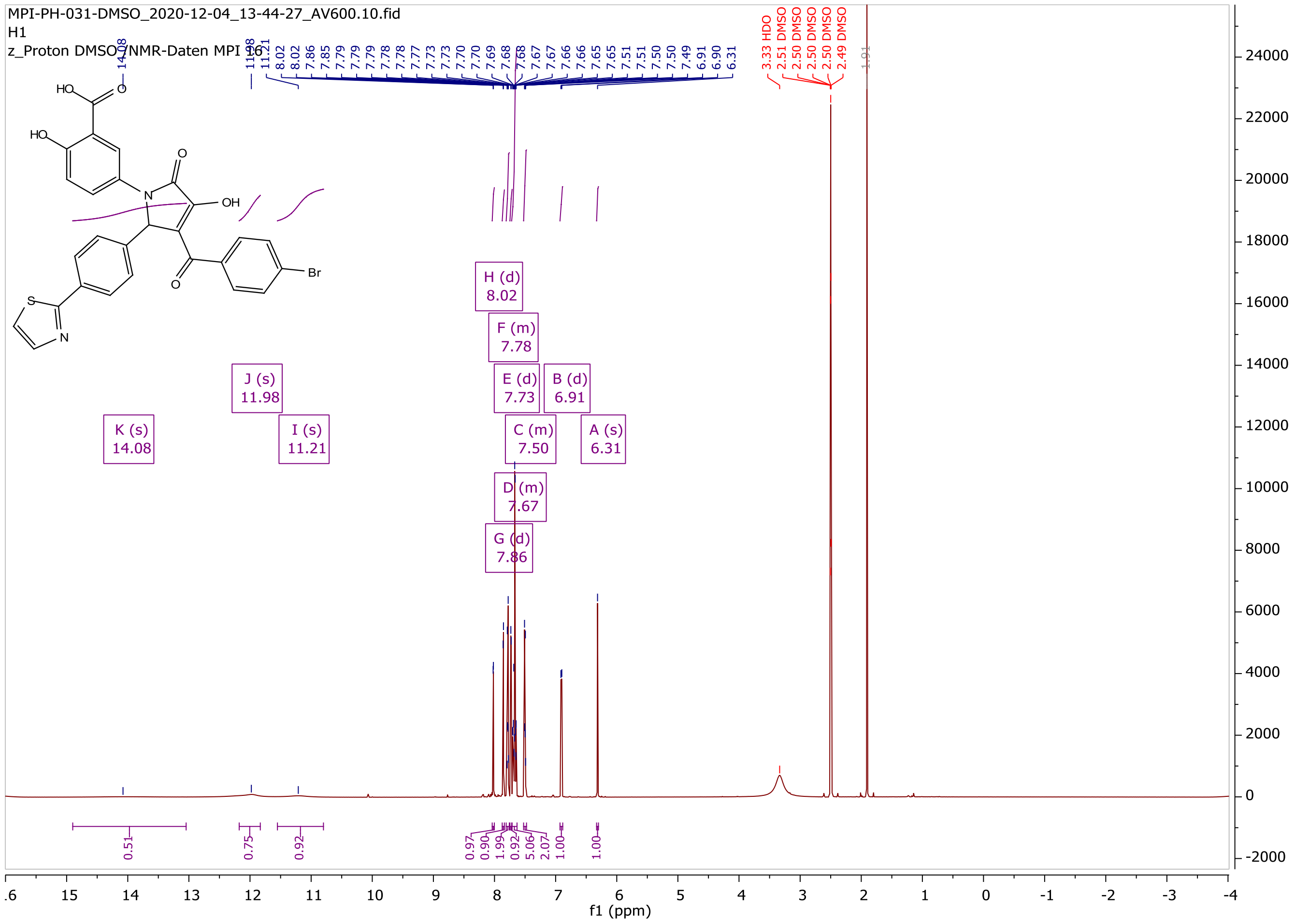

^{1}H NMR of PH-31 (600 MHz, DMSO-d_6 + D_2O exchange), 16 to -4 ppm:

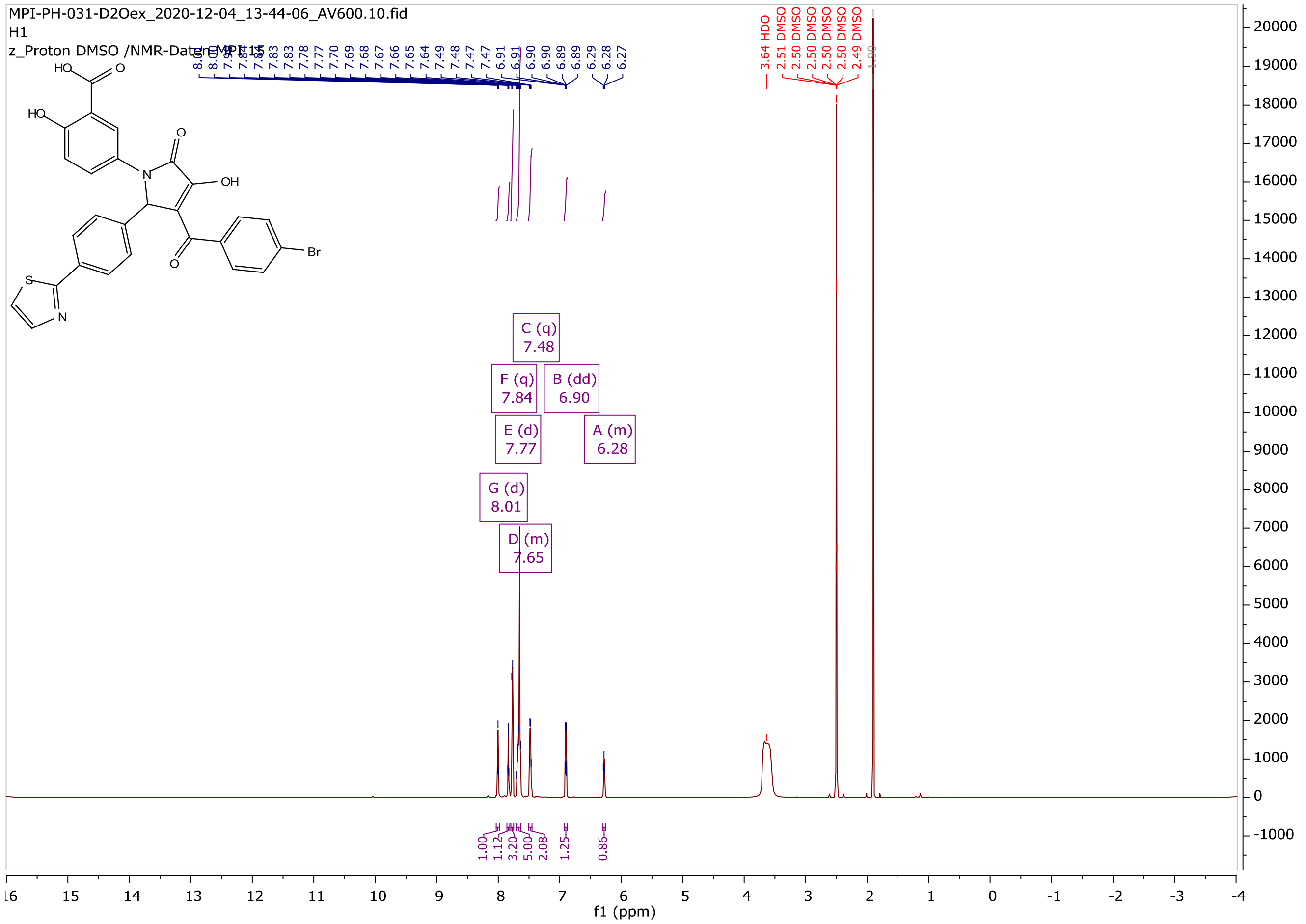

Analytical UHPLC, PH-34

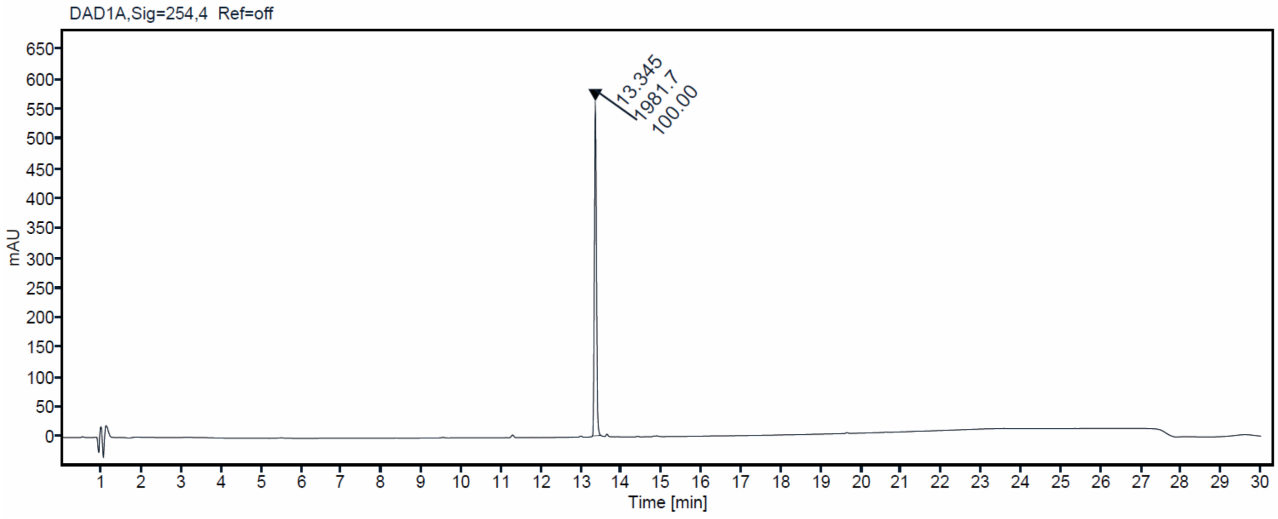

^{1}H NMR of PH-34 (600 MHz, DMSO-d_6), 16 ppm to -3 ppm:

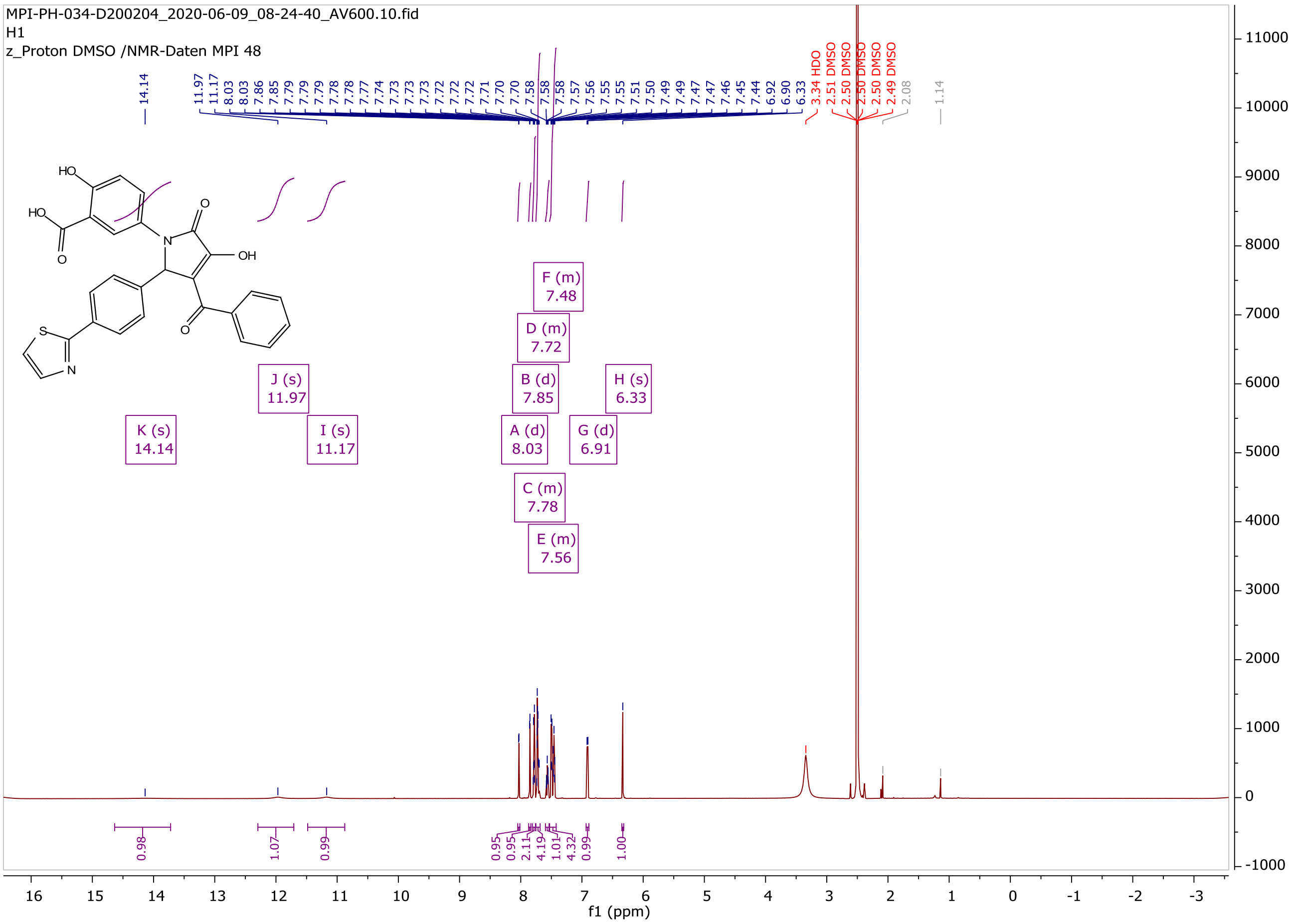

Partially enlarged ^{1}H NMR spectra of PH-34 (600 MHz, DMSO-d_6), 8.5 ppm to 6 ppm:

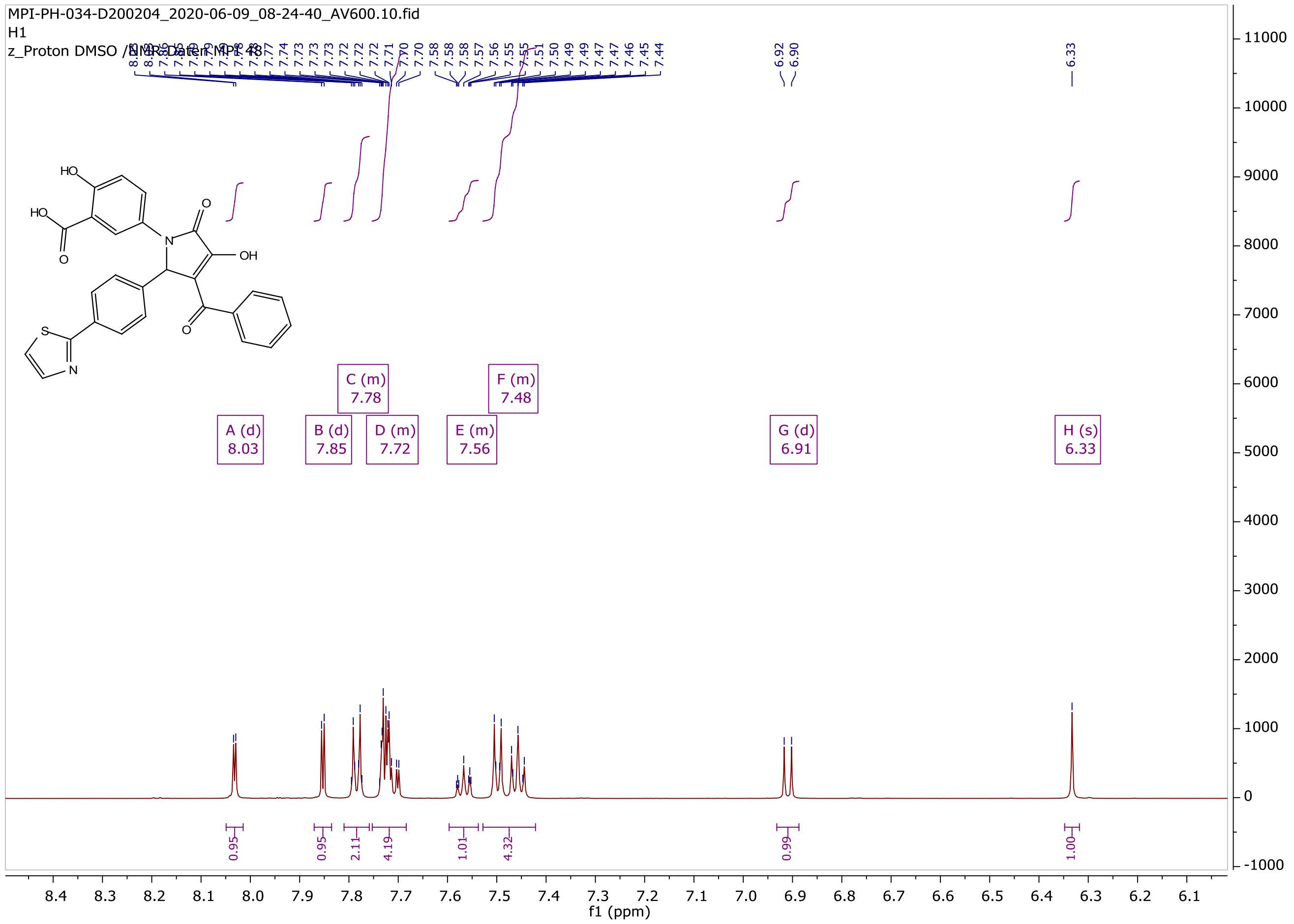

^{13}C NMR of PH-34 (151 MHz, DMSO-d_6), 210 ppm to -10 ppm:

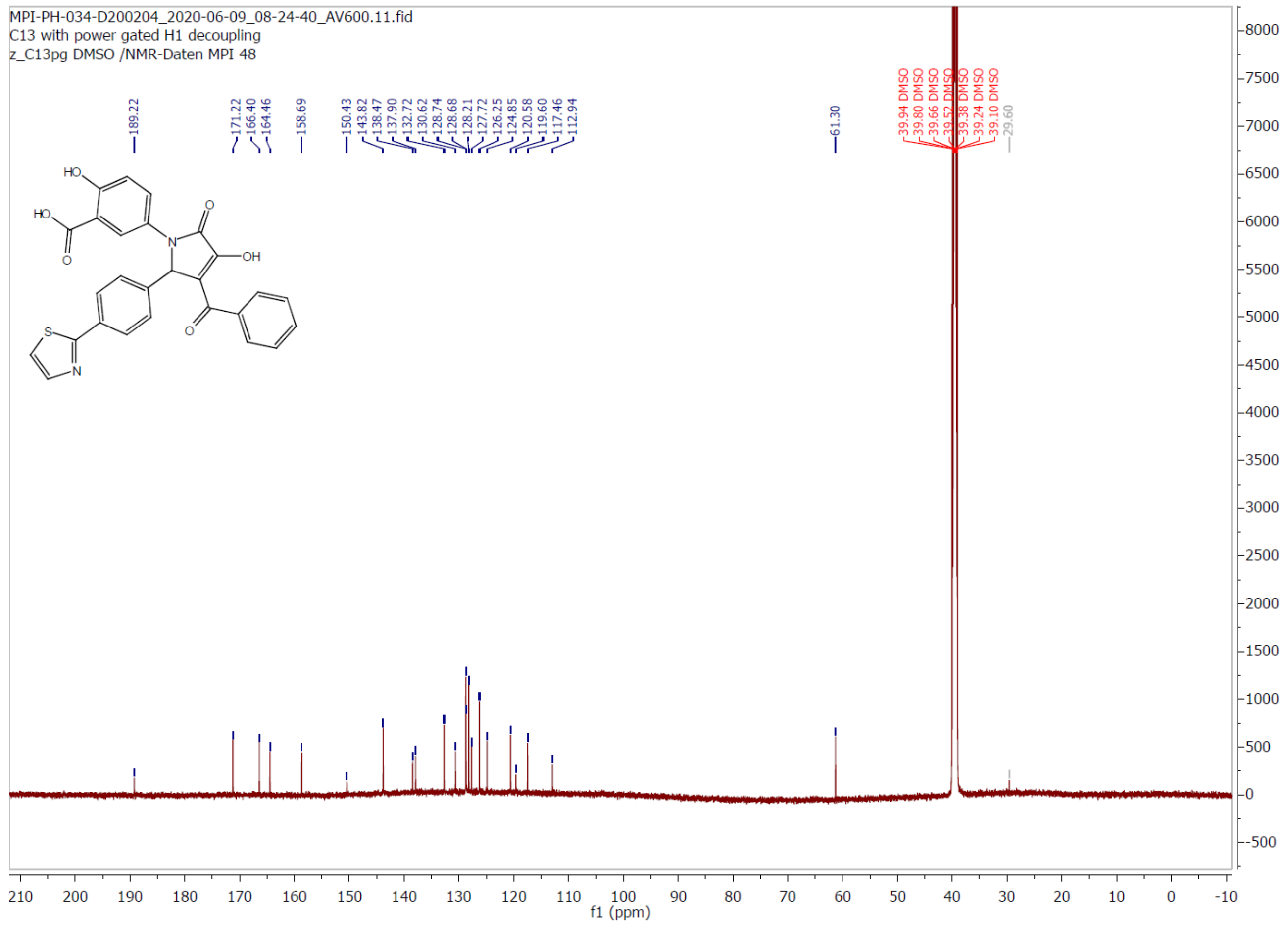

Analytical UHPLC, PH-35

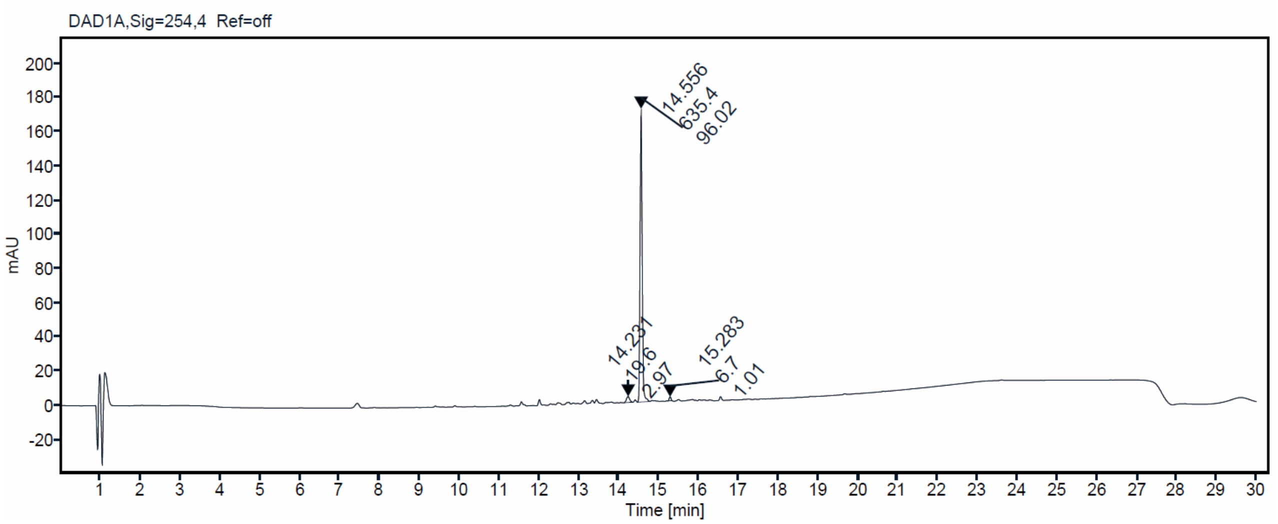

^{1}H NMR of PH-35 (500 MHz, DMSO-d_6), 16 ppm to -3 ppm:

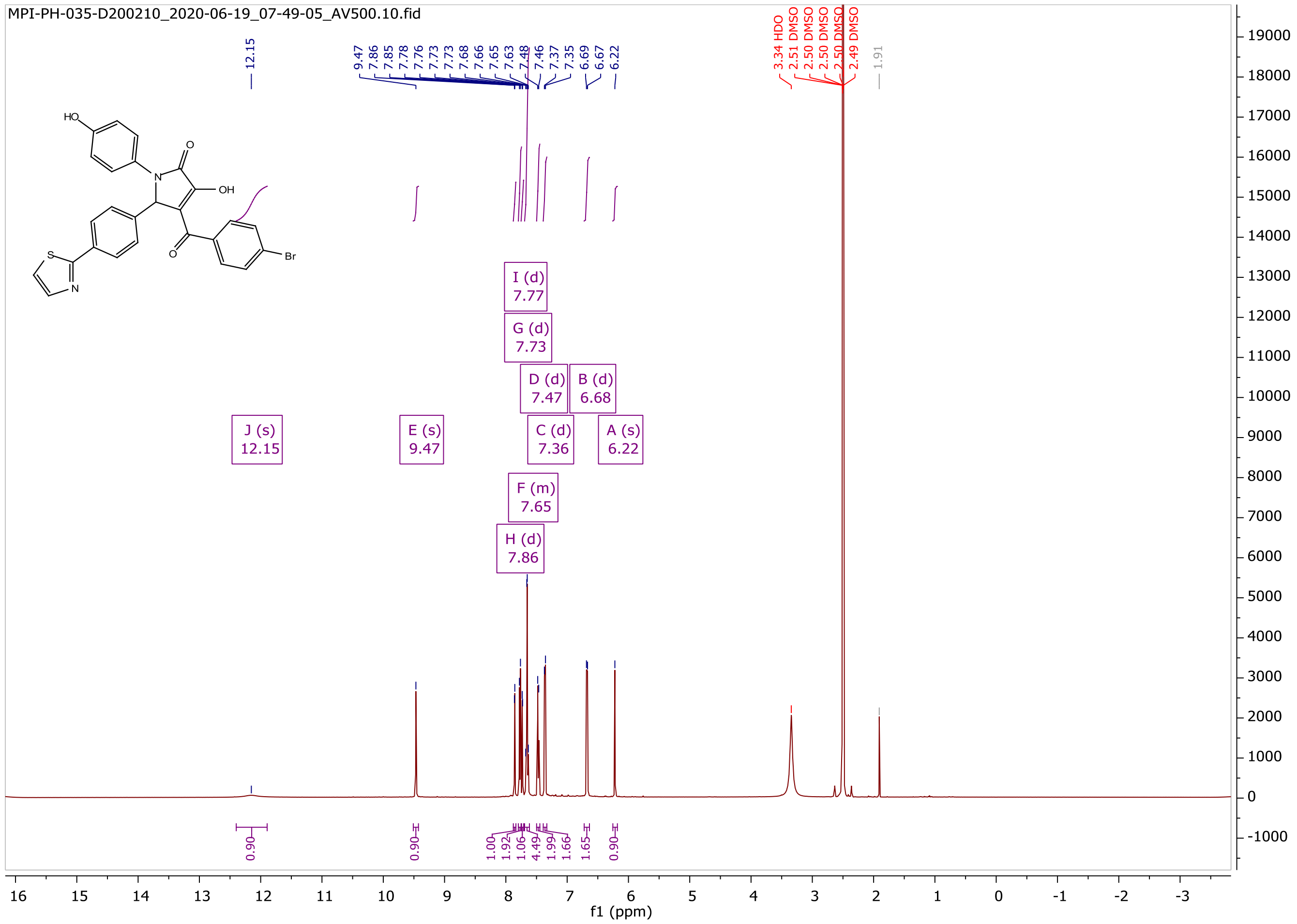

Partially enlarged ^{1}H NMR spectra of PH-35 (500 MHz, DMSO-d_6), 8 ppm to 6 ppm:

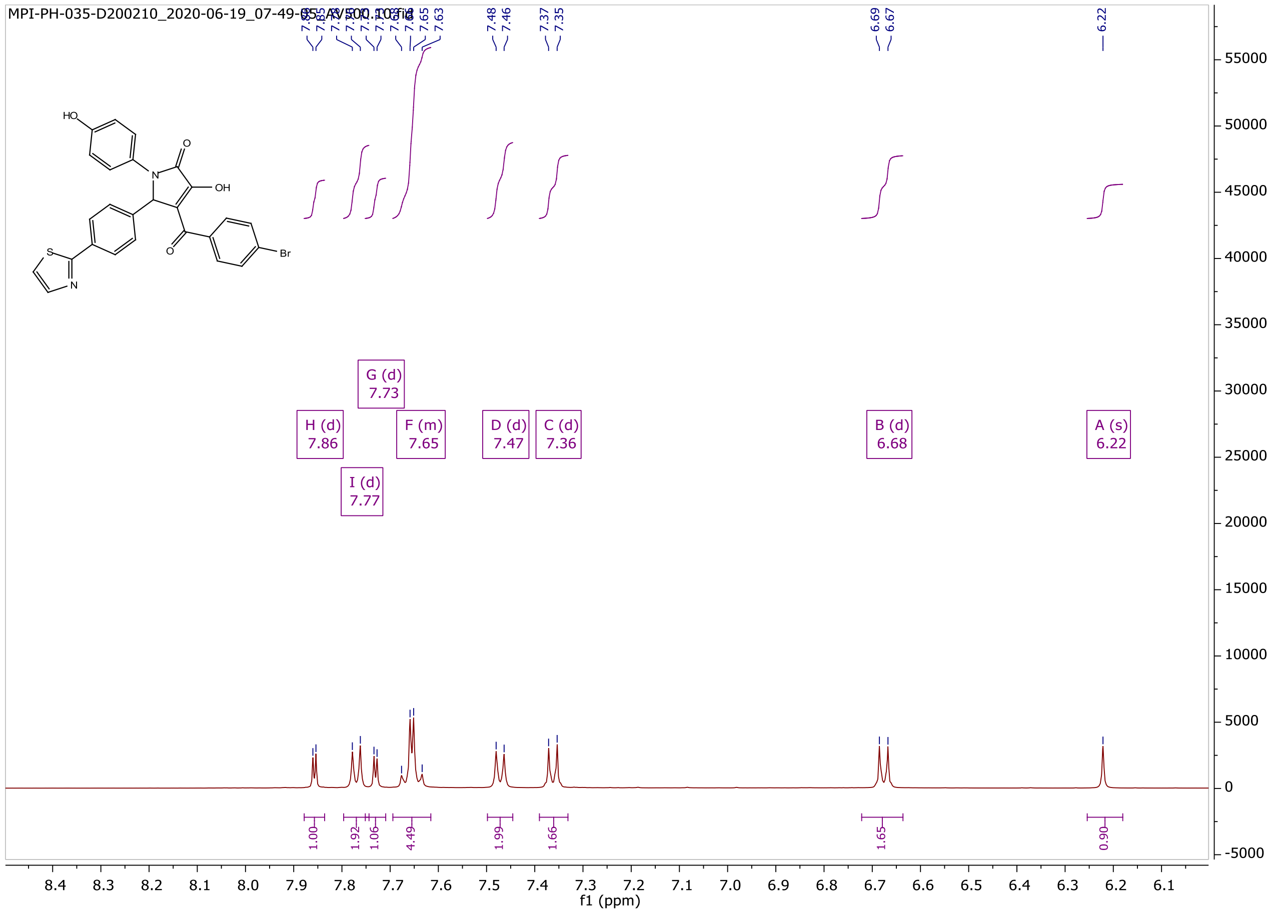

^{13}**C NMR of PH-35** (126 MHz, DMSO-d_6), 210 ppm to -10 ppm:

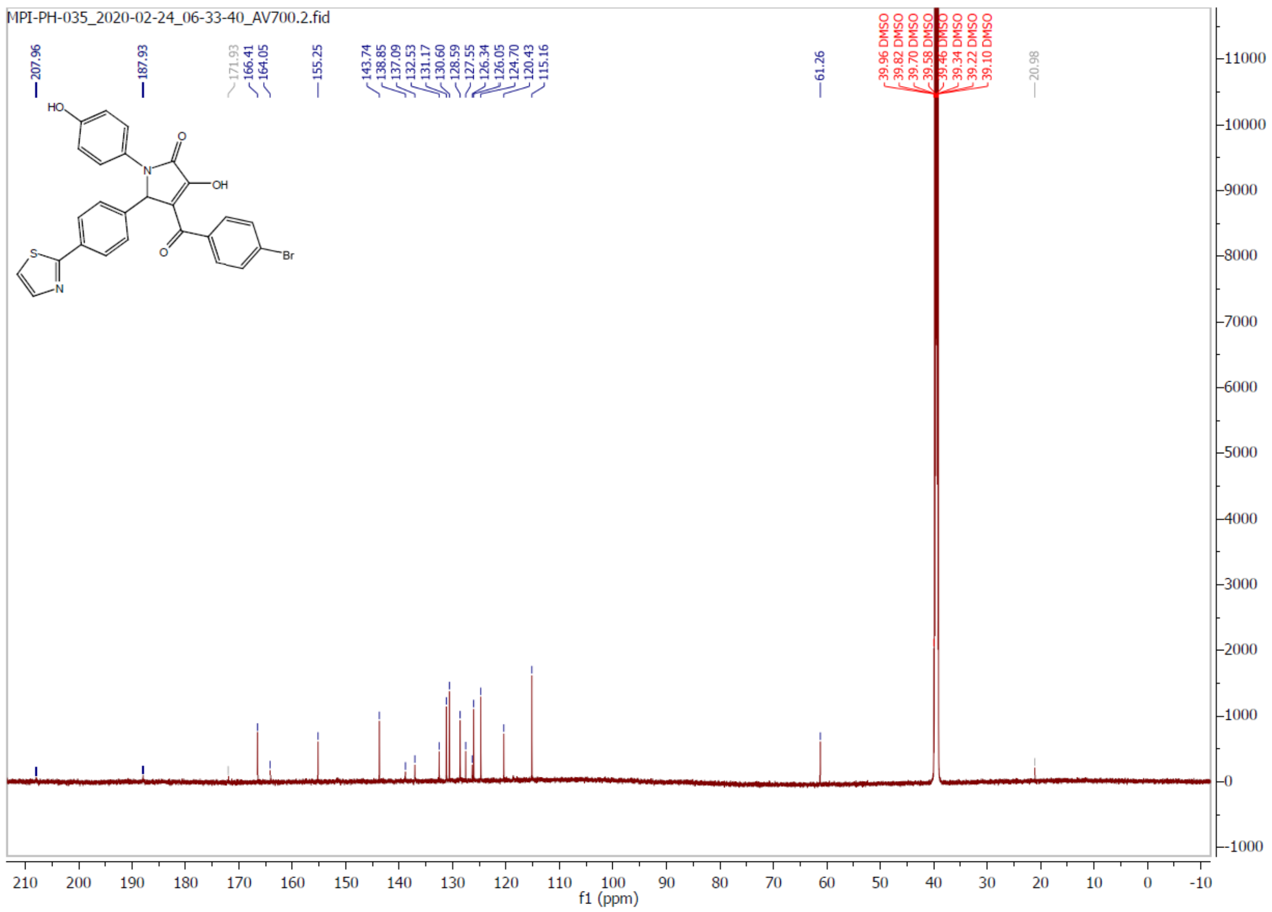

Analytical UHPLC, PH-36

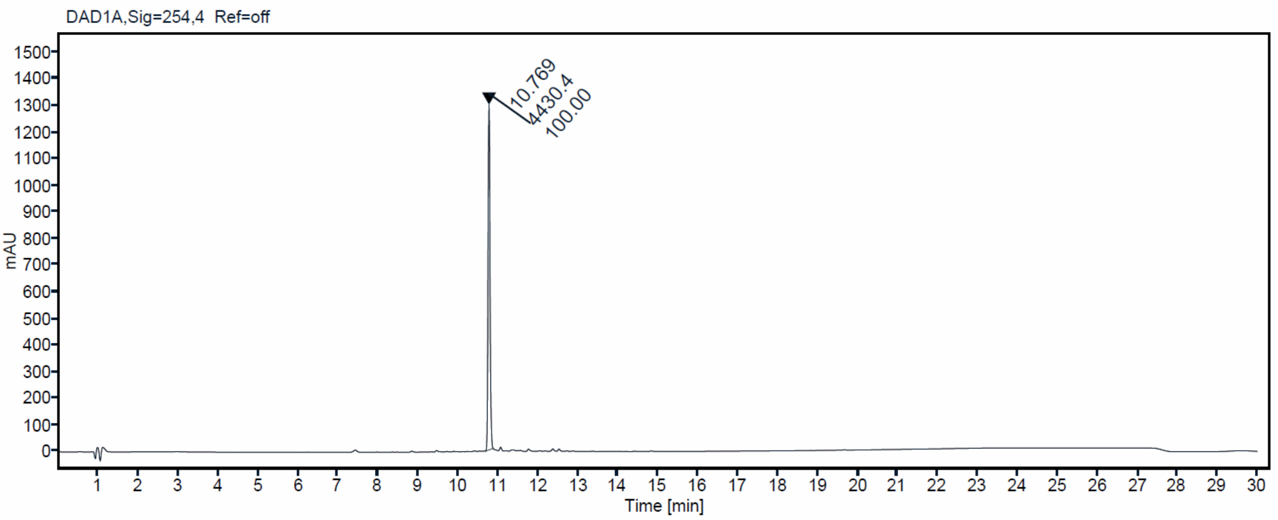

^{1}H NMR of PH-36 (600 MHz, DMSO-d_6), 15 ppm to -3 ppm:

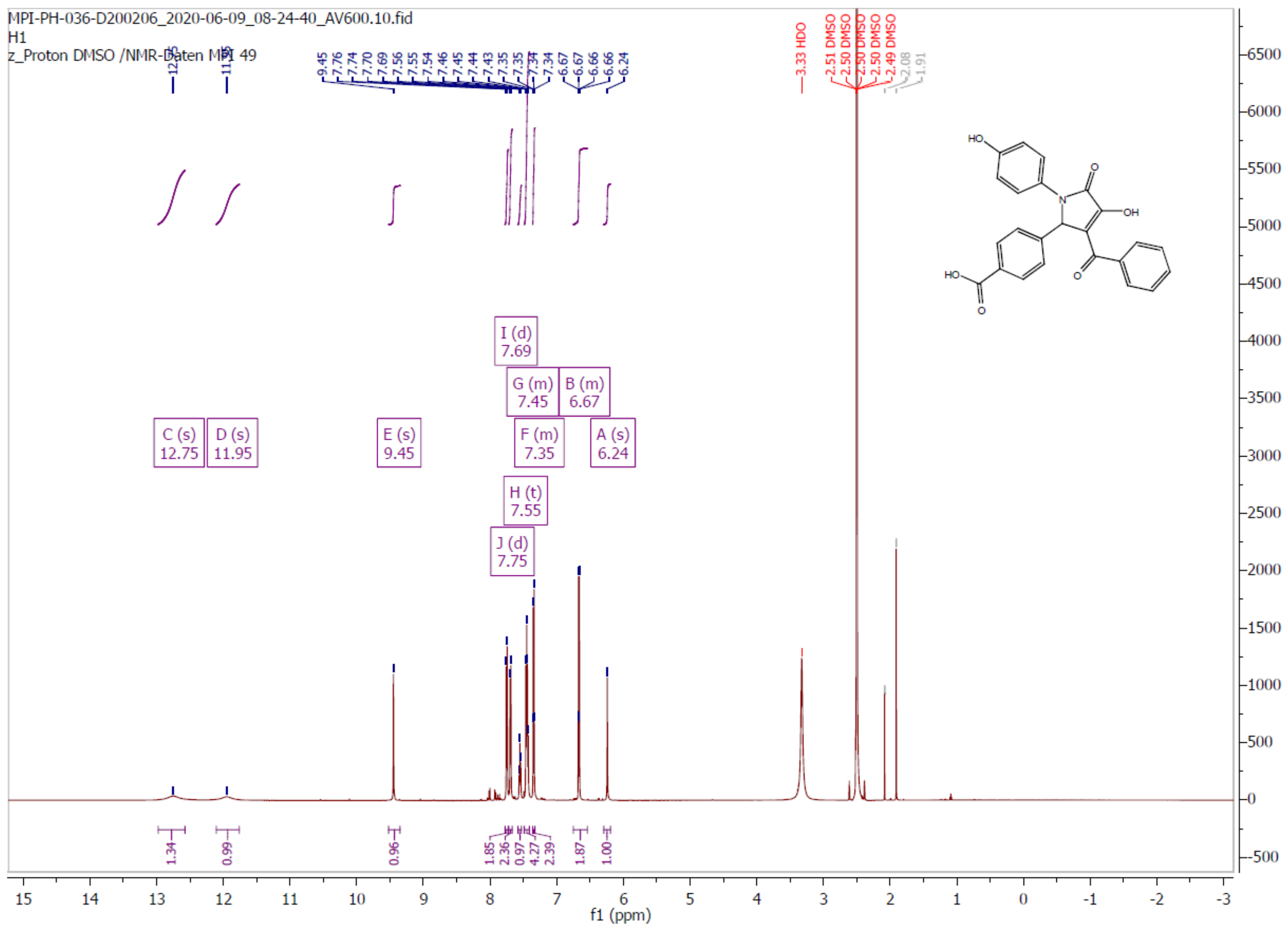

Partially enlarged ^{1}H NMR spectra of PH-36 (600 MHz, DMSO-d_6), 13 ppm to 6 ppm:

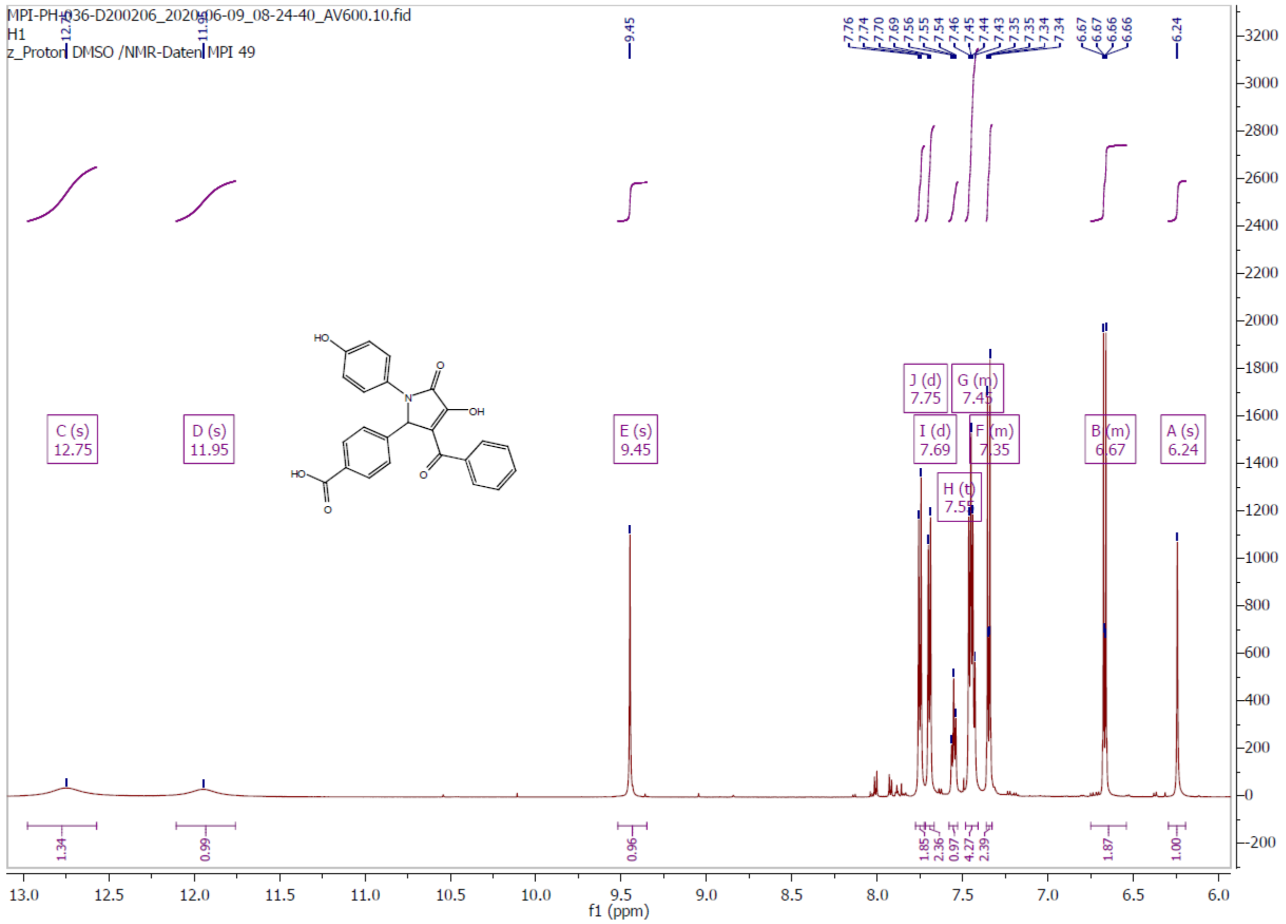

^{13}**C NMR of PH-36** (151 MHz, DMSO-d_6), 210 ppm to -10 ppm:

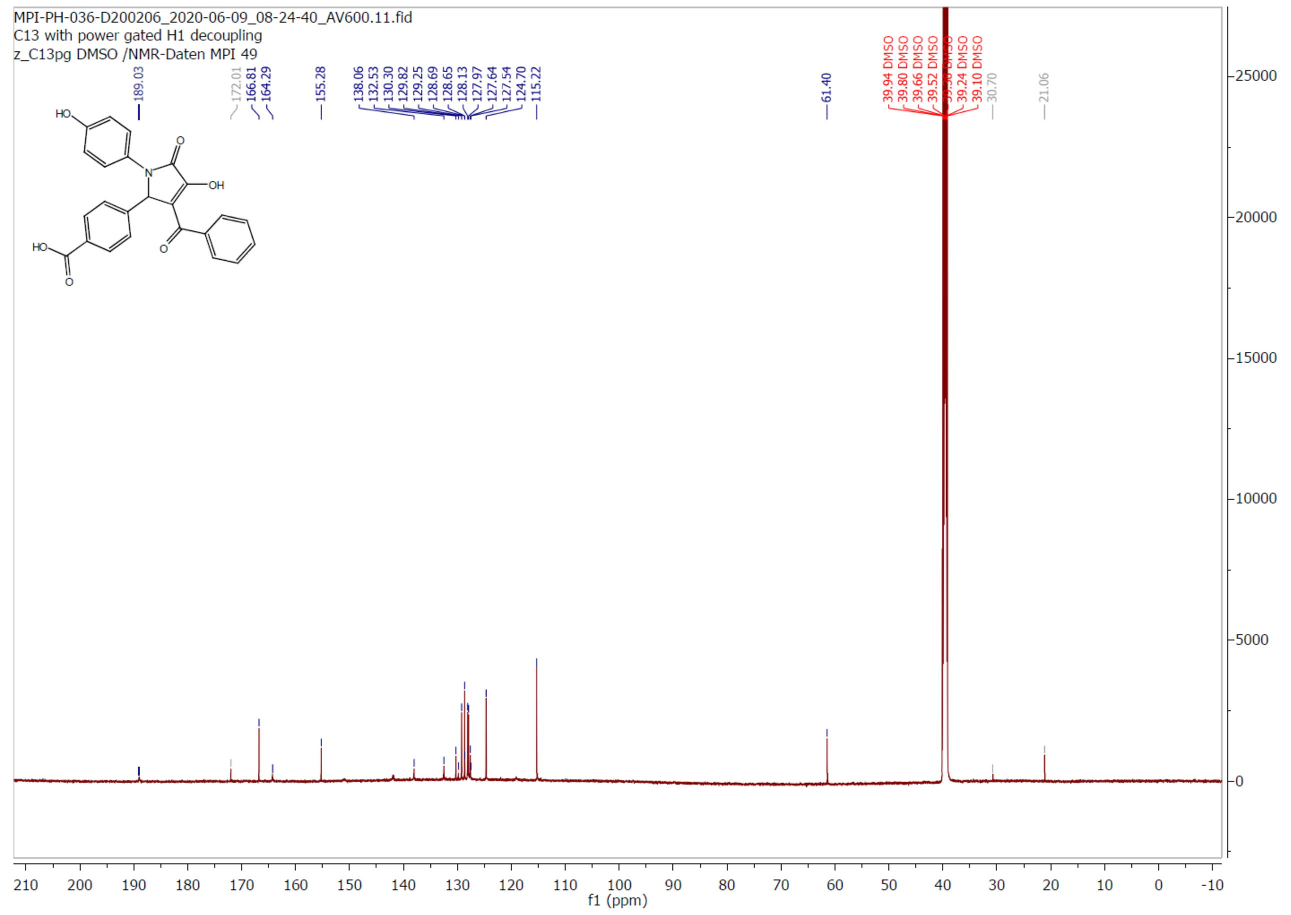

Analytical UHPLC, PH-37

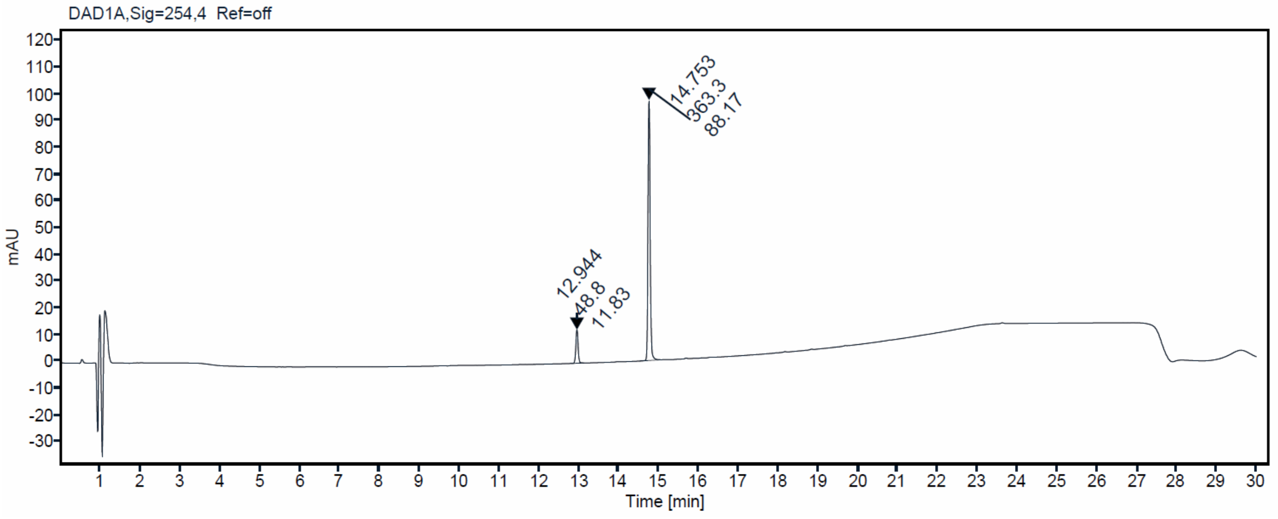

^{1}H NMR of PH-37 (600 MHz, DMSO-d_6), 16 ppm to -3 ppm:

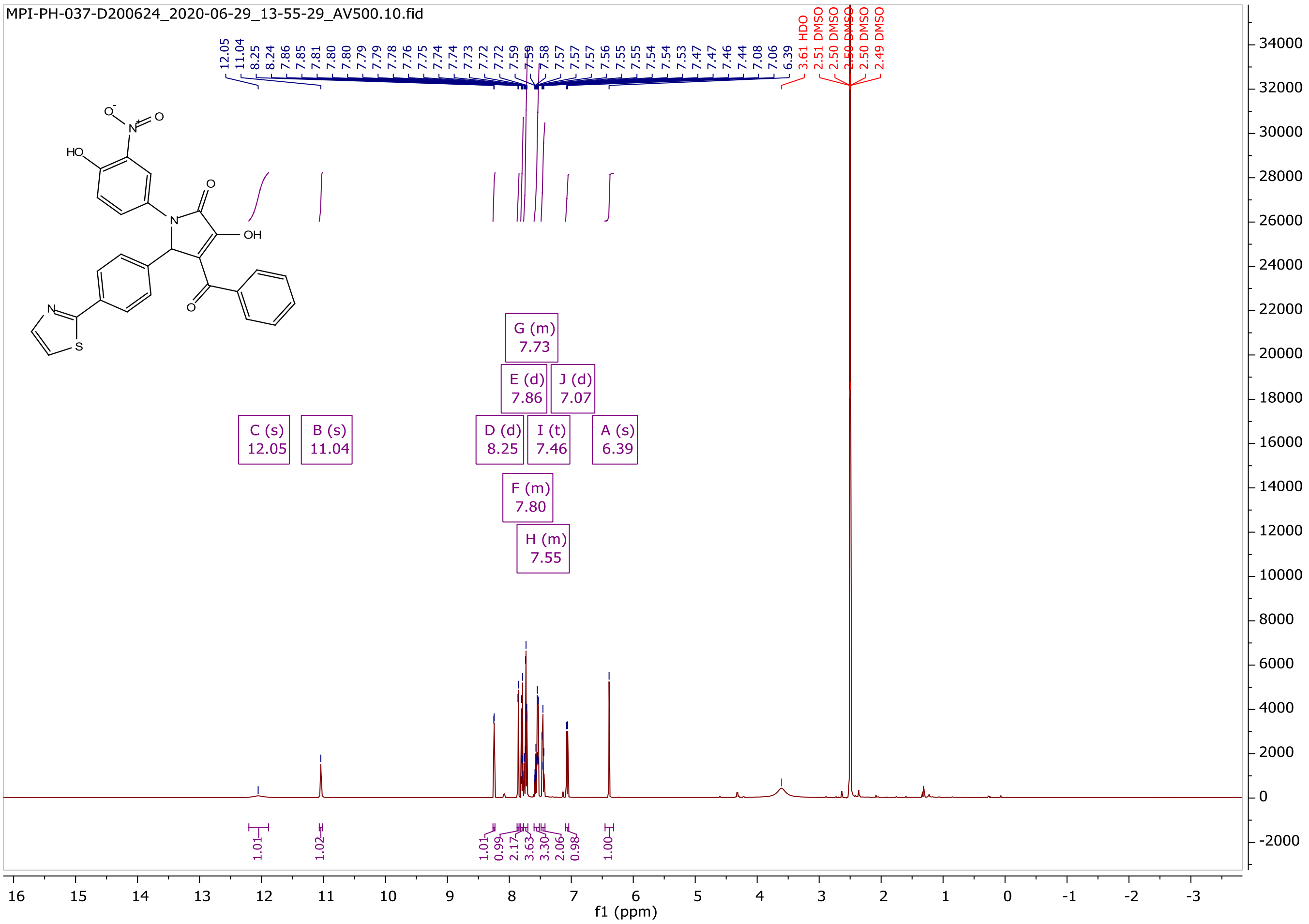

Partially enlarged ¹H NMR spectra of PH-37 (600 MHz, DMSO-d_6), 8.5 ppm to 6 ppm:

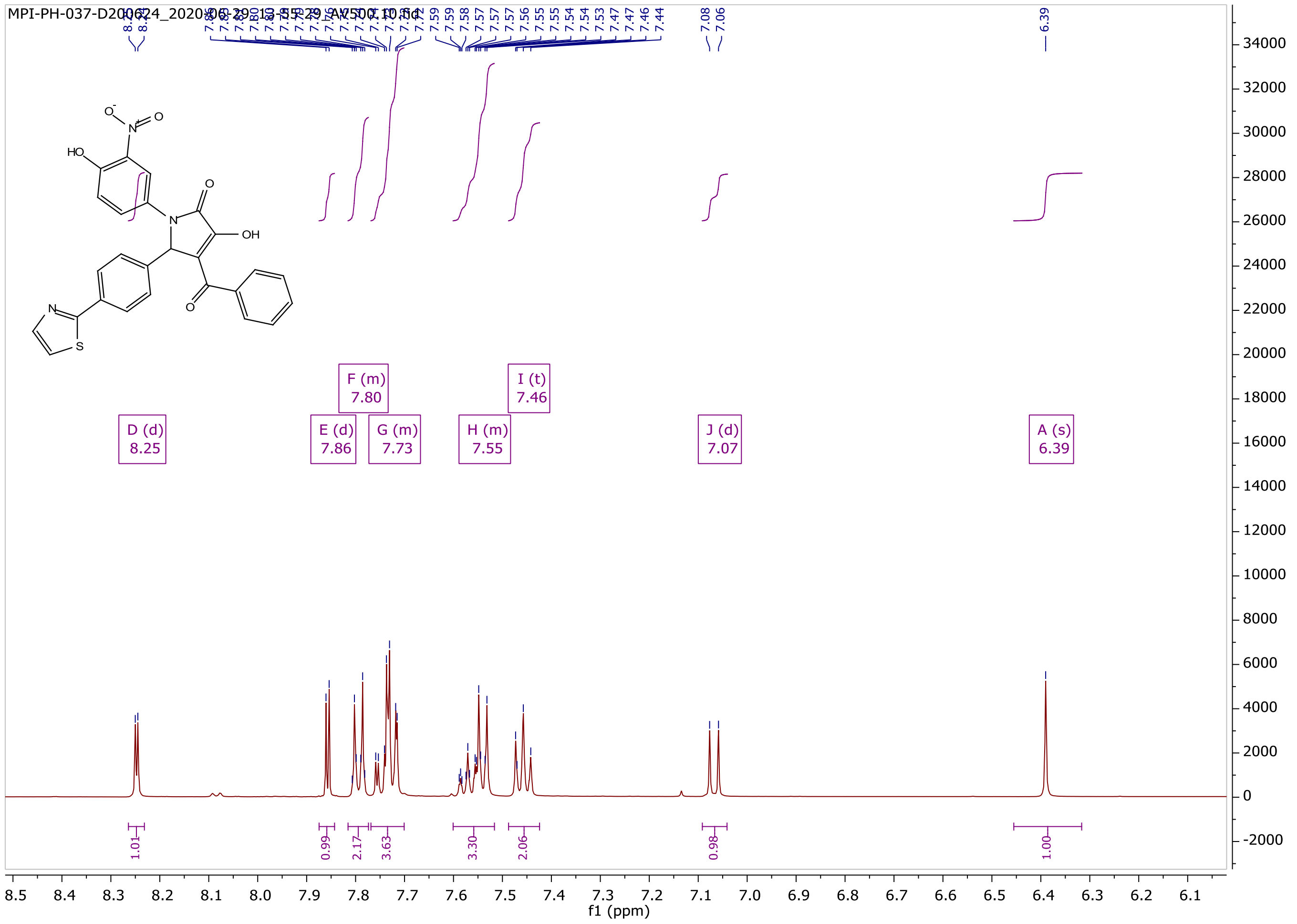

^{13}C NMR of PH-37 (126 MHz, DMSO-d_6), 210 to -10 ppm:

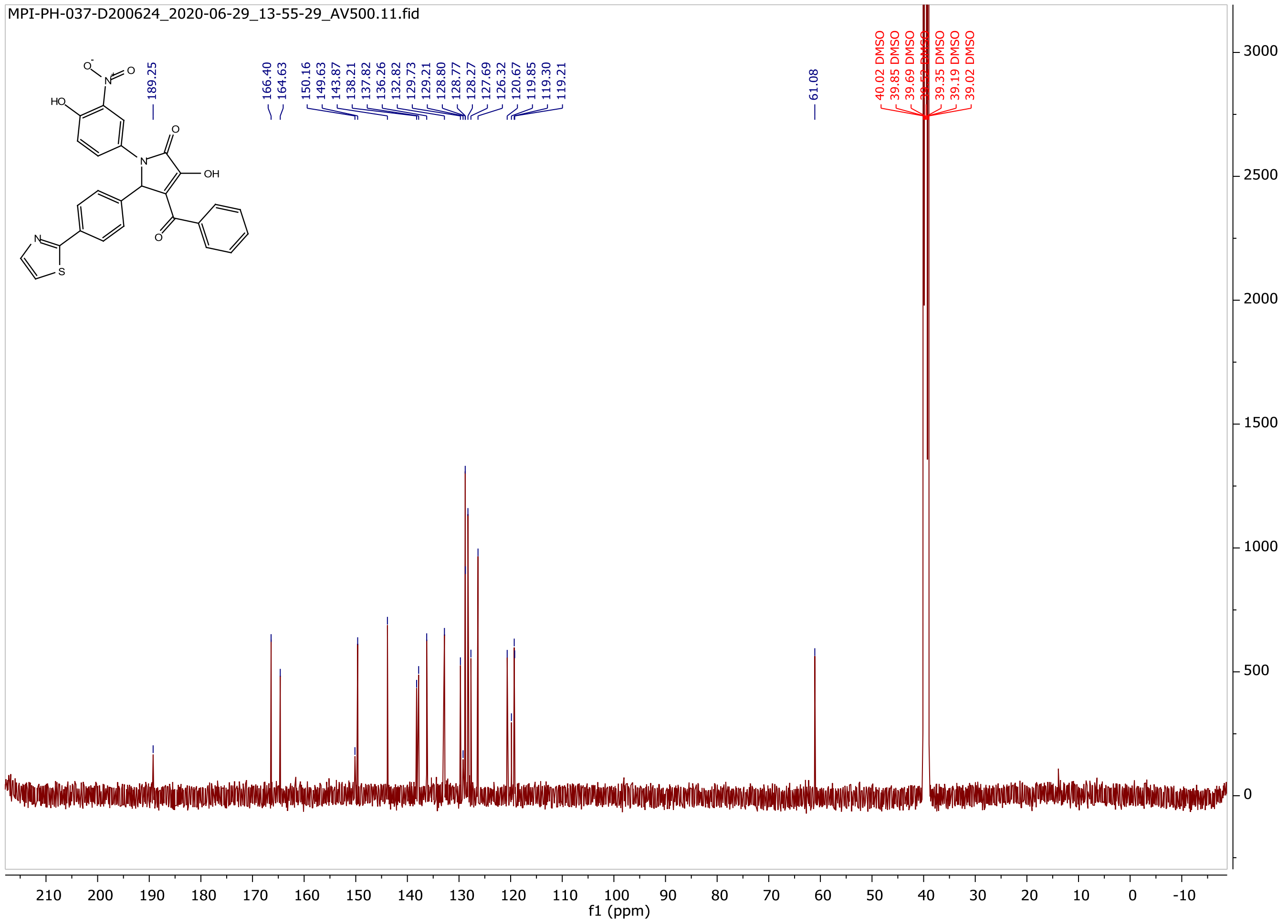

Analytical UHPLC, PH-38

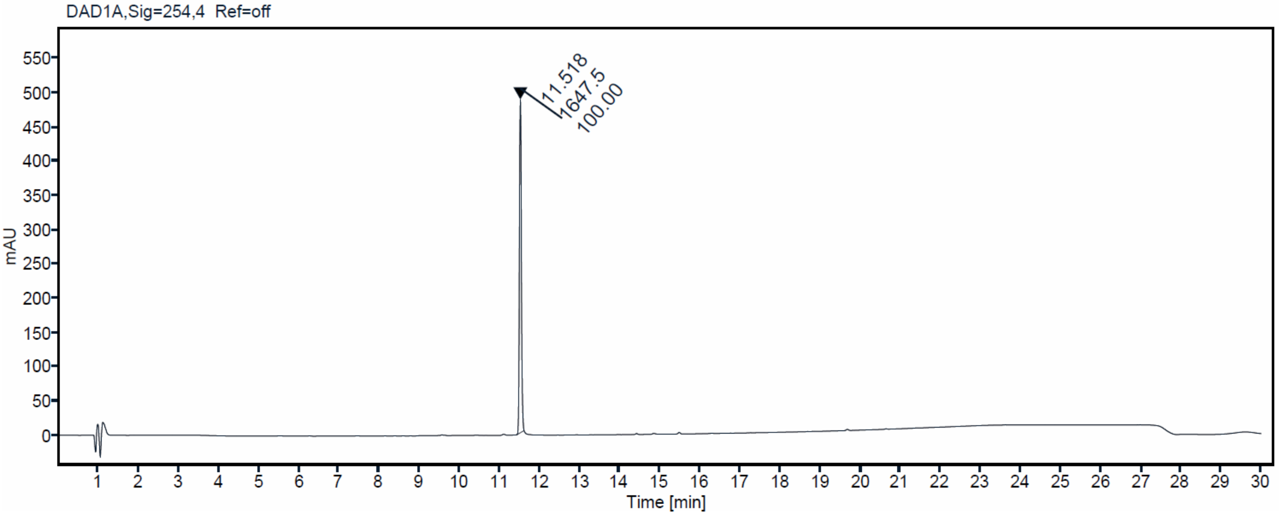

^{1}H NMR of PH-38 (500 MHz, DMSO-d_6), 16 ppm to -3 ppm:

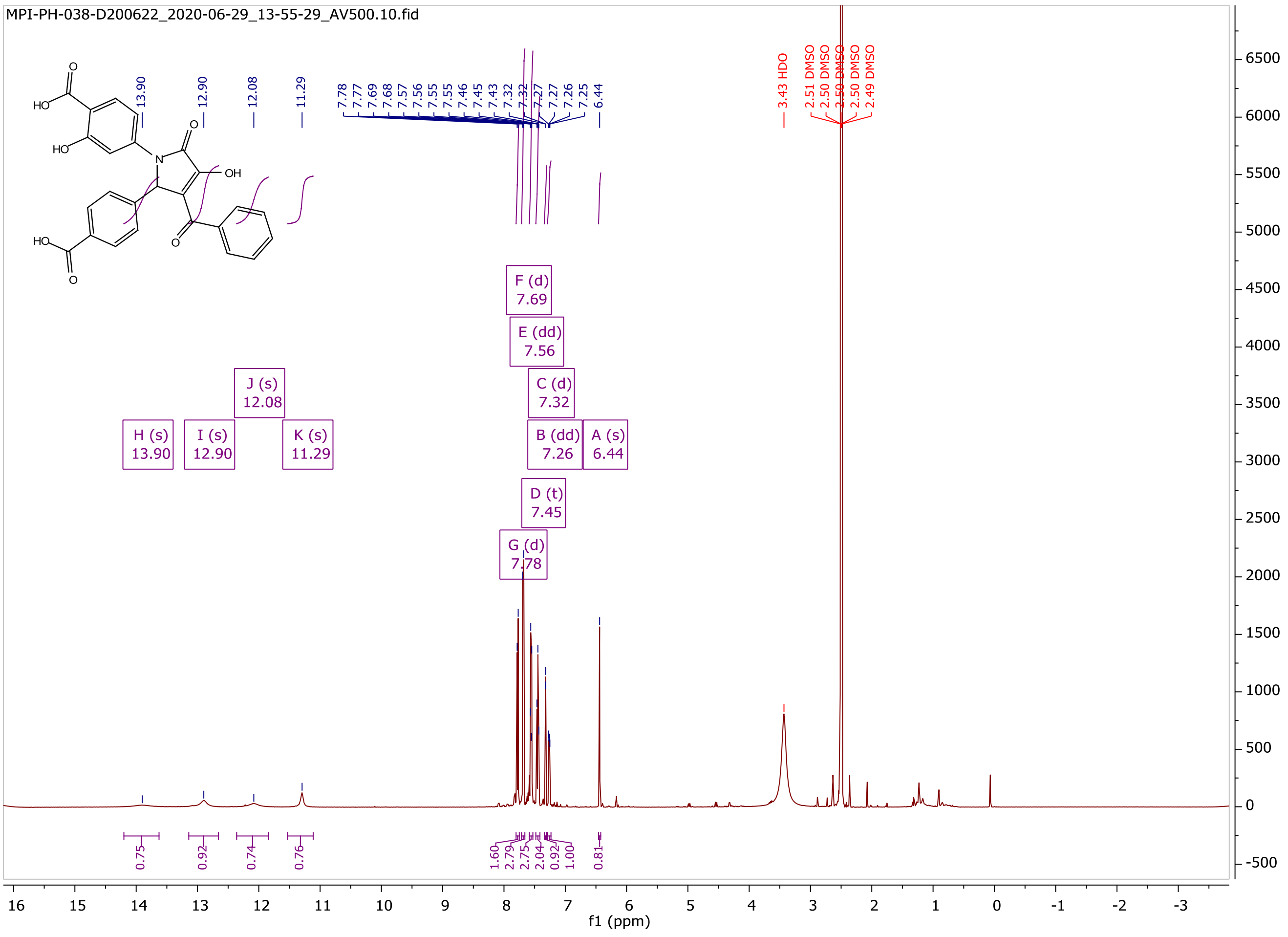

Partially enlarged ^{1}H NMR spectra of PH-38 (500 MHz, DMSO-d_6), 8.5 ppm to 6 ppm:

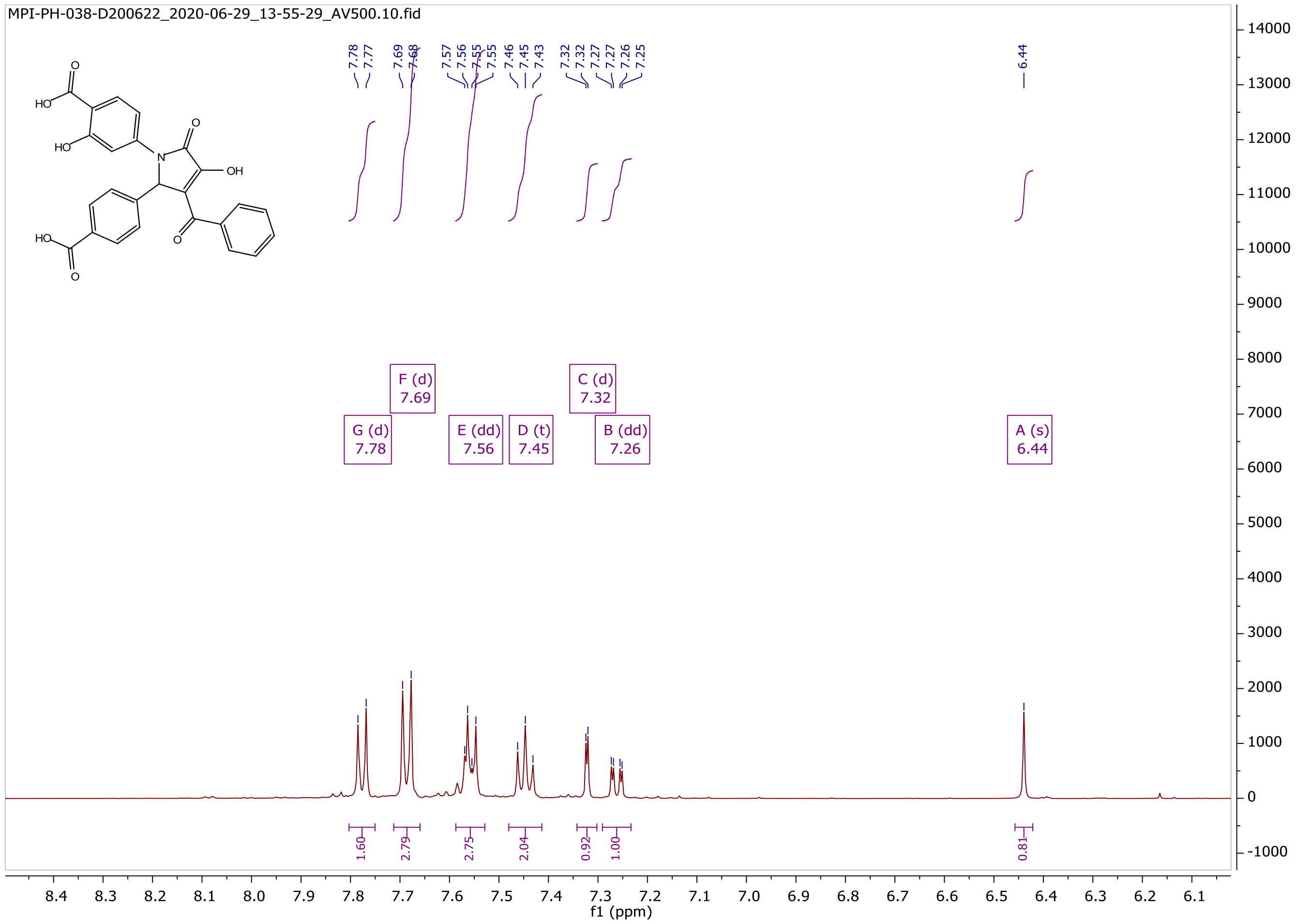

^{13}C NMR of PH-38 (126 MHz, DMSO-d_6), 210 ppm to -10 ppm:

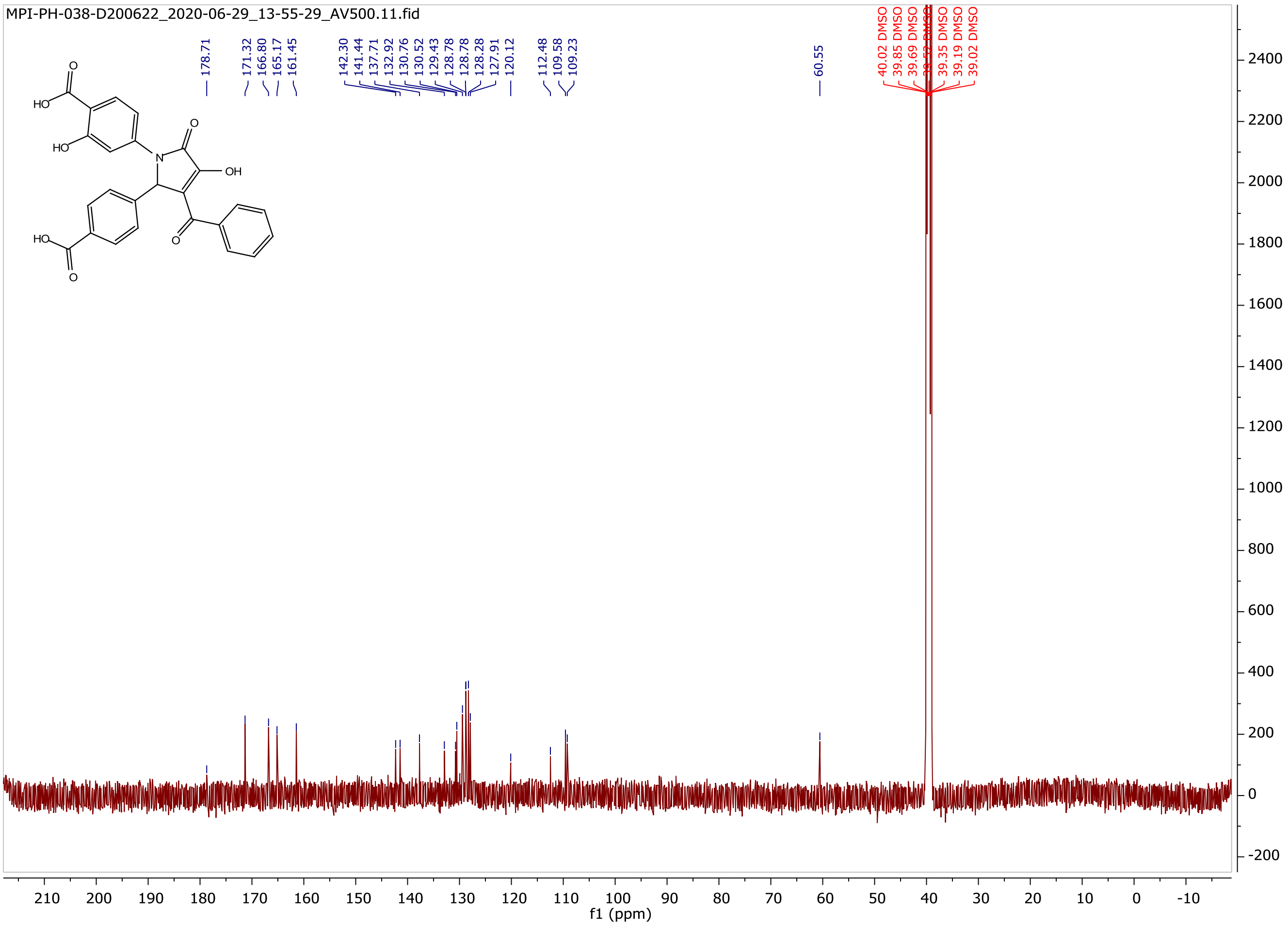

Analytical UHPLC, PH-39

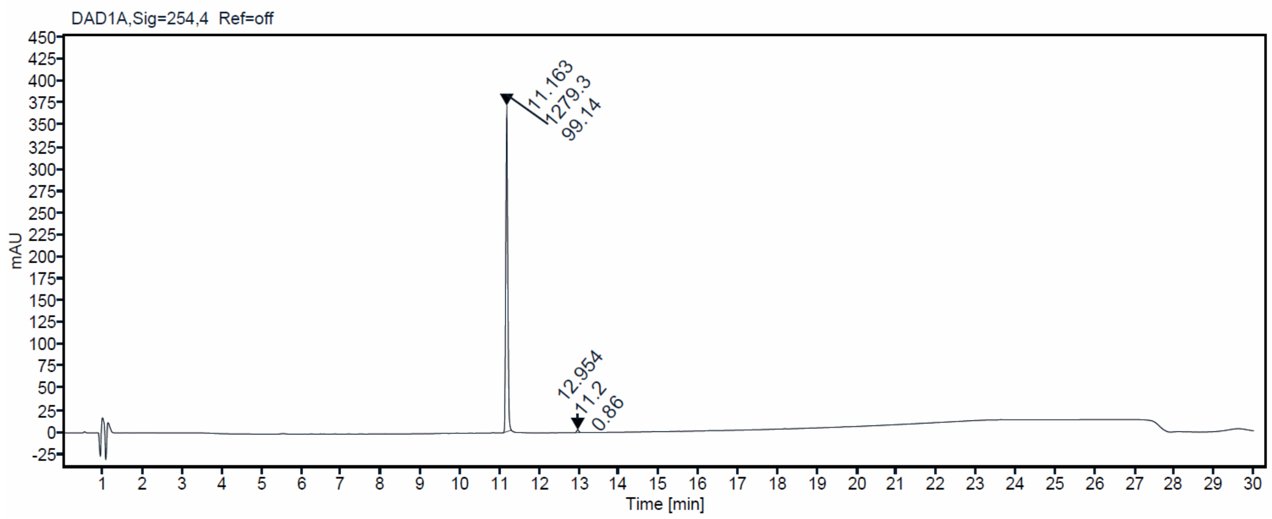

^{1}H NMR of PH-39 (500 MHz, Acetone-d_6), 15 ppm to -3 ppm:

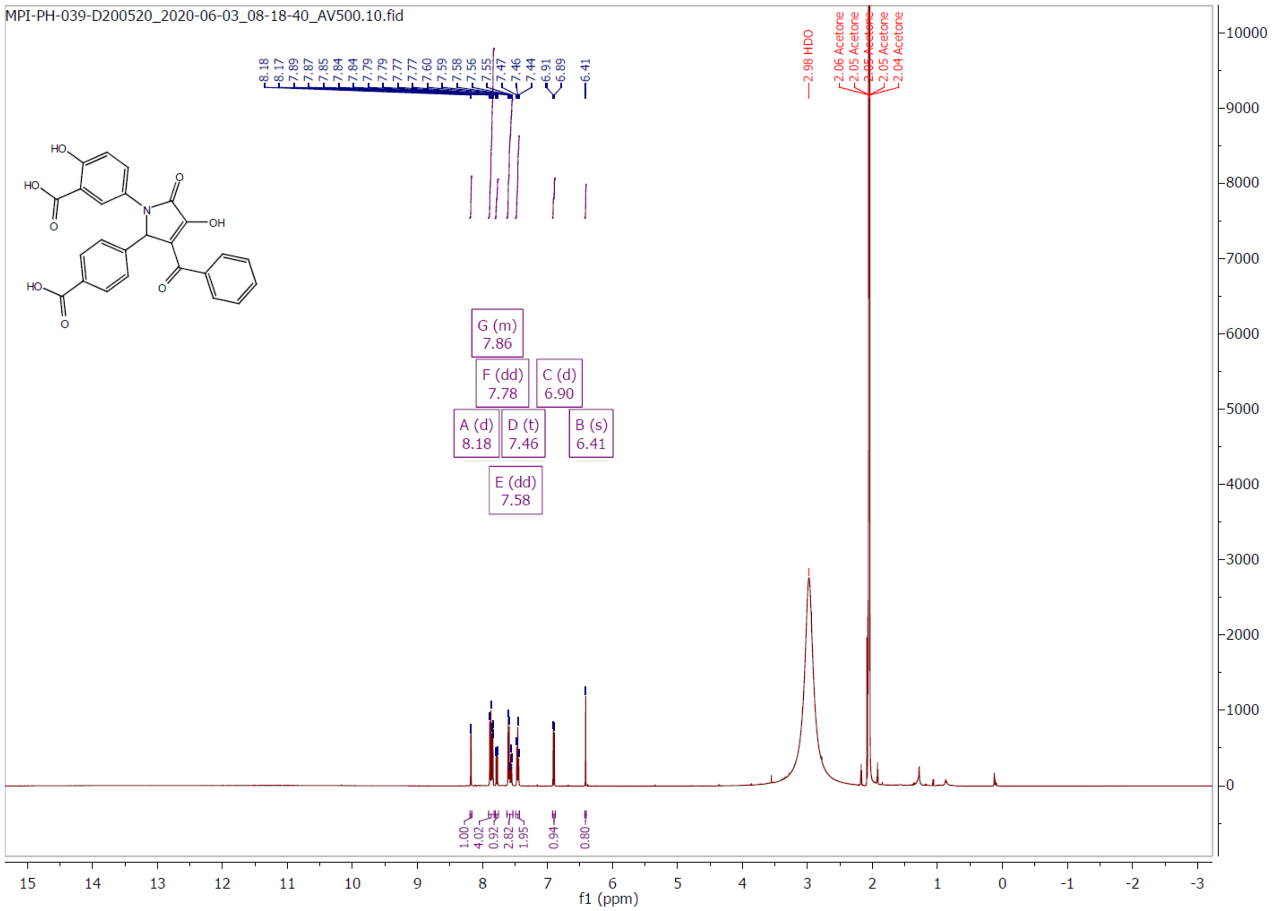

Partially enlarged ^{1}H NMR spectra of PH-39 (500 MHz, Acetone-d_6), 9 ppm to 6.0 ppm:

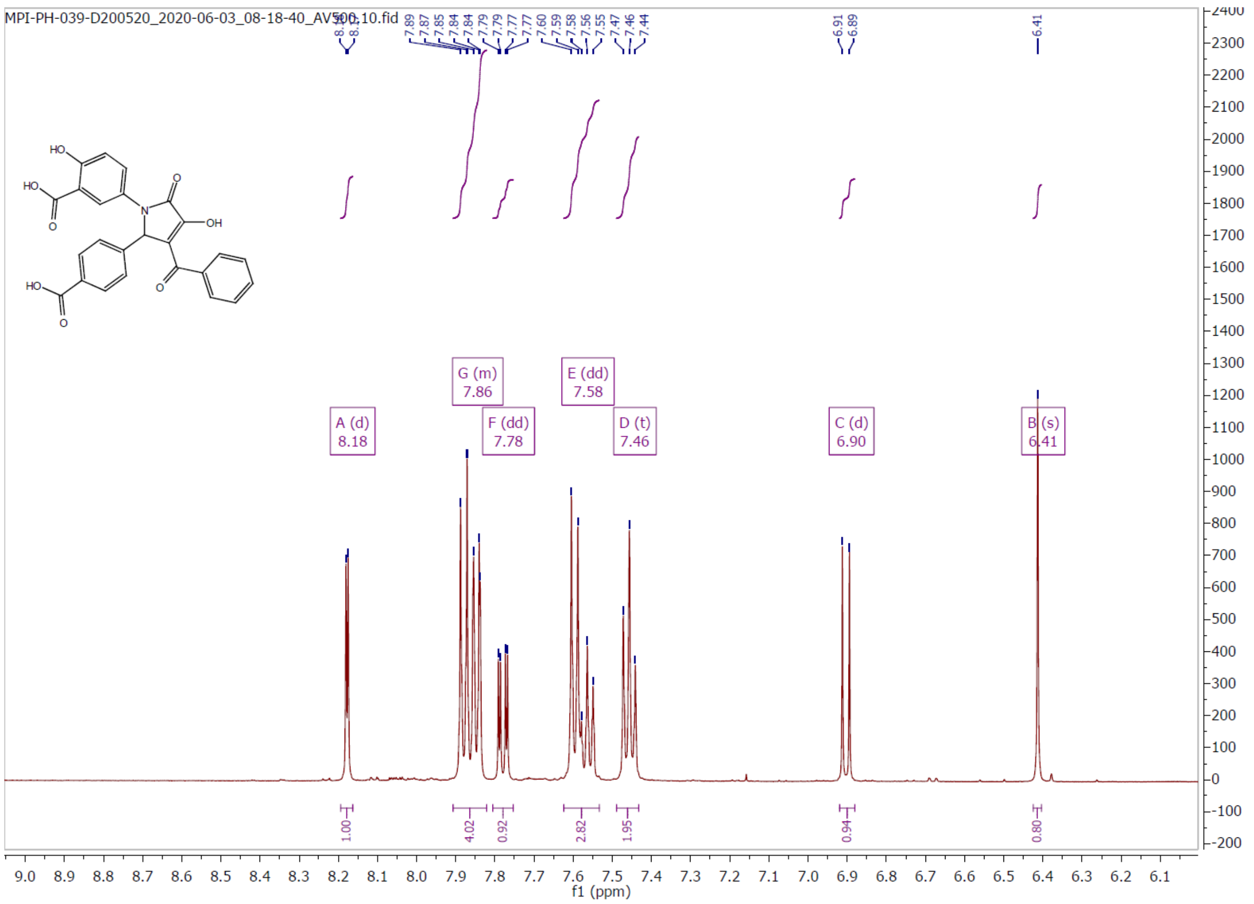

^{13}**C NMR of PH-39** (126 MHz, Acetone-d_6), 210 ppm to -10 ppm:

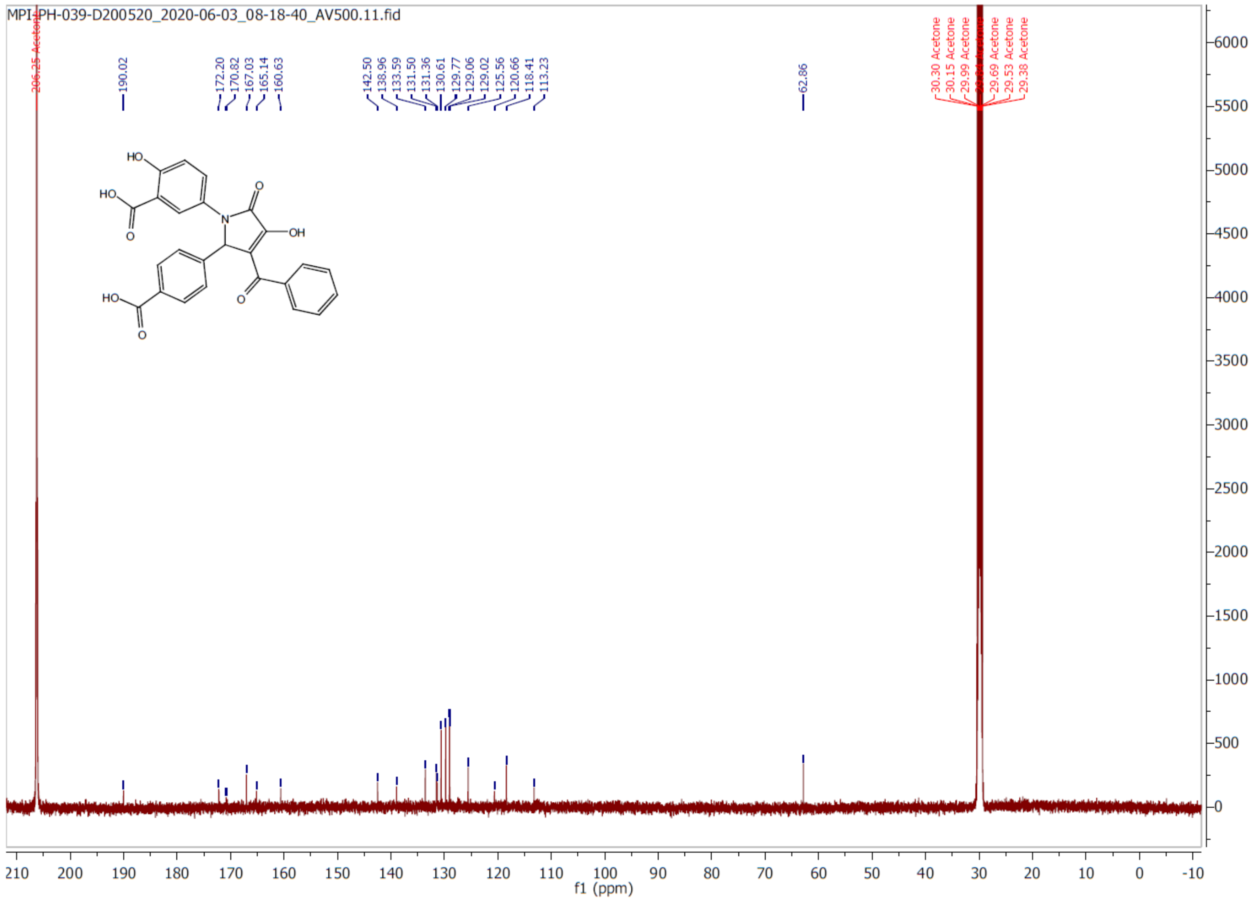

Analytical UHPLC, PH-43

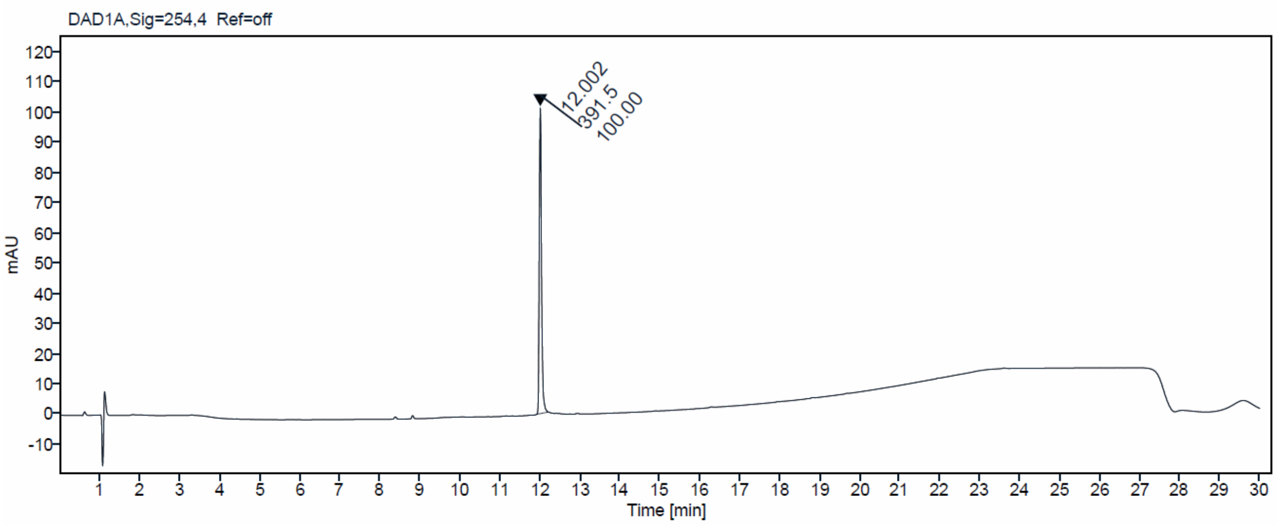

^{1}H NMR of PH-43 (600 MHz, Acetone-d_6), 15 ppm to -3 ppm:

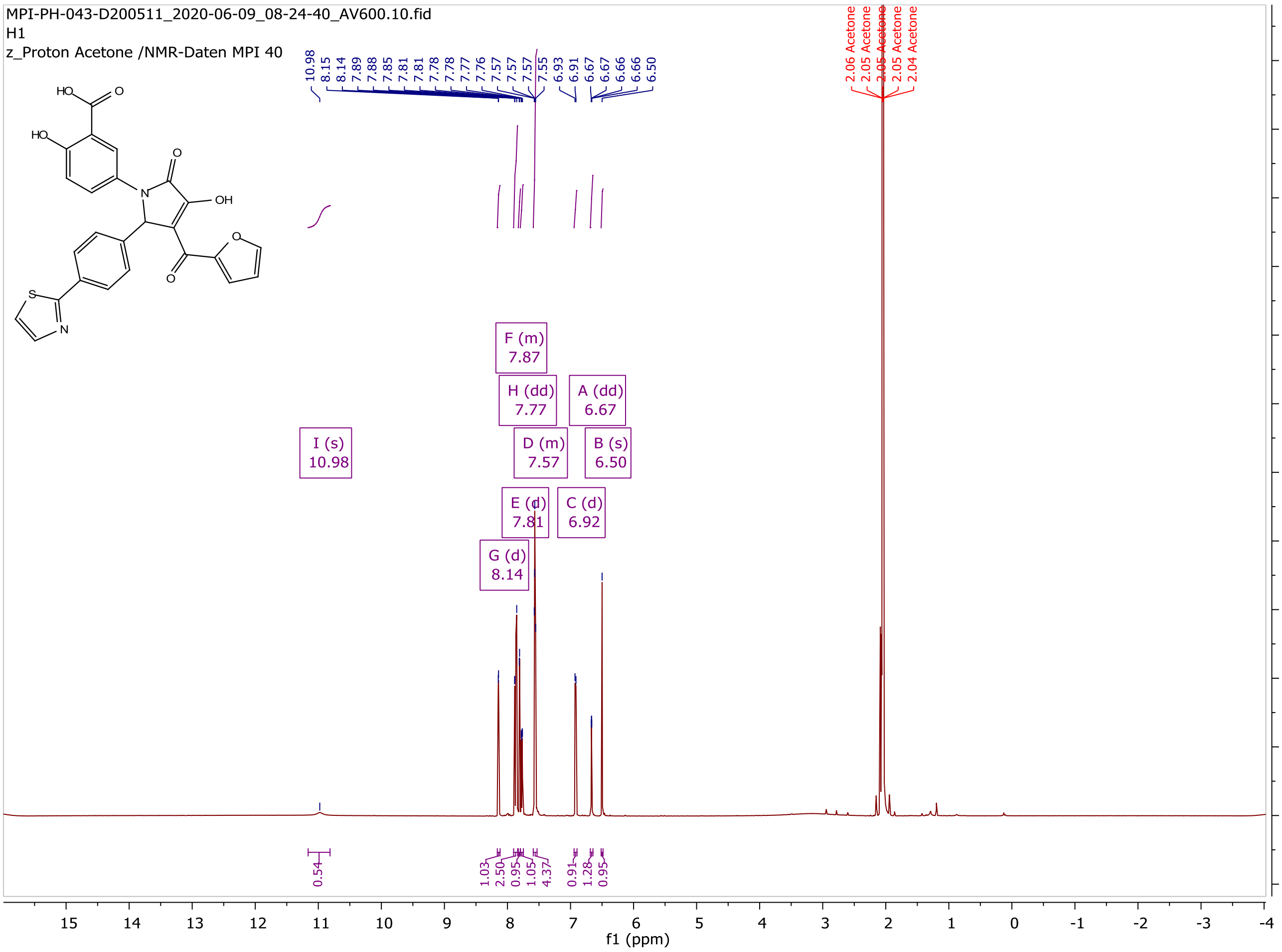

Partially enlarged ^{1}H NMR spectra of PH-43 (600 MHz, Acetone-d_6), 9 ppm to 6 ppm:

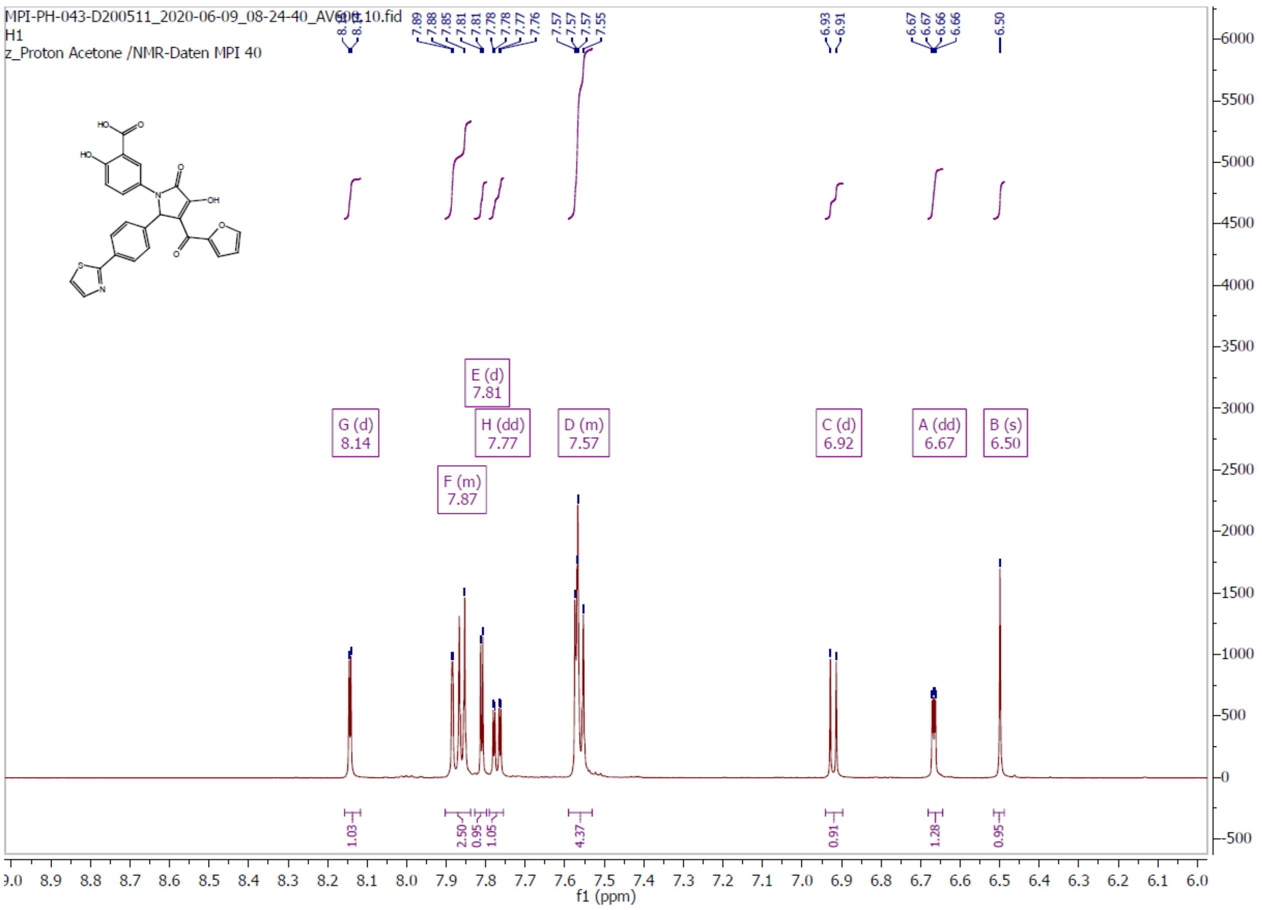

^{13}C NMR of PH-43 (151 MHz, Acetone-d_6), 210 ppm to -10 ppm:

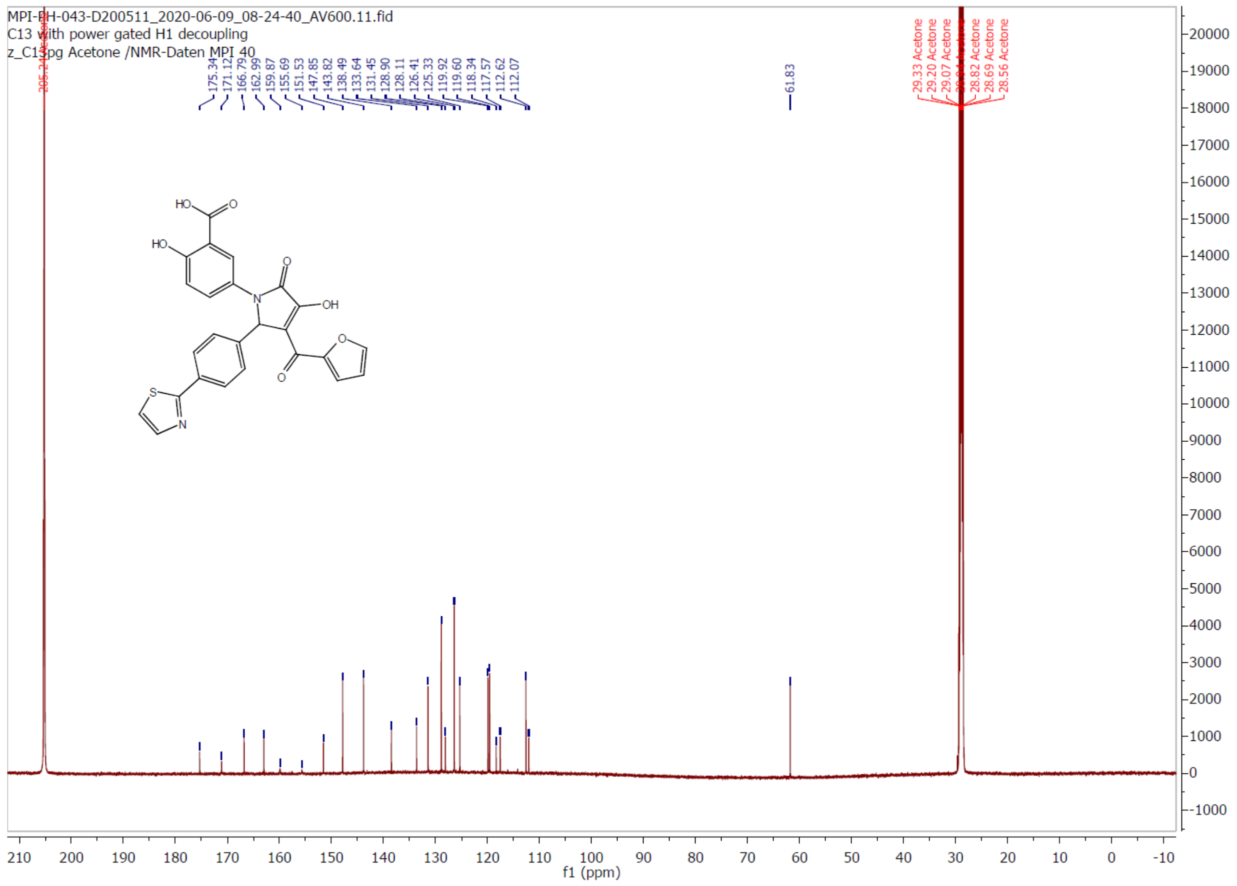

Analytical UHPLC, PH-44

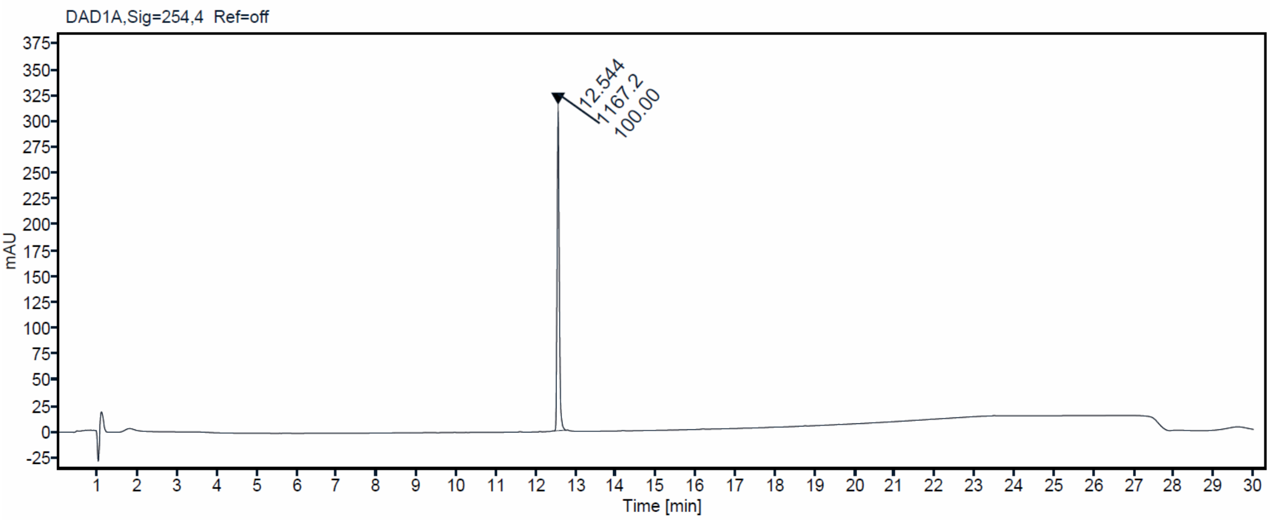

^{1}H NMR of PH-44 (600 MHz, Acetone-d_6), 15 ppm to -3 ppm:

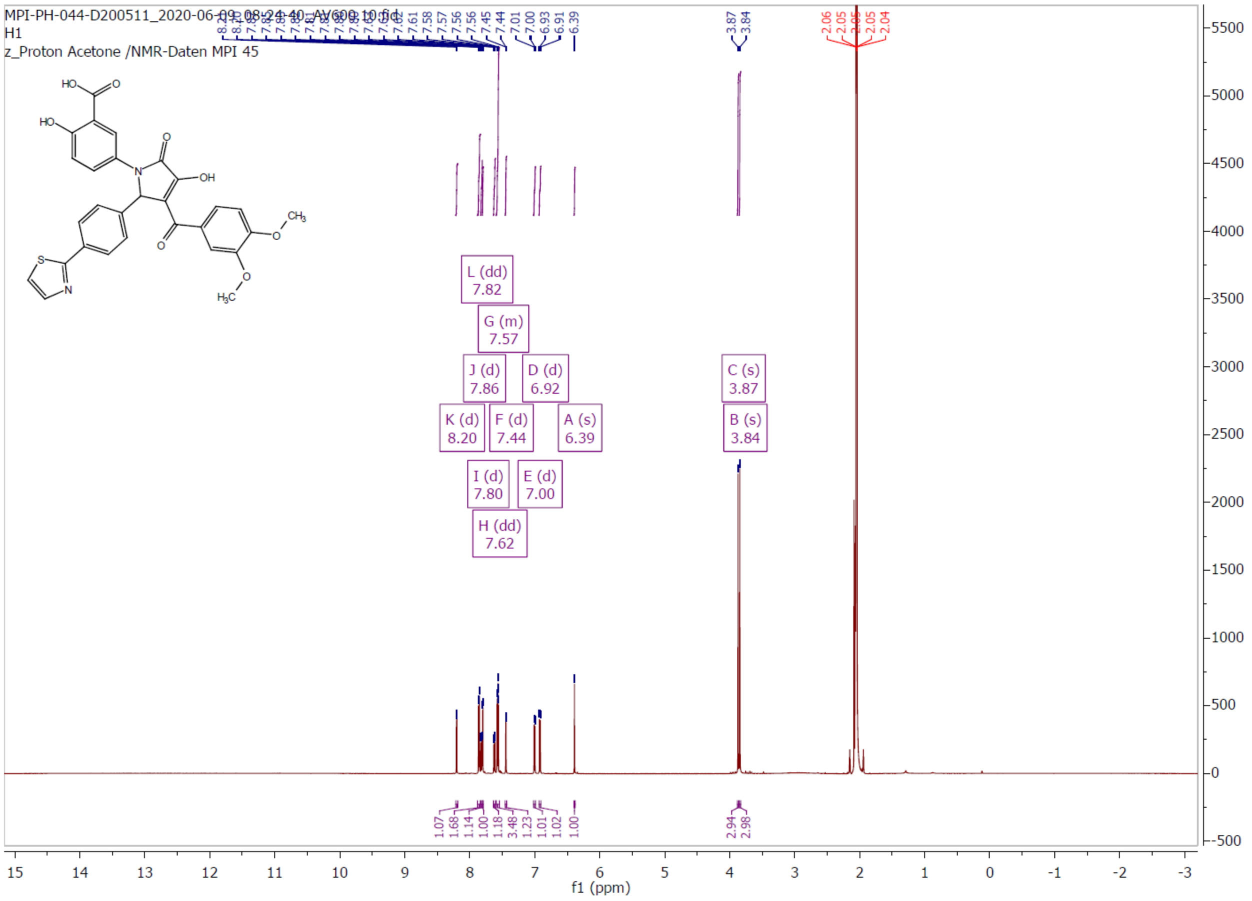

Partially enlarged ^{1}H NMR spectra of PH-44 (600 MHz, Acetone-d_6), 9 ppm to 6 ppm:

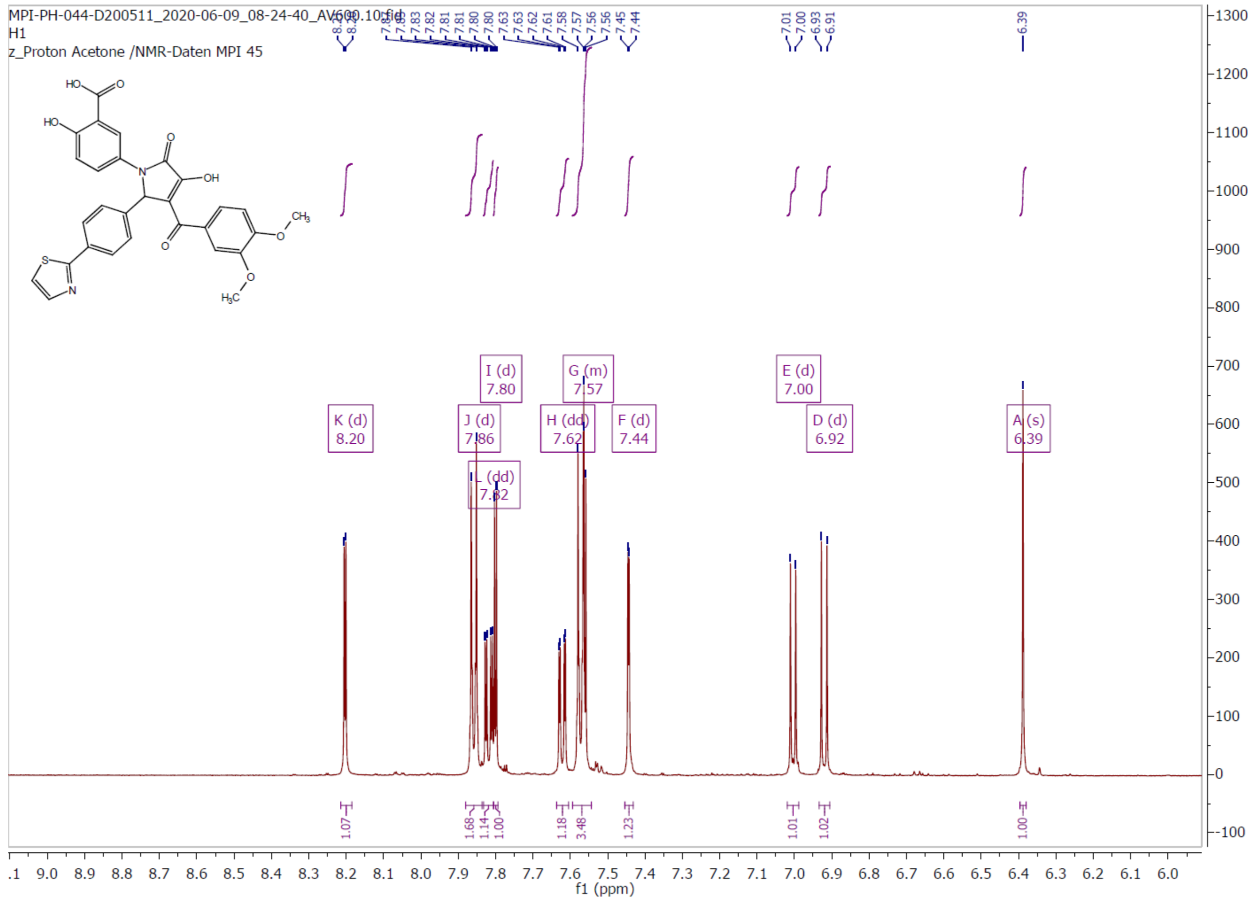

^{13}C NMR of PH-44 (151 MHz, Acetone-d_6), 200 ppm to -10 ppm:

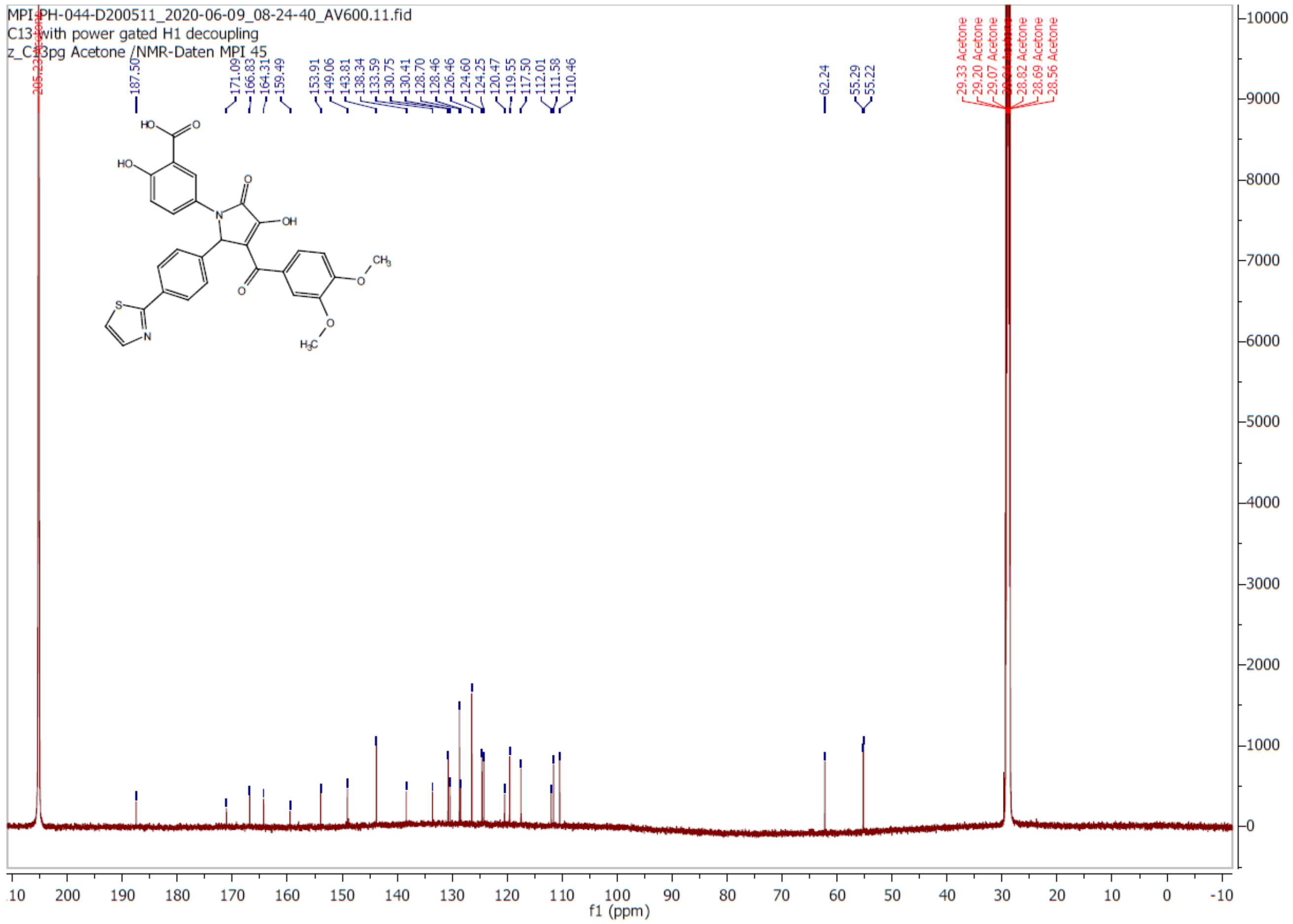

REFERENCES

1. Pattillo, R. A.; Ruckert, A.; Hussa, R. O.; Bernstein, R.; Delfs, E., The Jar cell line. Continuous human multihormone production and controls. *In Vitro* **1971,** *6*, 398-399.

2. Livak, K. J.; Schmittgen, T. D., Analysis of Relative Gene Expression Data Using Real-Time Quantitative PCR and the 2−ΔΔCT Method. *Methods* **2001,** *25* (4), 402-408.

3. Richter, A.; Rose, R.; Hedberg, C.; Waldmann, H.; Ottmann, C., An Optimised Small-Molecule Stabiliser of the 14-3-3–PMA2 Protein–Protein Interaction. *Chem. Eur. J.* **2012,** *18* (21), 6520-6527.
